# Polymeric immunoglobulin receptor deficiency exacerbates autoimmune hepatitis by inducing intestinal dysbiosis and barrier dysfunction

**DOI:** 10.1038/s41419-023-05589-3

**Published:** 2023-01-28

**Authors:** Hongwei Lin, Jing Lin, Tongtong Pan, Ting Li, Huimian Jiang, Yan Fang, Yuxin Wang, Faling Wu, Jia Huang, Huadong Zhang, Dazhi Chen, Yongping Chen

**Affiliations:** 1 Liver Disease Diagnosis and Treatment Center, The First Affiliated Hospital of Wenzhou Medical University, Hepatology Institute of Wenzhou Medical University, Wenzhou, 325000 Zhejiang China; 2 Zhejiang Provincial Key Laboratory for Accurate Diagnosis and Treatment of Chronic Liver Diseases, Wenzhou, 325000 Zhejiang China; 3 Hangzhou Medical College, Hangzhou, 310059 Zhejiang China

**Keywords:** Autoimmunity, Autoimmune diseases

## Abstract

Autoimmune hepatitis (AIH) is an immune-mediated inflammatory liver disease with unclear pathogenesis. The gut microbiota and intestinal barrier play an essential role in AIH. Polymeric immunoglobulin receptor (pIgR) is a central component of mucosal immunity. Herein, we aimed to test the hypothesis that pIgR plays a pivotal role in maintaining gut microbiota homeostasis and gut barrier integrity in an AIH mouse model. The expression of intestinal pIgR shows the variation tendency of falling after rising with the aggravation of experimental AIH (EAH). The deletion of *Pigr* exacerbates liver damage in EAH. Furthermore, we identified a distinct microbiota profile of *Pigr*-deficient EAH mice, with a significant increased aboundance in the *Oscillospiraceae* family, particularly the *Anaeromassilibacillus* genus. Such a situation occurs because the loss of *Pigr* inhibits MEK/ERK, a key signal pathway whereby pIgR transports immunoglobulin A (IgA), resulting in reduced IgA secretion, which leads to the destruction of intestinal epithelial tight junction proteins and intestinal flora disturbance. Increased intestinal leakage causes increased translocation of bacteria to the liver, thus aggravating liver inflammation in EAH. Treatment with the *Lactobacillus rhamnosus* GG supernatant reverses liver damage in EAH mice but loses its protective effect without pIgR. Our study identifies that intestinal pIgR is a critical regulator of the adaptive response to S100-induced alterations in gut flora and the gut barrier function, which closely correlates with liver injury. Intestinal upregulation of pIgR could be a novel approach for treating AIH.

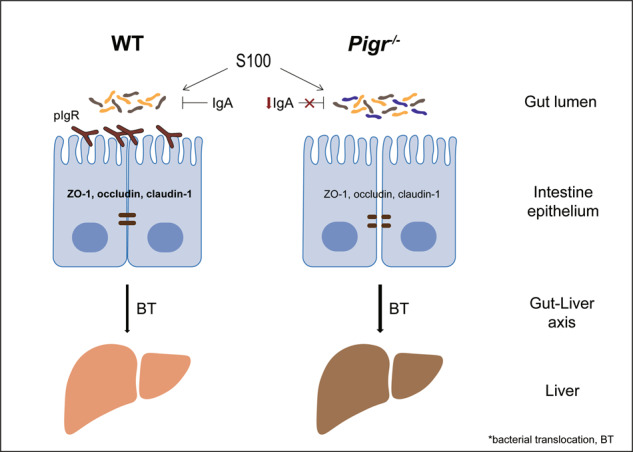

## Introduction

Autoimmune hepatitis (AIH) is a complex immune-mediated disease with chronic progressive inflammation characterized by elevated serum transaminases, hypergammaglobulinemia, and positive autoantibodies [1]. AIH is common in middle-aged women, and its incidence has increased rapidly in recent years [2]. The etiology of AIH is unclear; however, genetic and environmental factors are known to be involved [3, 4]. In addition, there is accumulating evidence indicating that alterations in the gut microbiome are associated with almost all liver or immunological diseases [3]. Disruption of the gut barrier function increases gut permeability, leading to translocation of gut-derived bacteria, thereby stimulating the innate immune system and damaging the liver [5, 6].

The gut barrier function is vital in preventing increased bacterial translocation [7–9]. Immunoglobulin A (IgA) is the main primary component of the mucosa and plays a key role in intestinal mucosal immunity and the maintenance of intestinal homeostasis by killing or inhibiting bacteria, neutralizing viral toxins, and regulating intestinal flora colonization and growth [10–12]. Most IgA-secreting plasma cells are located in the mucosa-associated lymphoid tissue of the gut and secrete IgA dimers linked by J chains [13]. IgA transcytosis, which is mediated by polymeric immunoglobulin receptor (pIgR), is an essential component of mucosal immunity [14]. PIgR is a single transmembrane protein that possesses extracellular, transmembrane, and intracytoplasmic domains. The extracellular ligand-binding region, termed the secretory component, is hydrolyzed and released as a secretory IgA (sIgA) component or in a free form. The secretory component has innate antimicrobial properties and defends sIgA against degradation [15]. By reinforcing the intestinal epithelial barrier, sIgA suppresses inappropriate immune activation by enteric microorganisms and antigens [16]. The paracellular space of the intestinal epithelium is sealed by a tight junction (TJ), composed of multiple protein complexes, including Claudin-1, ZO-1, and Occludin [17]. Extracellular signal-regulated kinase (ERK) is the most critical extracellular signal transduction pathway of the mitogen-activated protein kinase (MAPK) family [18]. ERK is stimulated by various extracellular factors and activated or inactivated upon phosphorylation. ERK1 and ERK2 are activated by MAPK/ERK kinase (MEK) 1 or 2 [19]. Researchers have shown that the MEK/ERK is a key pathway in the endocytosis of IgA-pIgR [20].

*Lactobacillus rhamnosus GG* (LGG), the most studied probiotic strain in the world, has been extensively used to prevent and treat liver diseases in humans and animal models [21–23]. Moreover, previous studies have shown that LGG culture supernatant (LGG-s) can protect against gut-derived liver disorders [24, 25]. Additionally, as one of the main metabolites of bacteria, short-chain fatty acids (SCFAs) have been confirmed to be closely related to IgA production [26, 27].

In this study, we identified that intestinal pIgR is a key regulator of AIH. *Pigr* deficiency exacerbated S100-induced experimental AIH (EAH) by reducing intestinal sIgA levels, increasing intestinal barrier dysfunction, thereby leading to more severe intestinal dysbiosis and increased bacterial migration. LGG-s attenuated liver injury in EAH mice but had no effect without pIgR. Our findings indicate that targeting intestinal pIgR may be a strategy to prevent AIH.

## Materials and methods

### Animal models

Six- to eight-week-old male B6J-*Pigr*^em1Cd12661/Gpt^ (*Pigr*^−/−^) mice and C57BL/6 J (WT) mice were acquired from GemPharmatech Co., Ltd. (Jiangsu, China). All mice were housed in a pathogen-free, temperature-controlled animal facility with a 12 h light/dark cycle. Randomization and blinding of researchers were implemented in animal experiments. Mice were euthanized to extract the liver antigen S100 [28]. Briefly, the liver was homogenized in pre-chilled phosphate buffered solution, centrifuged at 150 × *g* for 1 h, and then the supernatant was centrifuged at 100,000 × *g* for 1 h in an ultracentrifuge. The S100-containing supernatant was concentrated using an Amicon® Ultra-15 Centrifugal Filter Units (Millipore, Burlington, MA, USA) and then separated on an AKTA Pure (GE Healthcare, Chicago, IL, USA) 90 cm CL-6B Sepharose® column (Pharmacia & Upjohn Company LLC, Kalamazoo, MI, USA). The first non-toxic peak was obtained and the concentration was controlled at 0.5–2.0 g/L. Mice in the model group were intraperitoneally immunized with 0.5 mL liver S100 antigen emulsified with 0.5 mL complete Freund’s adjuvant (Sigma-Aldrich, St. Louis, MO, USA) on days 0 and 7, and mice in the control group were intraperitoneally injected with 1 mL sterile normal saline (NS). A group of mice were treated daily with non-absorbable antibiotics (Polymyxin B, 150 mg/kg and Neomycin, 200 mg/kg) from day 14, and control mice were gavaged with an equal volume of NS.

### Culture of LGG and preparation of LGG-s

*Lactobacillus rhamnosus* GG (BNCC 134266) was cultured in MRS broth according to the BeNa Culture Collection (BNCC) (BNCC, Henan, China) guidelines. When the bacterial density reached 10^9^ colony forming units/mL (CFU/mL), the LGG-s was obtained by filtration through a 0.22 μm filter. From day 7, each mouse was treated with LGG-s at a dose of 1 mL/day, and control mice were gavaged with an equal volume of NS.

### Histological analysis

Liver and small intestinal tissues were collected in 4% paraformaldehyde and then embedded in paraffin. Tissues were stained with hematoxylin and eosin (H&E) and analyzed by light microscopy (Olympus, Tokyo, Japan). Lymphocyte infiltration, inflammatory necrosis, and liver structure destruction were observed, and semi-quantitative analysis was performed using the Ishak scoring system [29].

### Biochemical analysis and enzyme-linked immunosorbent assay (ELISA)

The levels of serum transaminase were quantified using an automatic biochemical analyzer (AU5800, Beckman Coulter, Brea, CA, USA) in the Clinical Biochemical Laboratory of the First Affiliated Hospital of Wenzhou Medical University. Serum IgG levels were determined with an ELISA Kit (EK271, Multisciences (Lianke) Biotech, Co., Ltd., Shanghai, China) according to the manufacturer’s instructions. The concentrations of inflammatory cytokines IL-1β (EK201B/3, Multisciences), IL-6 (EK206/3, Multisciences), and TNF-α (EK282/4, Multisciences) in liver tissue lysates were measured. IgA levels were measured using an ELISA kit (A105446, Fusheng Industrial Co. Ltd., Shanghai, China).

### Quantitative real-time PCR (qRT-PCR)

Total RNA from liver and intestinal tissues was isolated with TRIzol reagent and was reverse transcribed using the 5X All-In-One RT MasterMix kit (G490, Applied Biological Materials (ABM), Vancouver, BC, Canada) to yield the cDNA. A quantitative polymerase chain reaction was performed in a 10 μL reaction mixture containing specific primers (GENEWIZ, Inc. Beijing, China) and BlasTaq 2X qPCR MasterMix (G891, ABM) on a real-time fluorescence quantitative PCR system (ABI 7500, Applied Biosystems, Waltham, MA, USA). The primers used in this study are listed in Supplementary Table 1. The relative mRNA expression levels of the target genes were evaluated by calculating the fold-change normalized to *Actb* for each sample using the 2^−ΔΔCt^ method. Specific bacteria quantitation was measured relative to the universal 16s gene.

### Western blotting

Liver and intestinal tissues were lysed in RIPA buffer containing protease inhibitors (Solarbio Science & Technology Co., Ltd., Beijing, China) and phosphatase inhibitors (Applygen Technologies Inc., Beijing, China). After centrifugation, the protein concentration of the lysate was determined with a BCA protein assay kit (P0012, Beyotime, Shanghai, China). An appropriate amount of protein was separated on SDS-PAGE and transferred onto polyvinylidene fluoride membranes (Millipore). The membranes were blocked after 1.5 h with 5% skim milk and incubated with antibodies against ZO-1 (Ab96587, Abcam, Cambridge, UK), Claudin-1 (Ab180158, Abcam), Occludin (Ab216327, Abcam), MEK1/2 (A4868, ABclonal, Wuhan, China), p-MEK1/2 (AF3385, Affinity Biosciences, Jiangsu, China), ERK1/2 (A4782, ABclonal), p-ERK1/2 (AP0234, ABclonal), pIgR (A6130, ABclonal), or GAPDH (A6130, Proteintech, Wuhan, China) overnight at 4 °C. Subsequently, the membranes were incubated with horseradish peroxidase-conjugated second antibodies against mouse or rabbit IgG (LF102 and LF101, respectively, Epizyme, Inc., Shanghai, China) for 1 h at 25 °C. Protein bands were visualized using a Bio-Rad immunoblot analysis detection system (Bio-Rad Laboratories, Hercules, CA, USA). Western blot band quantification was performed using ImageJ (Bethesda, MD, USA) software and normalized to GAPDH.

### Immunofluorescence (IF)

The liver and intestinal tissues or cells were fixed with 4% paraformaldehyde. After being blocked with 5% bovine serum albumin (BSA), the sections were incubated with antibodies against pIgR (A6130, ABclonal), F4/80 (A1256, ABclonal), Claudin-1 (Ab180158, Abcam), Occludin (Ab216327, Abcam), ZO-1 (Ab96587, Abcam), or CD20 (GB11540, Servicebio, Wuhan, China) overnight at 4 °C. Next, the sections were incubated with secondary antibodies in the dark for approximately 1 h at 25 °C and stained with 4′, 6-diamidino-2-phenylindole (Solarbio). Fluorescence images were obtained using a fluorescence microscope ECLIPSE C1. (Nikon, Tokyo, Japan).

### Immunohistochemistry

Immunohistochemistry (IHC) was performed using paraffin-embedded mouse intestinal tissues. The slides were blocked with BSA (5%) and incubated with anti-Ly6G (GB11229, Servicebio) overnight at 4 °C. Subsequently, the sections were incubated with biotinylated secondary antibodies, stained with diaminobenzidine, and counterstained with hematoxylin. Images were obtained using an optical microscope (Olympus).

### Short-chain fatty acid quantification

The LGG-s was transferred to a 2 mL centrifuge tube to which 50 μL of 15% phosphoric acid, 10 μL of 75 μg/mL internal standard (isocaproic acid) solution, and 140 μL of ether were added, vortexed for 1 min, and centrifuged at 12,000 rpm at 4 °C for 10 min. The supernatant was collected and used for chromatography-mass spectrometry (GC-MS) analysis (Trace 1310/ISQ LT, Thermo Fisher Scientific, Waltham, MA, USA). The SCFA standards were purchased from Sigma-Aldrich.

### Intestinal microbiota analysis

Fresh feces from individual mice were collected and stored at −80 °C. The fecal bacterial genomic DNA was isolated using the E.Z.N.A.® Stool DNA Kit (Omega Bio-Tek, Norcross, GA, USA). The V1–V9 region of the bacterial 16 S ribosomal RNA gene was amplified by PCR using the primers 27 F 5′-AGRGTTYGATYMTGGCTCAG-3′ and 1492 R 5′-RGYTACCTTGTTACGACTT-3′. Amplicons were extracted on 2% agarose gels and purified using an AxyPrep DNA Gel Extraction Kit (Axygen Biosciences Inc., Union City, CA, USA).

SMRTbell libraries were prepared from the amplified DNA by blunt ligation according to the manufacturer’s instructions (Pacific Biosciences, Menlo Park, CA, USA). Amplicon sequencing was performed by Shanghai Biozeron Biotechnology Co., Ltd. (Shanghai, China). PacBio raw reads were processed using SMRT Link Analysis software (version 9.0) to obtain demultiplexed circular consensus sequence reads. Operational Taxonomic Units (OTUs) were clustered with a 98.65% similarity cut-off using UPARSE (version 7.1), and chimeric sequences were identified and removed using UCHIME.

Subsequently, a representative sequence of each OTU was subjected to a taxonomy-based analysis using the RDP database. Beta diversity was analyzed using the QIIME software. The relative abundance of bacteria was expressed as a percentage value. Linear discriminant analysis effect size (LEfSe) analysis was performed to identify biomarkers for high-dimensional colonic bacteria. The Kruskal–Wallis sum-rank test was used to examine the changes and dissimilarities among classes.

### Endotoxin detection in the liver

Liver samples were assayed for lipopolysaccharide (LPS) levels using a chromogenic endotoxin quant kit (A39552, Thermo Fisher Scientific) according to the manufacturer’s instructions.

### Cell cultures

The human colorectal adenocarcinoma Caco-2 cells (BNCC 350769) were maintained in Dulbecco’s modified Eagle’s medium (DMEM; Gibco, Waltham, MA, USA) containing 10% fetal bovine serum and 1% penicillin-streptomycin solution (Solarbio) at 37 °C in a 5% CO_2_ humidified incubator. The cell lines were authenticated by STR profiling and tested for mycoplasma contamination. In the LPS group, cells were treated with LPS (1 μg/mL, Sigma-Aldrich) for 24 h. In the LPS + LGG-s + U0126 (MEK-inhibitor, used at 10 μM, GlpBio, Montclair, CA, USA) group, the cells were pretreated with LGG-s at three concentration gradients (1:100, 1:50, and 1:25) for 3 h, then treated with LPS and U0126 for 24 h. For *Pigr* knockdown, *Pigr*-specific knockdown siRNA and non-targeted control siRNA were transfected into cells by Lipofectamine 2000 Reagent (Thermo Fisher Scientific) following the standard procedure. The cells were incubated in OPTI medium (Gibco) for 6 h and then in a DMEM complete medium for 48 h. *Pigr*-specific siRNA (5′-GCAGAACGGUGACCAUCAATT-3′, 5′-UUGAUGGUCACCGUUCUGCTT-3′) and non-targeted control siRNA (5′-UUCUCCGAACGUGUCACGUTT-3′, 5′-ACGUGACACGUUCGGAGAATT-3′) were constructed by GenePharma (Shanghai, China).

### Molecular docking

AutoDockTools (version 1.5.7) were used for molecular docking to discover the interactions between the active ingredients and target proteins. The 3D structure of the acetic acid was downloaded from the PubChem database (https://pubchem.ncbi.nlm.nih.gov/). A 3D model of the receptor pIgR (ID: 4NOB) was acquired from the Protein Database Bank (https://www.resb.org/). The active ingredient and target protein were pre-treated with AutoDockTools. Finally, AutoDockTools was used for docking and determination of the optimal conformation. A total of 50 conformations were generated for each ligand-protein docking study, wherein, with lower binding energy score, stronger binding capacity between the protein and the molecule was observed. The best conformer with the minimum energy was visualized using PyMOL.

### Statistical analysis

Data are presented as the mean ± standard error of the mean from at least three independent experiments. Statistical significance of differences was assayed by one-way ANOVA in multiple groups, and *t*-tests for two groups using GraphPad Prism 9.0 (GraphPad Software Inc., San Diego, CA, USA). *P* > 0.05 was considered significant.

## Results

### PIgR level is strongly associated with disease development in EAH

**Fig. 1:**
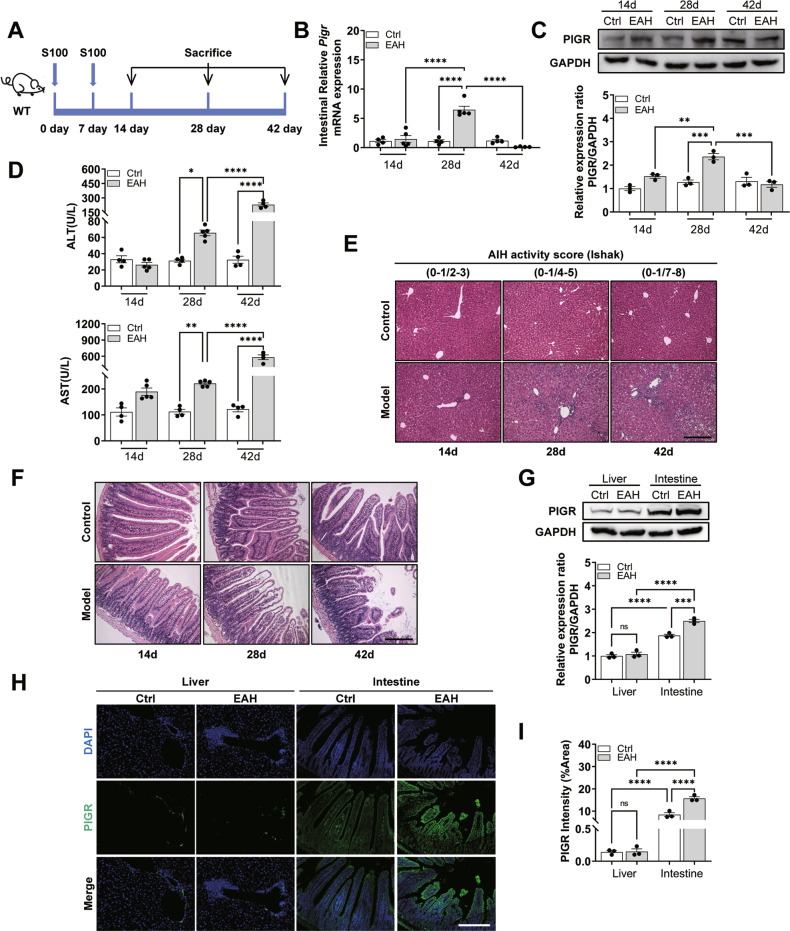
Polymeric immunoglobulin receptor (pIgR) level is strongly associated with disease development in experimental autoimmune hepatitis (EAH). **A** Wild-type (WT) mice were intraperitoneally injected with S100 on days 0 and 7 and euthanized on days 14, 28, and 42. **B** *Pigr* gene expression at different time points. **C** Western blotting analysis for intestinal pIgR normalized by GAPDH. **D** Changes in serum alanine aminotransferase (ALT) and aspartate aminotransferase (AST) in mice with time. **E**, **F** Hematoxylin and eosin (H&E) staining of the liver and intestine in mice. Scale bar = 200 μm. **G** Western blotting analysis for intestinal and liver pIgR normalized by GAPDH. **H**, **I** Immunofluorescence (IF) staining and quantification of pIgR. Scale bar = 200 μm. Data are presented as mean ± standard error of the mean (SEM). Statistical analysis was performed using one-way ANOVA with Tukey multiple comparisons.

We detected pIgR expression in the intestinal tract of S100-induced EAH mice at different time points (Fig. 1A). Intestinal *Pigr* mRNA expression levels gradually increased on days 14 and 28 but decreased on day 42 (Fig. 1B). The intestinal pIgR protein expression levels at different time points were consistent with the changes observed in its mRNA levels (Fig. 1C). Furthermore, we analyzed the correlation between the expression of intestinal pIgR and the degree of intestinal and liver injury in mice. Serum alanine aminotransferase (ALT) and aspartate aminotransferase (AST) levels in mice increased gradually with time (Fig. 1D). The infiltration of inflammatory cells around the central vein of the hepatic lobule and the Ishak index both also gradually increased (Fig. 1E). There were similar changes in the gut, where the damage was gradually aggravated and manifested as atrophy and sparse villi (Fig. 1F). Simultaneously, the pIgR protein was mainly enriched in intestinal epithelial cells, and in the model group (28 days), the intestinal pIgR protein increased notably, whereas the liver pIgR protein had no notable change (Fig. 1G–I). The level of intestinal pIgR increased in the early stage of S100-induced liver injury and decreased with the progression of the liver injury. We hypothesized that the upregulation of intestinal pIgR is an adaptive response to S100 and that prolonged injury disrupts this adaptive mechanism.

### Deletion of *Pigr* exacerbates S100-induced liver injury

**Fig. 2:**
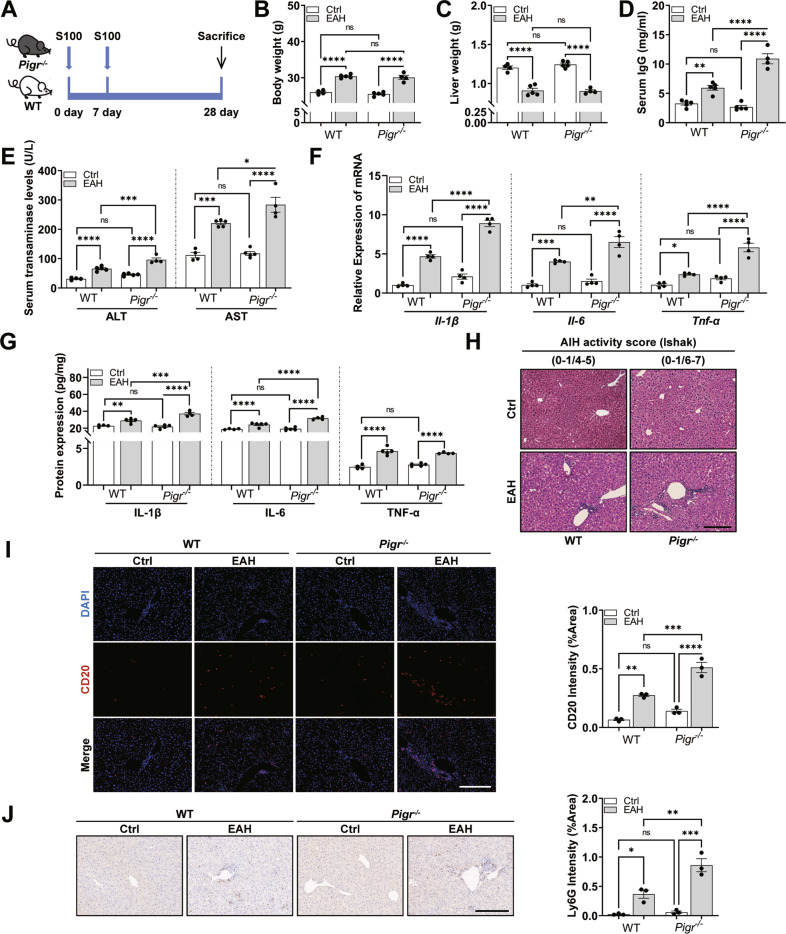
*Pigr*^−/−^ mice have exacerbated S100-induced liver injury. **A** The modeling process of EAH. **B**, **C** Body weight and liver weight. **D** Serum immunoglobulin G (IgG) level. **E** Serum ALT and AST levels. **F**, **G** Hepatic gene and protein expression of pro-inflammatory cytokines IL-1β, TNF-α, and IL-6. **H** H&E staining of the liver. Scale bar = 200 μm. **I** CD20 staining of the liver. Scale bar = 200 μm. **J** Ly6G staining of the liver. Scale bar = 200 μm. Data are presented as mean ± SEM. Statistical analysis was performed using one-way ANOVA with Tukey multiple comparisons.

Observations mentioned above confirmed that *Pigr* is strongly linked to the progression of EAH, and we speculated that depletion of *Pigr* may disrupt intestinal homeostasis and aggravate liver injury. Consequently, we used *Pigr*^−/−^ mice to investigate the changes in liver and gut pathophysiology after S100 intervention on day 28 to further probe the role and mechanism of *Pigr* in EAH (Fig. 2A).

Mice in the four groups (wild-type (WT)-Ctrl, WT-EAH, *Pigr*^−/−^-Ctrl, *Pigr*^−/−^-EAH) were observed and compared daily. Mice in the WT and *Pigr*^−/−^ control groups were in good mental condition, active, exhibited shiny hair, and consumed regular food and water. Intraperitoneal injection of S100 caused the mice to develop ascites and increased their body weights, while their activity level and the weight of the liver decreased. However, this change was independent of whether *Pigr* was knocked out or not (Fig. 2B, C).

Deletion of the *Pigr* exacerbated liver inflammation in mice with EAH. The levels of ALT, AST, and IgG were remarkably increased in the *Pigr*^−/−^ group (Fig. 2D, E). Additionally, pro-inflammatory factors IL-6, IL-1β, and TNF-α were increased in the liver of *Pigr*^−/−^ mice (Fig. 2F, G). It is precisely because of the intense infiltration and activation of lymphocytes, neutrophils and macrophages that lead to the increase of pro-inflammatory factors (Fig. 2H). As shown via immunofluorescence staining with CD20, immunohistochemical staining with Ly6G, and immunofluorescence staining with F4/80, *Pigr*^−/−^ mice had increased infiltrations of B lymphocytes (Fig. 2I), neutrophils (Fig. 2J), and macrophages (Fig. S1), respectively. These results demonstrated that *Pigr*^−/−^ mice exhibited enhanced liver inflammation.

### *Pigr*^−/−^ mice develop gut dysbiosis

Studies have shown that AIH is associated with an imbalance in the gut flora [3]. Next, to determine whether gut *Pigr* deficiency affects gut microbiota homeostasis, we analyzed the fecal microbiota using 16 S rDNA full-length amplicon sequencing. The raw data have been uploaded to the NCBI database under project number: PRJNA908898.

**Fig. 3:**
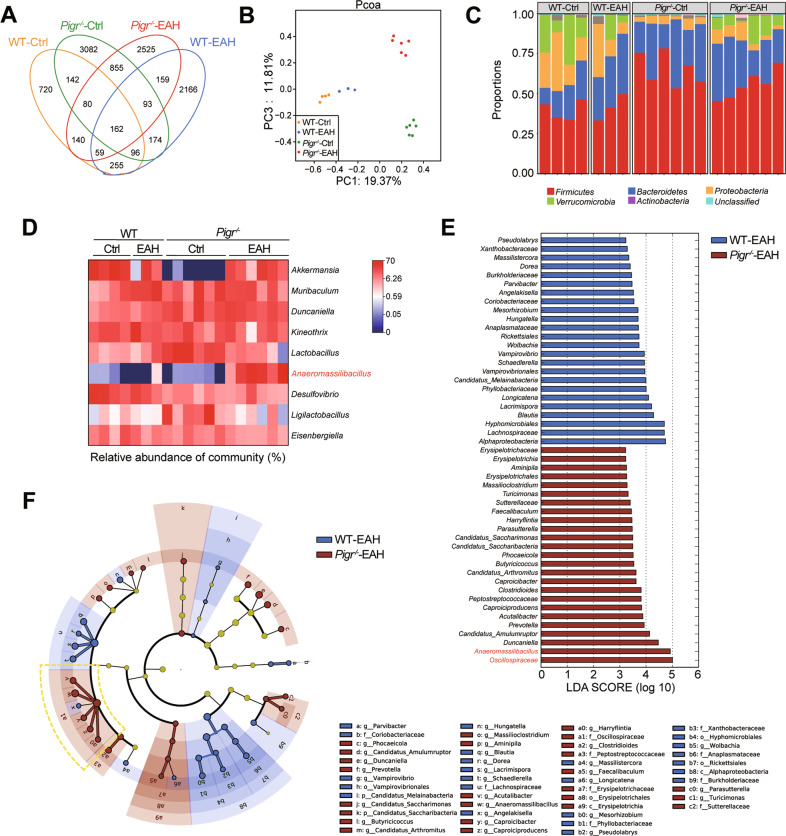
*Pigr*^−/−^ mice develop gut dysbiosis. **A** Venn diagram. **B** Principal component analysis of unweighted UniFrac analysis. **C** Relative abundance of bacteria at the phylum level. **D** Heatmap of bacteria at the genus level. **E** Linear discriminant analysis (LDA) score. **F** Cladogram plot of LEfSe analysis. The yellow frame indicates the 5 significantly differential genus in *Oscillospiraceae* family.

On comparing the OTUs among the four groups, 720 OTUs in the control group, 2166 in the EAH group, 3082 in the *Pigr*^−/−^ group, and 2525 in the *Pigr*^−/−^-EAH group were obtained. The four groups shared 162 OTUs in common (Fig. 3A). Principal coordinate analysis on the basis of unweighted UniFrac distances showed distinct structures among the four groups (Fig. 3B). The above results showed that the four groups had slight intragroup differences, marked intergroup differences, and high data quality. Phylum-level analysis showed that the intestinal flora of all groups was dominated by *Bacteroidetes*, *Firmicutes*, and *Proteobacteria*. *Bacteroidetes* were more abundant in the model group than the control group, resulting in a lower *Firmicutes*/*Bacteroidetes* ratio. *Pigr*^−/−^ remarkably elevated the abundance of *Firmicutes* and *Bacteroidetes* (Fig. 3C). From the genus-level analysis it was observed that *Akkermansia* decreased markedly in *Pigr*^−/−^-WT mice, whereas *Lactobacillus* increased, and *Anaeromassilibacillus* was markedly increased in *Pigr*^−/−^-EAH mice (Fig. 3D). Furthermore, the different abundances of species between WT-EAH and *Pigr*^−/−^-EAH mice were compared using LEfSe. We found a notable increase in the *Oscillospiraceae* family, particularly the *Anaeromassilibacillus* genus in *Pigr*^−/−^-EAH mice (Fig. 3E, F). These results demonstrated that *Pigr* knockout notably altered the composition of gut microbiota in mice.

### *Pigr* deficiency induces intestinal inflammation and barrier dysfunction in EAH mice

**Fig. 4:**
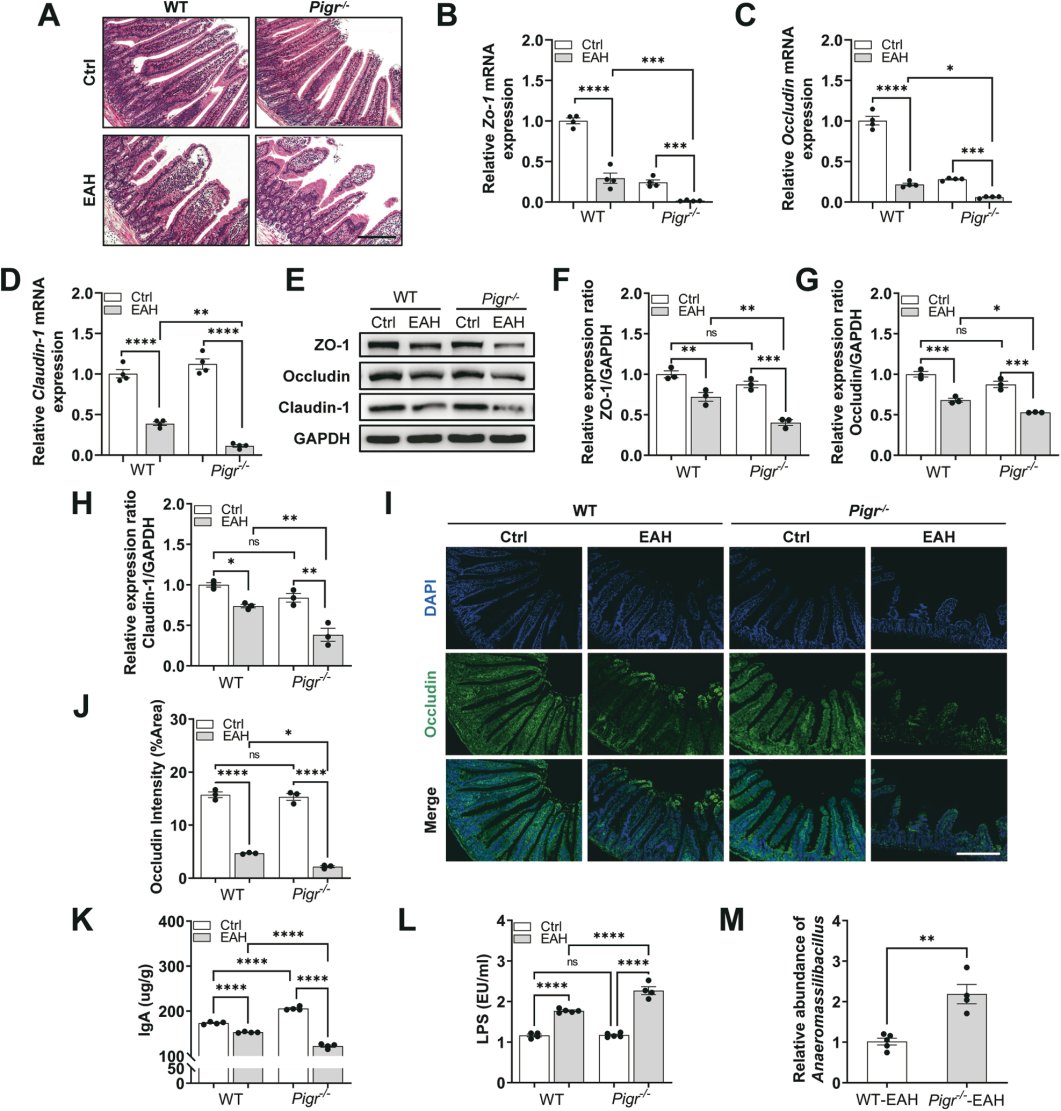
Effect of *Pigr* on intestinal inflammation and barrier integrity. **A** H&E staining of the intestine in mice. Scale bar = 200 μm. **B**–**D** Gene expression of *Zo-1*, *Occludin*, and *Claudin-1*. **E**–**H** Western blotting analysis for ZO-1, Occludin, and Claudin-1 normalized by GAPDH. **I**, **J** IF staining and quantification of Occludin. Scale bar = 200 μm. **K** Immunoglobulin A (IgA) levels. **L** Liver lipopolysaccharides (LPSs) level. **M** The abundance of the *Anaeromassilibacillus* genus between WT-EAH and *Pigr*^*−/−*^-EAH was validated by quantitative PCR. Data are presented as mean ± SEM. Statistical analysis was performed using one-way ANOVA with Tukey multiple comparisons.

Increased liver inflammation and intestinal microbiota dysbiosis were observed in *Pigr*^−/−^ mice. The bacterial translocation is highly impacted by intestinal inflammation and barrier dysfunction. Intestinal H&E staining showed that intestinal inflammation in the WT-EAH group was remarkably enhanced, whereas the disruption worsened notably in *Pigr*^−/−^-EAH mice (Fig. 4A). Immunofluorescence staining, western blotting, and PCR showed that the expression of the intestinal TJ proteins (ZO-1, Occludin, and Claudin-1) in the *Pigr*^−/−^-EAH group was markedly decreased compared to those in the other groups (Fig. 4B–J). IgA was decreased in the intestine of WT-EAH mice, and the decrease was more obvious in *Pigr*^−/−^-EAH mice (Fig. 4K). These results suggest that *Pigr* deficiency aggravates intestinal inflammation and barrier dysfunction in EAH mice.

### Increased intestinal permeability in *Pigr*^−/−^ mice

Increased intestinal inflammation and disruption of the barrier function results in increased intestinal permeability, which facilitates bacterial translocation. We examined *Anaeromassilibacillus* and LPSs in EAH mice livers to determine the role of *Pigr* in the translocation of bacteria or bacterial products to the liver. We confirmed a high abundance of *Anaeromassilibacillus* and LPSs in *Pigr*^−/−^-EAH mice (Fig. 4L, M).

### Antibiotic intestinal decontamination reduces liver injury *Pigr*^−/−^-EAH mice

To further analyze the role of intestinal bacteria in *Pigr*^−/−^ mice, the mice were given non-absorbable antibiotics (ABx, Polymyxin B, and Neomycin) for 14 days. As shown in Fig. S2, ABx treatment reduced liver LPSs (Fig. S2A), serum ALT/AST (Fig. S2B), and liver inflammation (Fig. S2C) in *Pigr*^−/−^-EAH mice. These results support the concept that gut dysbiosis is critical in the *Pigr*^−/−^-EAH mice.

### *Pigr* is needed for the therapeutic effect of LGG-s in EAH mice

LGG is the best-characterized probiotic strain that plays a key role in preventing and treating liver diseases via intestinal microbiota modification. Probiotics affect intestinal microecology and intestinal and systemic immunity via their metabolites. SCFAs including acetate, propionate, and butyrate are the most significant metabolites [30]. Therefore, we used GC-MS analysis to detect LGG-s and found that the supernatant contained a large amount of SCFAs, of which acetic acid was the most abundant (Fig. S3A).

Next, we performed molecular docking to determine whether acetic acid could directly activate pIgR and their interaction sites. To predict the binding sites of acetic acid on pIgR, we performed binding energy calculations for the 50 docking sites. The two key residues with the lowest energy values in the extracellular binding domain of pIgR were identified as ARG52 and ASN71 (Fig. S3B).

**Fig. 5:**
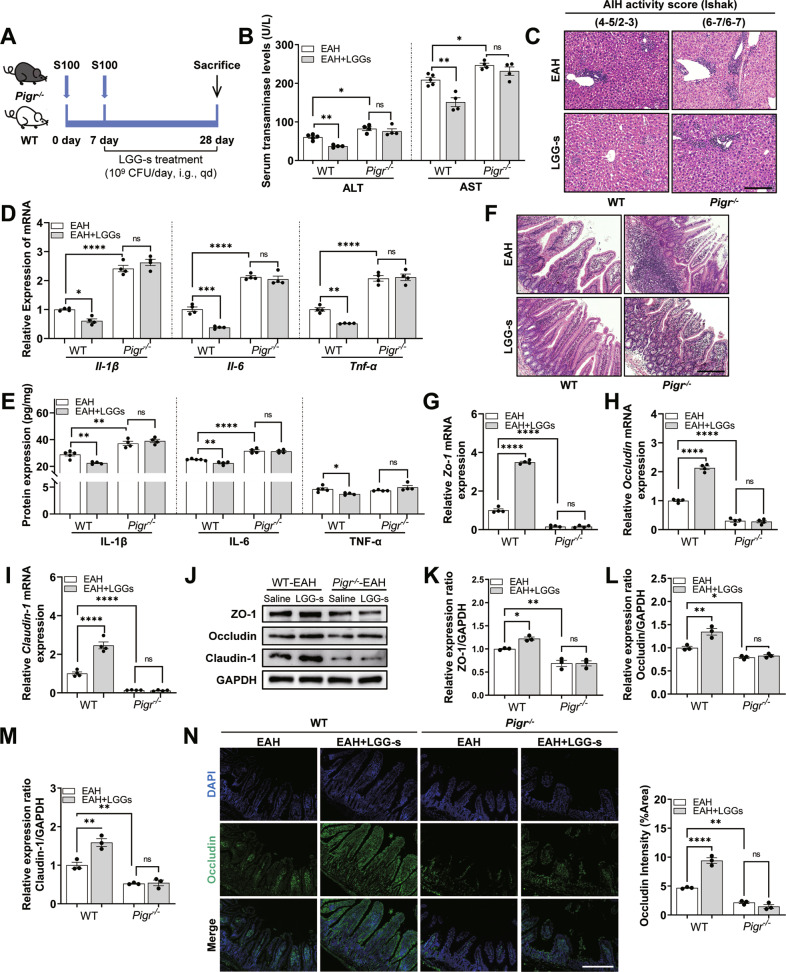
Effects of *Pigr* on *Lactobacillus rhamnosus GG* culture supernatant (LGG-s) treatment in EAH. **A** Schematic of the LGG-s treatment regimen for WT and *Pigr*^−/−^-EAH mice. Mice were intraperitoneally injected with S100 on days 0 and 7, followed by i.g. with LGG-s (10^9^ CFU/ml) or normal saline every day until day 28. **B** Serum ALT and AST levels. **C**, **F** H&E staining of the liver and intestine in mice. Scale bar = 200 μm. **D**, **E** Hepatic gene and protein expression of pro-inflammatory cytokines IL-1β, TNF-α, and IL-6. **G**–**I** Gene expression of *Zo-1*, *Occludin*, and *Claudin-1*. **J**–**M** Western blotting analysis for ZO-1, Occludin, and Claudin-1 normalized by GAPDH. **N** IF staining and quantification of Occludin. Scale bar = 200 μm. Data are presented as mean ± SEM. Statistical analysis was performed using one-way ANOVA with Tukey multiple comparisons.

We investigated whether pIgR is needed for the therapeutic effects of LGG-s. After two intraperitoneal injections of S100, mice were given daily gavage starting from day 7 with 1 ml of LGG-s (10^9^ CFU/ml) or normal saline until day 28 (Fig. 5A). LGG-s treatment resulted in modest improvements in serum ALT and AST levels in WT-EAH mice, but not in *Pigr*^−/−^-EAH mice (Fig. 5B). LGG-s administration markedly reduced liver inflammation in WT-EAH mice, whereas this therapeutic effect was attenuated in *Pigr*^−/−^-EAH mice (Fig. 5C–E). In addition, LGG-s ameliorated intestinal inflammation (Fig. 5F) and upregulated the expression of intestinal TJ mRNA and proteins (Fig. 5G–N) in WT-EAH mice but not in *Pigr*^−/−^-EAH mice. These results demonstrated that *Pigr* is needed for the therapeutic effects of LGG-s in the treatment of EAH.

### *Pigr* deletion reduces IgA transport by inhibiting MEK/ERK signaling in EAH

**Fig. 6:**
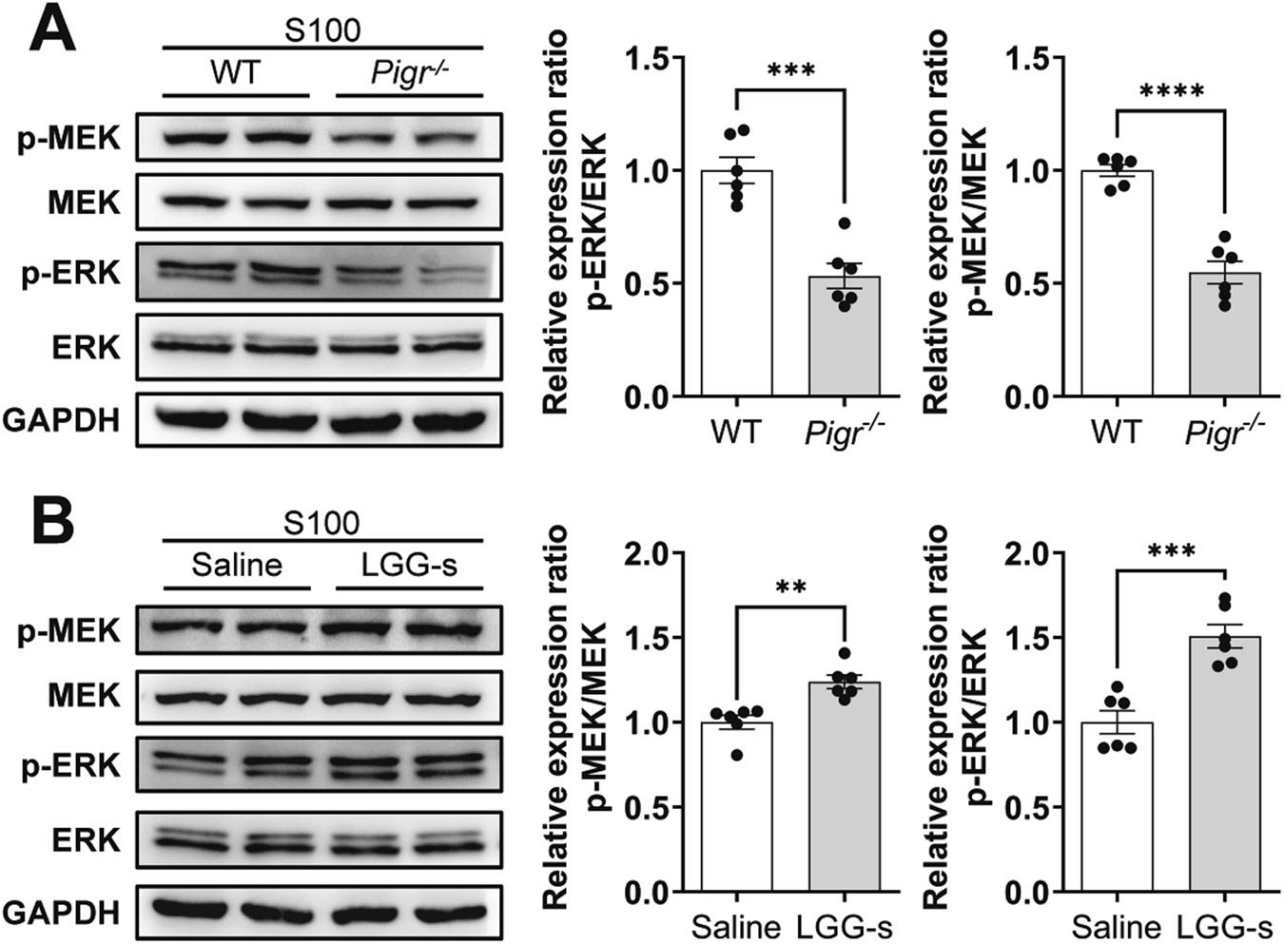
Effects of *Pigr* deletion and LGG-s on intestinal MEK/ERK phosphorylation. Western blotting analysis for the p-MEK and p-ERK normalized by MEK and ERK. **A** *Pigr*^−/−^ inhibits intestinal MEK/ERK phosphorylation and (**B**) LGG-s promotes intestinal MEK/ERK phosphorylation. Data are presented as mean ± SEM. Statistical analysis was performed using Student’s *t*-test.

Studies have shown that the MEK/ERK pathway is involved in the transcytosis of IgA-pIgR [20]. Nevertheless, whether MEK/ERK is a critical pathway that mediates pIgR to regulate EAH inflammation remains unclear. First, we analyzed the impact of *Pigr*^−/−^ on MEK/ERK activation. We found that the phosphorylation level of MEK/ERK was decreased in the *Pigr*^−/−^-EAH mice (Fig. 6A) and LGG-s supplementation promoted MEK/ERK phosphorylation (Fig. 6B). Initially, it was suggested that the MEK/ERK pathway has a critical role in the therapeutic effects of LGG-s in AIH.

Our data showed that LGG-s reduced liver damage in EAH mice by improving the intestinal barrier, in which pIgR plays a key role. Moreover, preliminary observations have shown that it can be mediated via the MEK/ERK pathway. We used an in vitro model (LPS-treated Caco-2 epithelial cells) to further study the effects and mechanisms of LGG-s on intestinal barrier function. In the LPS group, the expression of the vital protein pIgR was markedly increased, while intestinal TJ proteins were substantially reduced. Treatment with LGG-s alleviated this change, with the most notable alteration in the ratio of LGG-s observed at 1:50 (Fig. S4A, B).

**Fig. 7:**
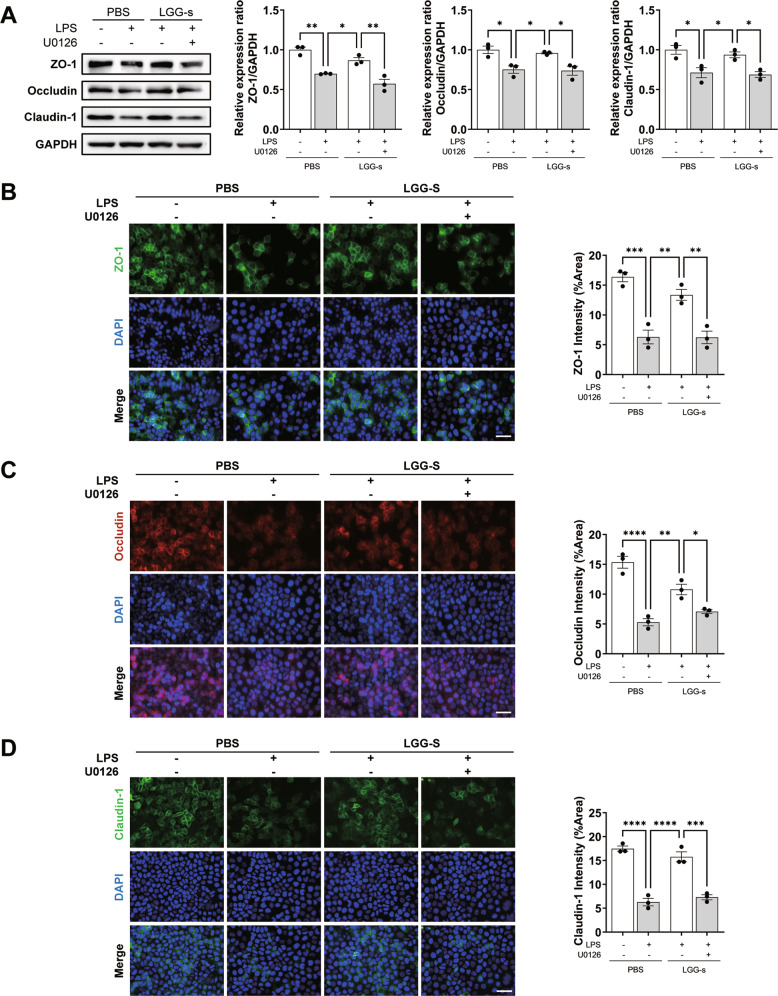
LGG-s lost the improvement of barrier function after MEK inhibition. **A** Western blotting analysis for ZO-1, Occludin, and Claudin-1 normalized by GAPDH. **B**–**D** IF staining and quantification of ZO-1, Occludin, and Claudin-1. Scale bar = 50 μm. Data are presented as mean ± SEM. Statistical analysis was performed using one-way ANOVA with Tukey multiple comparisons.

Next, we knocked down *Pigr* in Caco-2 cells using *Pigr*-specific siRNA and found that LGG-s did not reverse TJ protein damage or restore MEK/ERK phosphorylation levels in the *Pigr*^−/−^ group treated with LPS (Fig. S4C, D). Furthermore, in vitro experiments demonstrated that the loss of *Pigr* abolished the protective function of LGG-s on the gut and that the MEK/ERK pathway was involved. Finally, we used the MEK inhibitor U0126 to further elucidate whether the therapeutic effect of LGG-s on the intestinal epithelial barrier is related to the MEK/ERK signaling pathway. As shown in Fig. 7A–D, the MEK inhibitor U0126 inhibited the LGG-s-induced increase in the expression of ZO-1, Occludin, and Claudin-1, thus confirming that the LGG-s-induced activation of the MEK/ERK signaling pathway is *Pigr*-dependent.

## Discussion

AIH is a chronic immune-mediated inflammatory liver disease of unknown etiology [1]. It has been found that changes in the intestinal microecology are associated with AIH [5, 6]. The purpose of this study was to explore how the gut microecology affects AIH. We found that loss of gut *Pigr* increased intestinal dysbiosis and altered gut integrity, leading to increased liver inflammation and damage in EAH mice.

IgA plays a key role in maintaining intestinal homeostasis [31]. The transport of IgA in the epithelial layer is mediated by pIgR [32]. Studies using *Pigr*^−/−^ mice have provided strong support for the theory that sIgA inhibits the colonization of *Salmonella typhimurium* or *Mycobacterium bovis* in the intestine [33–35]; however, the role of intestinal *Pigr* in AIH has not previously been elucidated. In this study, the upregulation in *Pigr* following a relatively short duration of S100 stimulation (14 and 28 days) may represent adaptive response. However, this adaptive response decreases with disease progression. Therefore, we speculated whether *Pigr* is an early and specific biomarker of AIH intestinal injury with an essential role in the occurrence and progression of AIH.

Host-microbe symbiosis is achieved by maintaining a balanced interaction between the host immunity and gut-associated bacteria [36]. When the intestinal microecology is disrupted, the intestinal mucosa responds to adaptive mechanisms, such as the upregulation of antimicrobial substances to restore homeostasis. We hypothesized that epithelial *Pigr* represents this adaptive mechanism. S100 stimulation for 28 days slightly altered the homeostasis of intestinal microbiota in WT mice. We observed that the *Pigr* deficiency leads to complex dysbiosis. Elevated *Bacteroides* in the EAH group resulted in lower *Firmicutes*/*Bacteroidetes* ratios, which are widely considered dysbiosis indicators [37]. *Pigr*^−/−^ markedly increased levels of *Firmicutes* and *Bacteroidetes*, causing another complex disorder in the gut microbiota, a shift that requires further examination. Notably, the abundance of *Akkermansia* in *Pigr*^−/−^-WT mice was almost zero. Recently, *Akkermansia* has been a prevalent bacterium in intestinal research and is considered a beneficial microbe. The reduction observed in our study suggests that simple *Pigr* knockout affects gut microbiota. Furthermore, we identified a distinct microbiota profile of *Pigr*^−/−^-EAH mice, with a significant increased aboundance in the *Oscillospiraceae* family, particularly the *Anaeromassilibacillus* genus. Although the specific bacteria that exert a notable influence on EAH remain elusive, the absence of *Pigr* can certainly disrupt the microbiome. The intestinal flora is a complex system, and it may not be a single bacterium that plays an absolute role; however, the increase in populations of some bacteria might have a specific predictive effect.

The disturbance of the gut microbiota caused the earliest response pIgR molecules to respond, and the massive depletion led to the impairment of the pIgR function in transporting IgA, thereby aggravating the disturbance of the gut microbiota. Then, it affected other intestinal barrier functions such as ZO-1, Occludin, and Claudin-1, which are vital for maintaining the integrity of the intestinal barrier [38, 39]. The decrease of these intestinal TJ proteins in *Pigr*^−/−^-EAH mice disrupted intestinal barrier function, resulting in “bacterial translocation,” which increased the concentration of LPSs and *Anaeromassilibacillus* in the liver. Moreover, intestinal inflammation was markedly increased in *Pigr*^−/−^-EAH mice. In summary, these multiple mechanisms resulted in intestinal barrier dysfunction in *Pigr*^−/−^-EAH mice and aggravated liver damage via the gut-liver axis. The pathogenic role of gut dysbiosis in *Pigr*^−/−^ mice in EAH was further demonstrated following the administration of non-absorbable gut antibiotics.

Studies have shown that probiotics are beneficial for the prevention and treatment of EAH in mice [40, 41]. SCFAs are the most important markers of gut microbiota metabolism [42]. Probiotics can increase the production of intestinal SCFAs, which upregulate TJ proteins, thereby enhancing the integrity of the epithelial barrier [43]. Researchers have shown that LGG can improve several clinically relevant models of liver injury in mice, such as those with non-alcoholic fatty liver disease and alcoholic fatty liver disease [44, 45]. However, it is difficult for probiotics to colonize damaged intestinal mucosal surfaces, which may cause adverse effects, such as bacteremia, in infants or immunocompromised patients [46, 47]. Previous studies have shown that LGG-s can protect against gut-derived liver disorders [24, 25]. In this study, we found that SCFAs were enriched in LGG-s, the most abundant of which, acetic acid, could directly bind to pIgR. Moreover, another study has shown that SCFAs can promote the production of IgA by activating the mTOR signaling and glycolysis pathways in B cells [26]. Acetic acid not only increases the production of IgA, but also alters the capacity of the IgA pool to bind to specific microorganisms [48]. Thus, LGG-s may promote the production and transport of IgA by directly binding to pIgR and increasing the activation of B cells, thereby enhancing the intestinal immune function. Furthermore, intestinal *Pigr* depletion abolished the protective effects of LGG-s against intestinal barrier disruption in EAH.

The MEK/ERK signaling pathway is vital for communicating intracellular and extracellular signals [49]. In addition, a previous study has shown that the MEK/ERK pathway is an important pathway for pIgR to transport IgA [20]. Our study found that the intestinal MEK/ERK phosphorylation level was markedly reduced in *Pigr*^−/−^-EAH mice and that LGG-s restored the intestinal MEK/ERK phosphorylation level in EAH. In the presence of MEK inhibition, LGG-s failed to correct the destruction of the intestinal barrier function. These results indicate that activation of the MEK/ERK signaling pathway is crucial for the protective effects of LGG-s in EAH and is achieved based on *Pigr*.

Our research results suggest that intestinal epithelial pIgR is closely associated with barrier disruption and subsequent liver injury in EAH mice. Activation of pIgR maintained intestinal bacterial homeostasis by regulating IgA transport via the MEK/ERK signaling pathway and upregulated TJ proteins to stabilize barrier function. These findings may broaden our understanding of the role of pIgR in AIH and supports pIgR as a possible novel molecular target for the treatment of AIH. However, there are still some limitations in our study, for example, we constructed *Pigr* full-gene knockout mice instead of intestinal epithelial-specific knockout mice, which may have some implications to our study. In the future, we will consider the use of intestinal epithelial-specific *Pigr-*knockout mice for further research.

## Supplementary information

fig.S1

fig.S2

fig.S3

fig.S4

Supplementary Figure Legends

Supplementary Table 1

aj-checklist

Original Data File-Western blot

## Data Availability

The data will be available upon reasonable request to the corresponding author.
