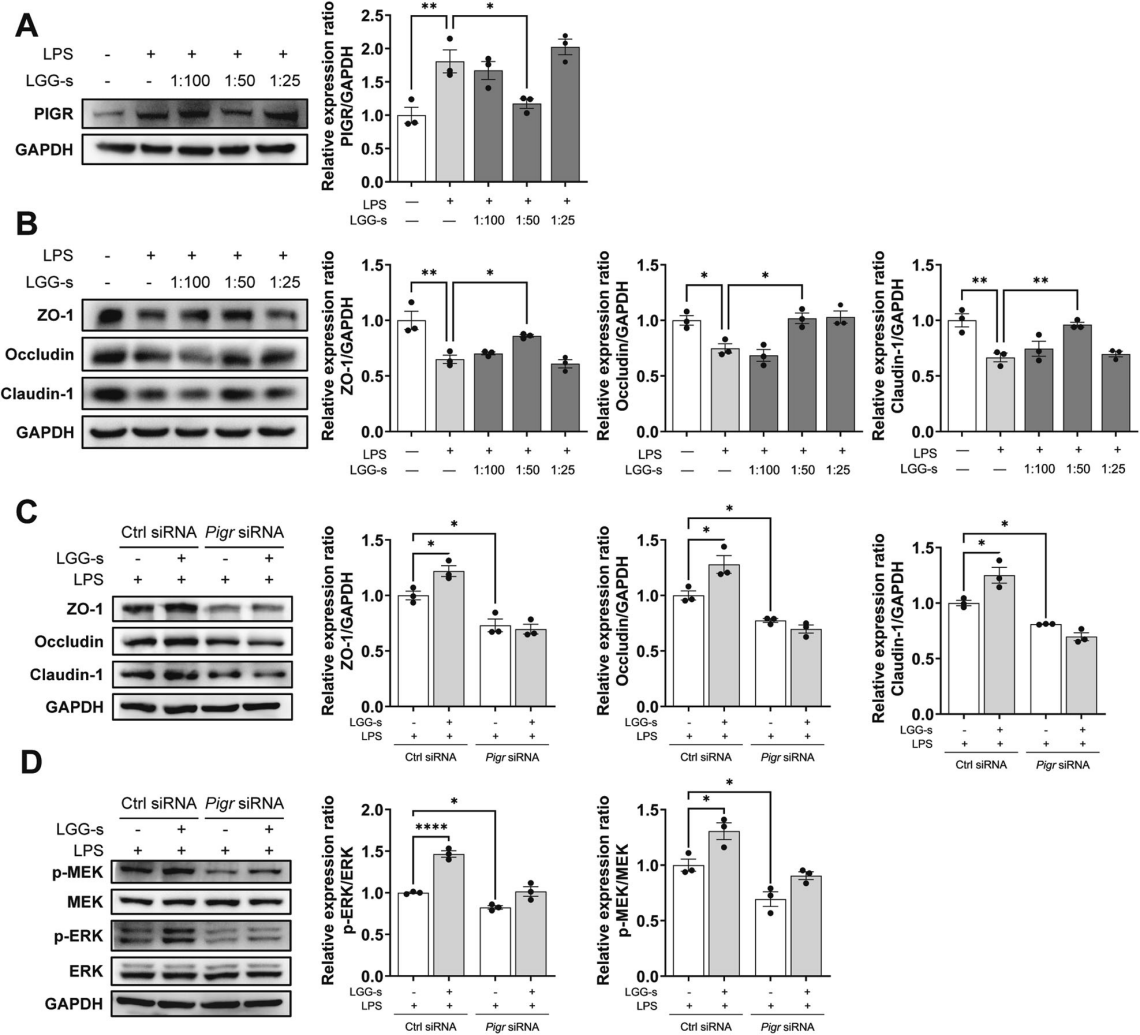# Correction: Polymeric immunoglobulin receptor deficiency exacerbates autoimmune hepatitis by inducing intestinal dysbiosis and barrier dysfunction

**DOI:** 10.1038/s41419-023-05816-x

**Published:** 2023-05-02

**Authors:** Hongwei Lin, Jing Lin, Tongtong Pan, Ting Li, Huimian Jiang, Yan Fang, Yuxin Wang, Faling Wu, Jia Huang, Huadong Zhang, Dazhi Chen, Yongping Chen

**Affiliations:** 1 Liver Disease Diagnosis and Treatment Center, The First Affiliated Hospital of Wenzhou Medical University, Hepatology Institute of Wenzhou Medical University, Wenzhou, 325000 Zhejiang China; 2 Zhejiang Provincial Key Laboratory for Accurate Diagnosis and Treatment of Chronic Liver Diseases, Wenzhou, 325000 Zhejiang China; 3 Hangzhou Medical College, Hangzhou, 310059 Zhejiang China

**Keywords:** Autoimmunity, Autoimmune diseases

Correction to: *Cell Death and Disease* 10.1038/s41419-023-05589-3, published online 28 January 2023

The original version of this article contained an error in the affiliation of the first corresponding author. The correct affiliation of the first corresponding author Yongping Chen should be 1, 2, not 1, 2, 3. (1 Liver Disease Diagnosis and Treatment Center, The First Affiliated Hospital of Wenzhou Medical University, Hepatology Institute of Wenzhou Medical University, Wenzhou, 325000 Zhejiang, China 2 Zhejiang Provincial Key Laboratory for Accurate Diagnosis and Treatment of Chronic Liver Diseases, Wenzhou, 325000 Zhejiang, China). In addition, the original version of this article contained an error in Figure 4J. The authors showed the representative images and statistical graph for immunofluorescence of the intestinal Occludin protein. There is no problem with the representative images and conclusions of immunofluorescence, but the author mistakenly placed Figure 5N instead of Figure 4J. The original version of this article also contained an error in Figure S4C and S4D. The authors knocked down Pigr in vitro to further investigate the function of pIgR. The groups should be LPS + ctrl siRNA, LPS + ctrl siRNA + LGG-s, LPS + Pigr siRNA, and LPS + Pigr siRNA+ LGG-s respectively. The author made a mistake in the position of LPS and LGG-s, which caused this error, but there is no error in the experimental conclusions and statistical graph. These mistakes were generated during submission. The authors sincerely apologize for these mistakes. The correct figures can be found below. The original article has been corrected.

Fig. 4
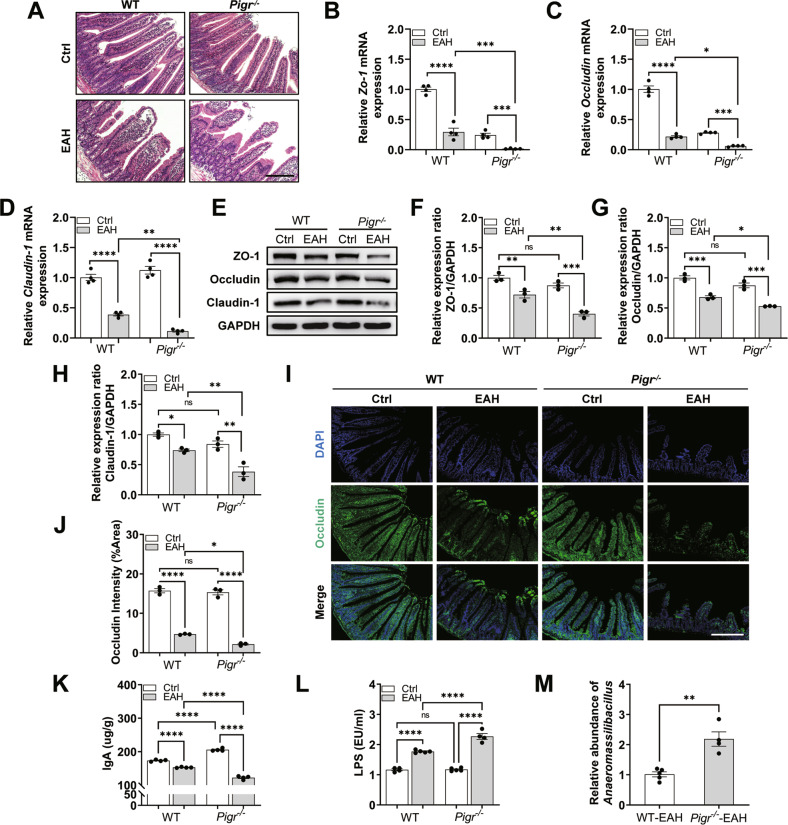


Fig. S4