# Supplementary figures and images for: Polymeric immunoglobulin receptor deficiency exacerbates autoimmune hepatitis by inducing intestinal dysbiosis and barrier dysfunction
Source: Cell Death Dis. 2023 Jan 28;14(1):68. doi: 10.1038/s41419-023-05589-3 (PMC9884241; doi:10.1038/s41419-023-05589-3)

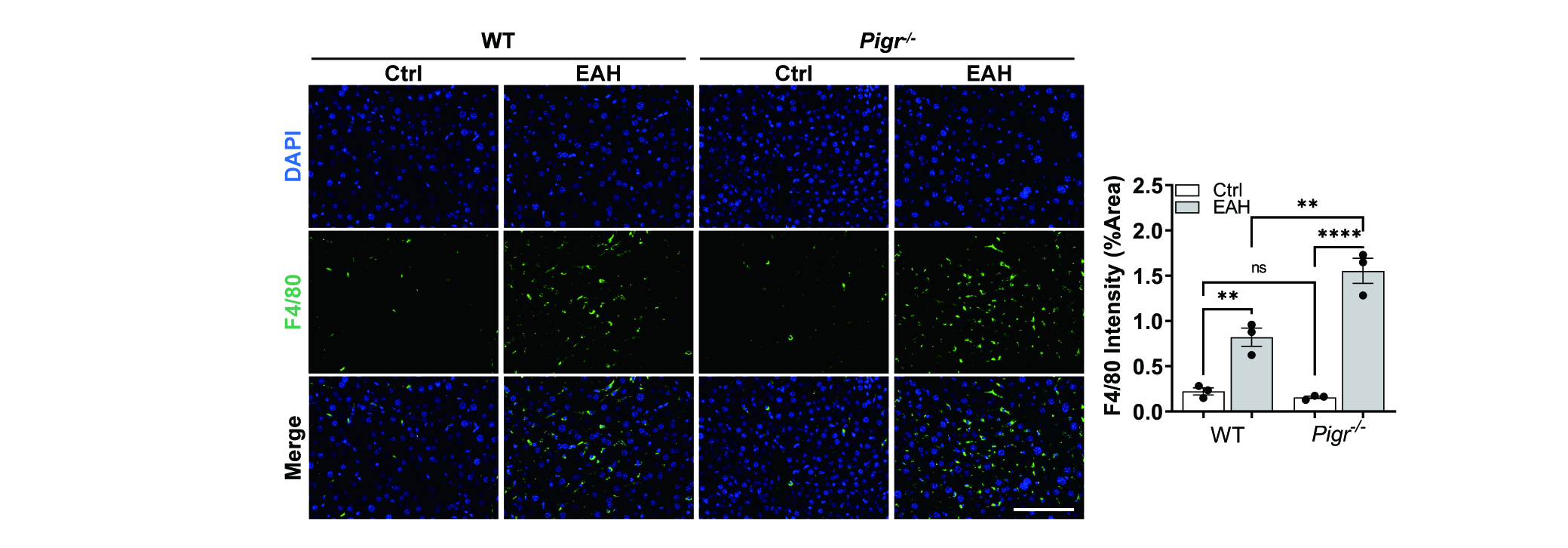

Supplement: Supplementary file 1 — fig.S1 [file 41419_2023_5589_MOESM1_ESM.tif]

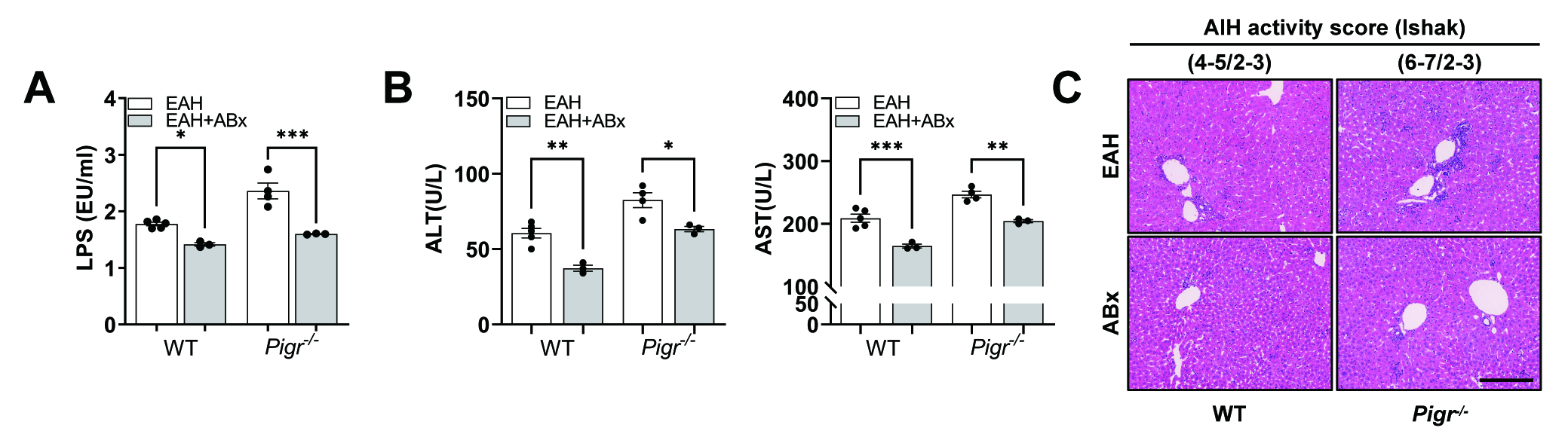

Supplement: Supplementary file 2 — fig.S2 [file 41419_2023_5589_MOESM2_ESM.tif]

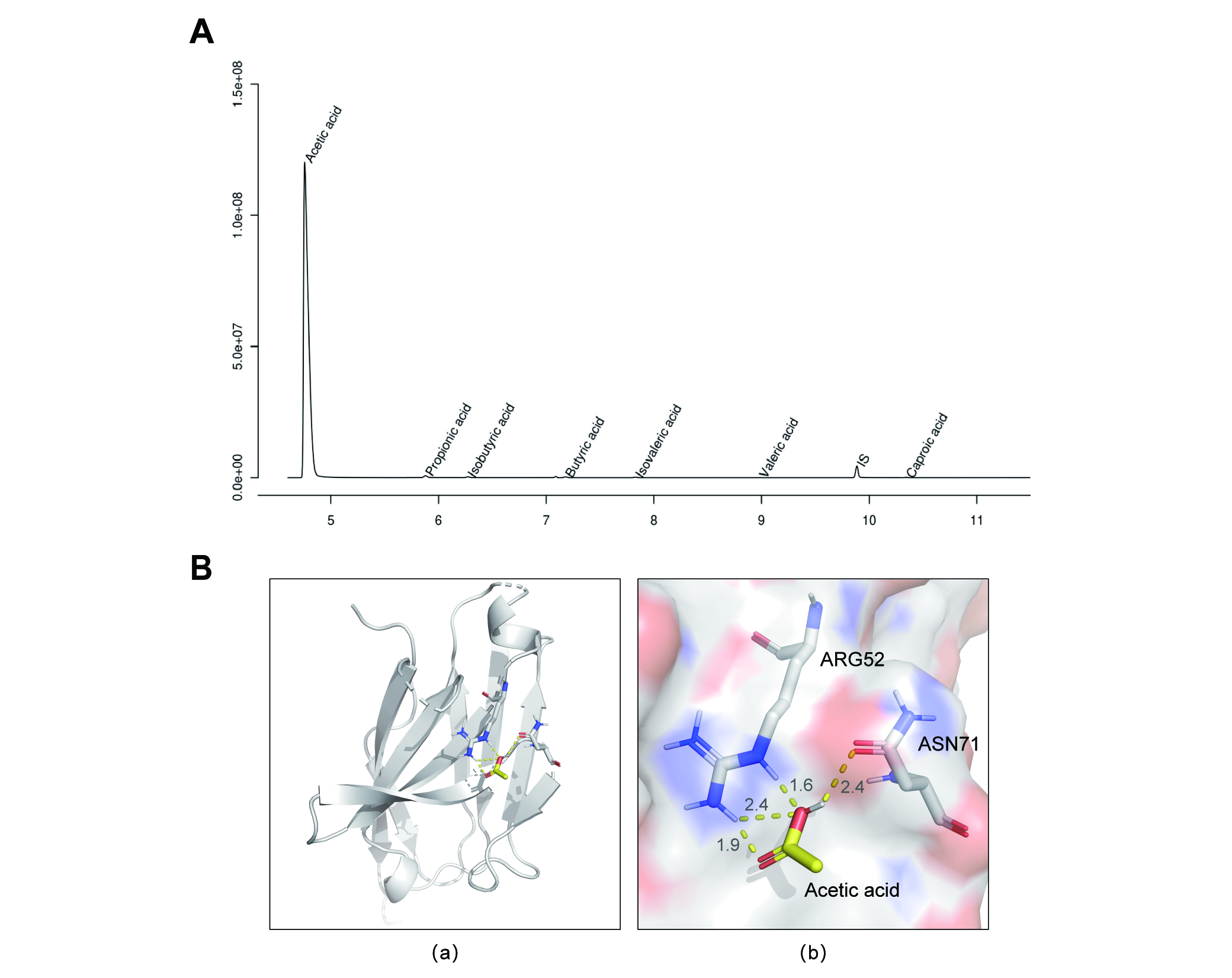

Supplement: Supplementary file 3 — fig.S3 [file 41419_2023_5589_MOESM3_ESM.tif]

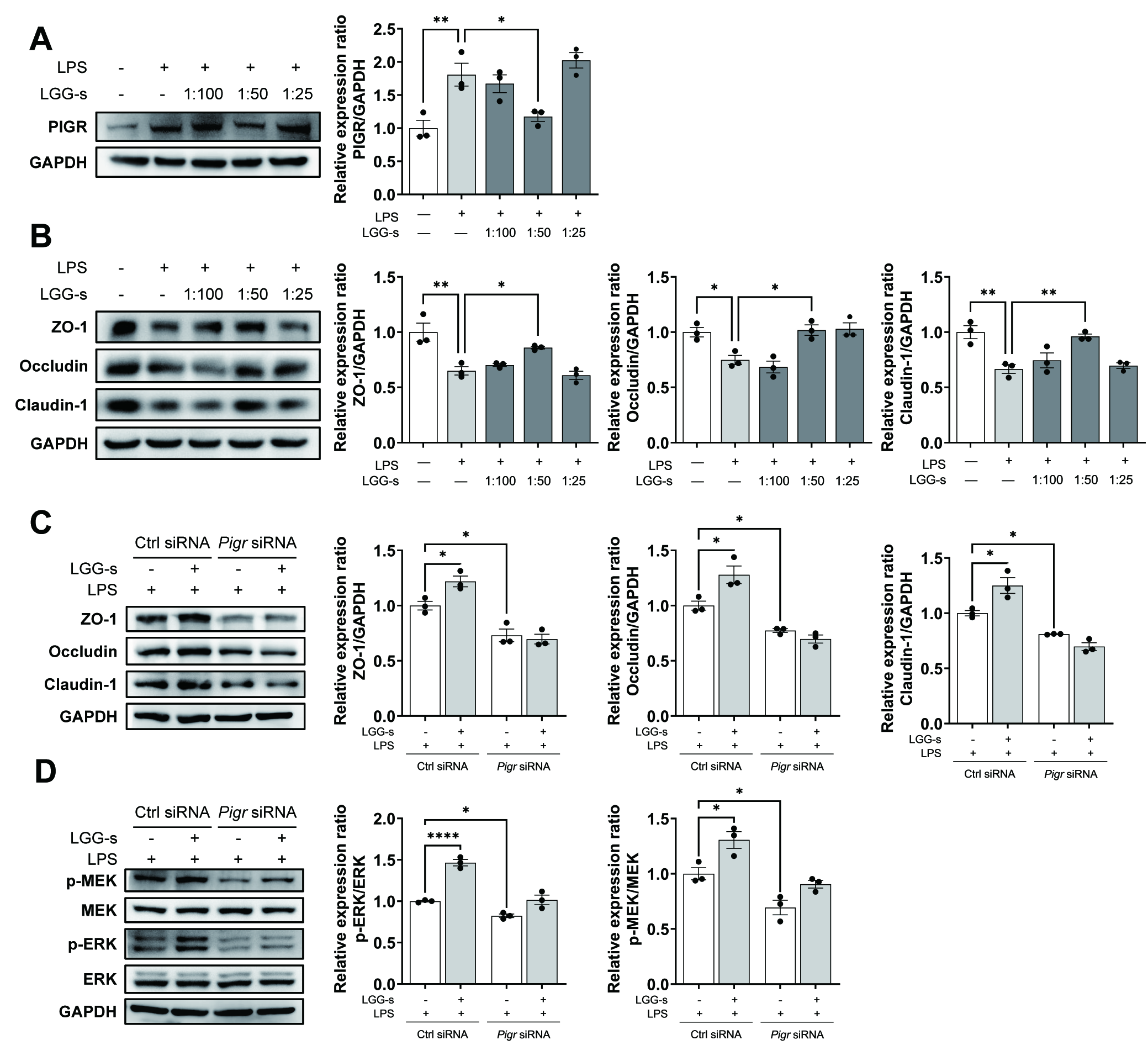

Supplement: Supplementary file 4 — fig.S4 [file 41419_2023_5589_MOESM4_ESM.tif]
