# Supplementary material for: Polymeric immunoglobulin receptor deficiency exacerbates autoimmune hepatitis by inducing intestinal dysbiosis and barrier dysfunction
Source: Cell Death Dis. 2023 Jan 28;14(1):68. doi: 10.1038/s41419-023-05589-3 (PMC9884241; doi:10.1038/s41419-023-05589-3)
Supplement: Supplementary file 5 — Supplementary Figure Legends [file 41419_2023_5589_MOESM5_ESM.docx]

**Figure. S1.** ***Pigr*^-/-^ mice have exacerbated S100-induced liver injury.** F4/80 staining of the liver. Scale bar = 100 μm.

**Figure. S2. Effects of non-absorbable broad-spectrum antibiotics (ABx) on liver injury.** (A) Liver lipopolysaccharide (LPS) level. (B) Changes in serum ALT and AST. (C) Hematoxylin and eosin (H&E) staining of the liver. Scale bar = 200 μm.

**Figure. S3. Acetic acid binds pIgR protein.** (A) The SCFAs levels in LGG-s. (B) (a) Schematic diagram of acetic acid-binding pIgR protein (gray cartoon). (b) The binding pose of acetic acid with the lowest binding energy is shown for pIgR. The carbon atoms of two key residues, side chain and acetic acid, are represented as gray and yellow sticks, respectively. The hydrogen bonds are shown as a yellow chain.

**Figure. S4. Effects of *Pigr* deletion and LGG-s on intestinal MEK/ERK phosphorylation.** (A, B) Exploring the optimal concentration of LGG-s. (C) The effect of *Pigr* in improving barrier damage by LGG-s in LPS-induced Caco-2. (D) Effects of *Pigr*^-/-^ on MEK/ERK phosphorylation in LPS-induced Caco-2. Western blotting analysis for pIgR, ZO-1, Occludin, and Claudin-1 normalized by GAPDH. Western blotting analysis for p-MEK and p-ERK normalized by MEK and ERK. Data are presented as mean ± SEM. Statistical analysis was performed using one-way ANOVA with Tukey multiple comparisons.
