# Supplementary material for: Polymeric immunoglobulin receptor deficiency exacerbates autoimmune hepatitis by inducing intestinal dysbiosis and barrier dysfunction
Source: Cell Death Dis. 2023 Jan 28;14(1):68. doi: 10.1038/s41419-023-05589-3 (PMC9884241; doi:10.1038/s41419-023-05589-3)
Supplement: Supplementary file 6 — Supplementary Table 1 [file 41419_2023_5589_MOESM6_ESM.docx]

| **Supplementary Table 1. The Oligonucleotide primers used in real-time PCR analysis** | |
| --- | --- |
| **Name** | **Sequence** |
| *Zo-1* | F: GGGCCATCTCAACTCCTGTA |
|  | R: AGAAGGGCTGACGGGTAAAT |
| *Occludin* | F: ACTATGCGGAAAGAGTTGACAG |
|  | R: GTCATCCACACTCAAGGTCAG |
| *Claudin-1* | F: GGGGACAACATCGTGACCG |
|  | R: AGGAGTCGAAGACTTTGCACT |
| *Tnf-α* | F: ATCTACCTGGGAGGCGTCTT |
|  | R: GAGTGGCACAAGGAACTGGT |
| *Il-6* | F: TGGAAATGAGAAAAGAGTTGTGC |
|  | F: TGGAAATGAGAAAAGAGTTGTGC |
| *Il-1β* | F: TTCATCTTTGAAGAAGAGCCCAT |
|  | R: TCGGAGCCTGTAGTGCAGTT |
| *Pigr* | F: GCTCCAAAGTGCTGTTCTCC |
|  | R: TTGCTGTGTGTCTGGAGAGG |
| *Actb* | F: CCTCACTGTCCACCTTCC |
|  | R: GGGTGTAAAACGCAGCTC |
| *16S* | F: ACTCCTACGGGAGGCAGCAGT |
|  | R: ATTACCGCGGCTGCTGGC |
| *Anaeromassilibacillus* | F: TGGATGAGGATGAGGAGTAAG |
|  | R: GAGCGAAAAGACGAACACA |
