# Supplementary material for: Polymeric immunoglobulin receptor deficiency exacerbates autoimmune hepatitis by inducing intestinal dysbiosis and barrier dysfunction
Source: Cell Death Dis. 2023 Jan 28;14(1):68. doi: 10.1038/s41419-023-05589-3 (PMC9884241; doi:10.1038/s41419-023-05589-3)
Supplement: Supplementary file 8 — Original Data File-Western blot [file 41419_2023_5589_MOESM8_ESM.pdf]

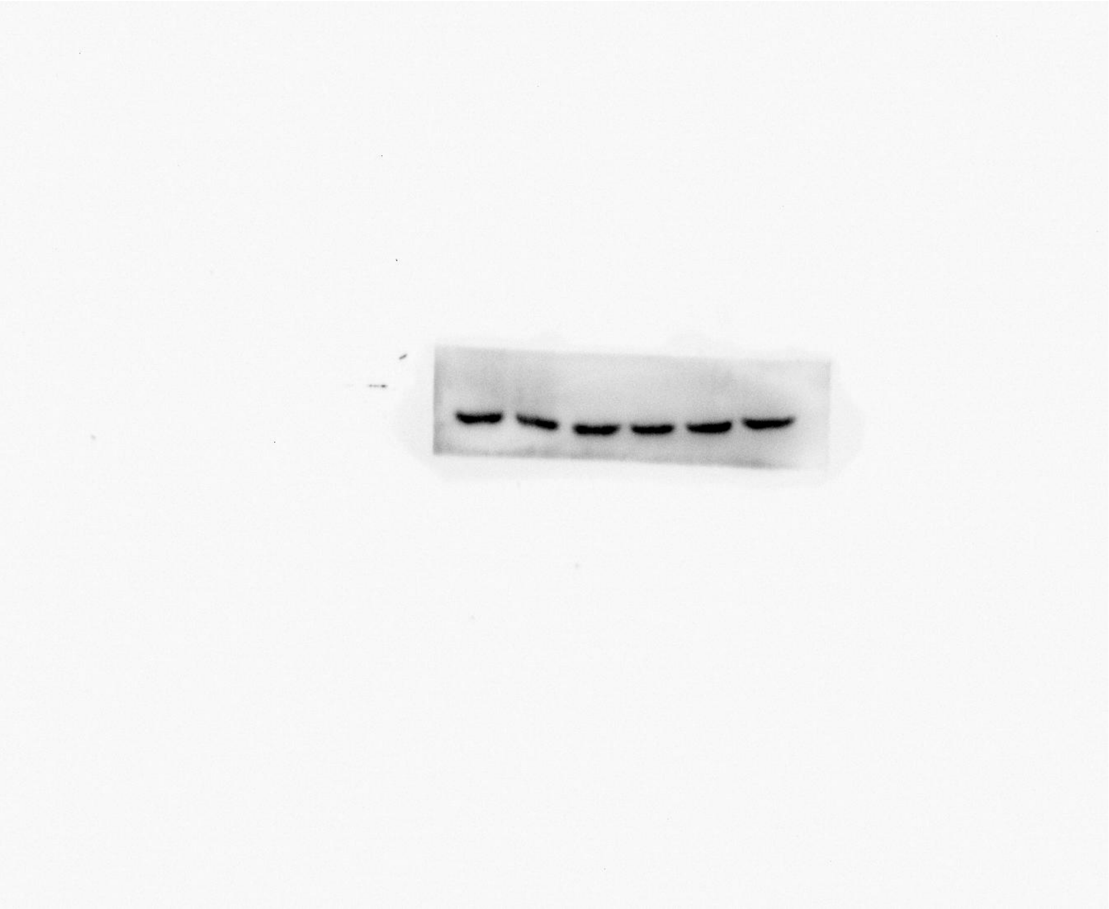

1.fig.1C-1-gap

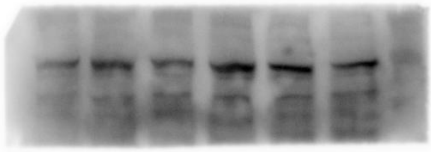

2.fig.1C-1-pigr

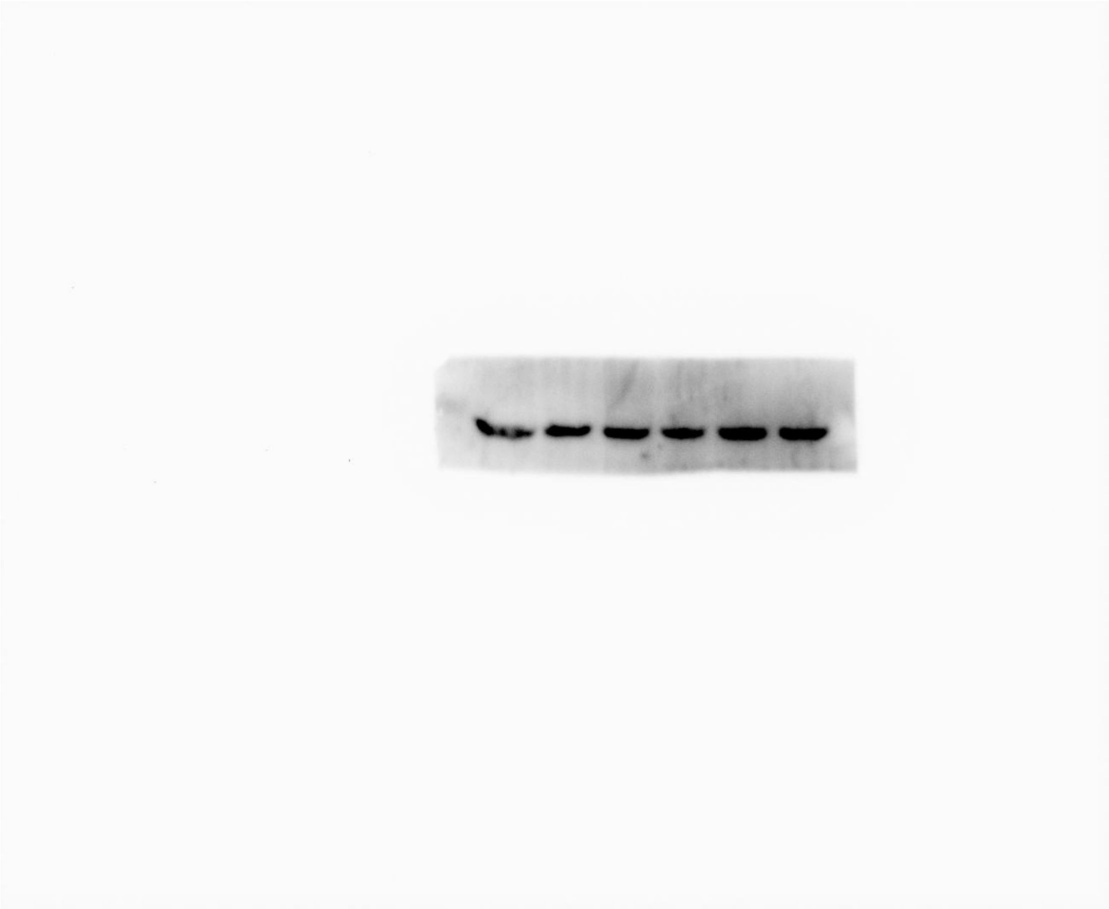

3.fig.1C-2-gap

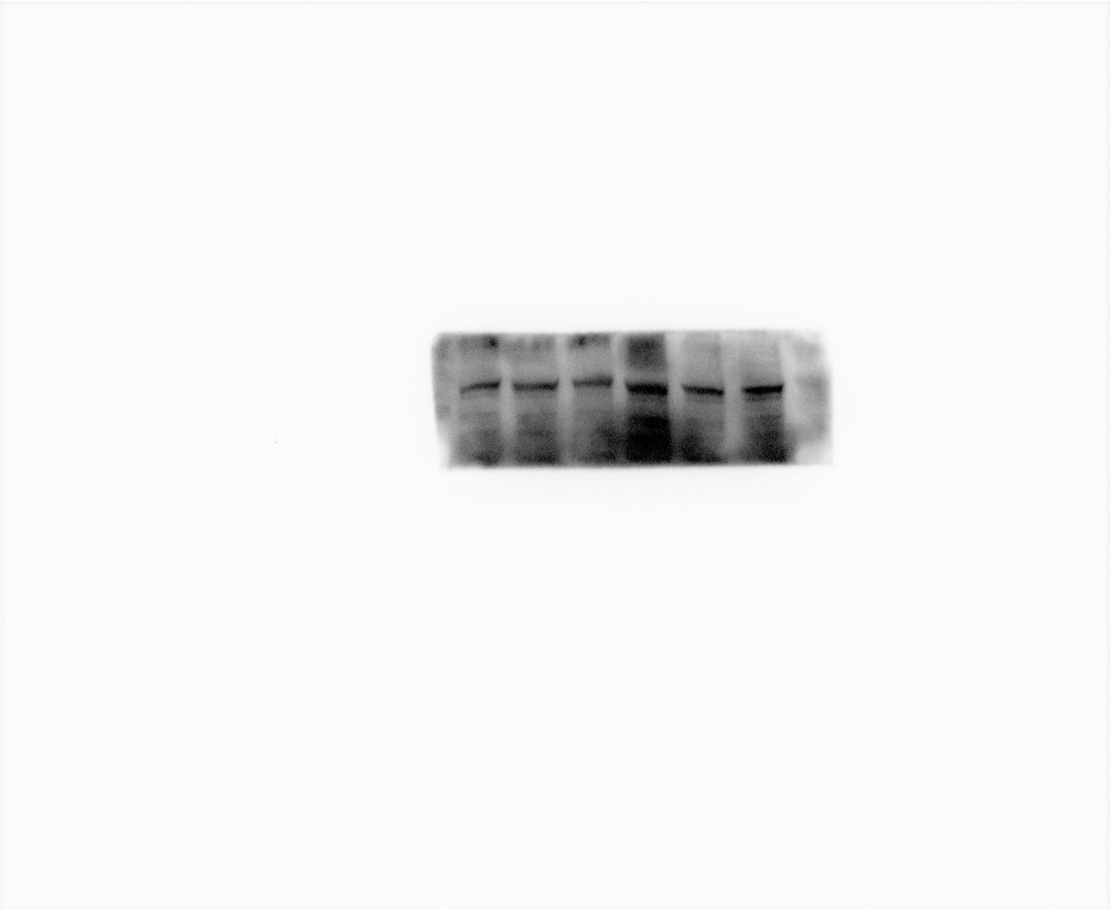

4.fig.1C-2-pigr

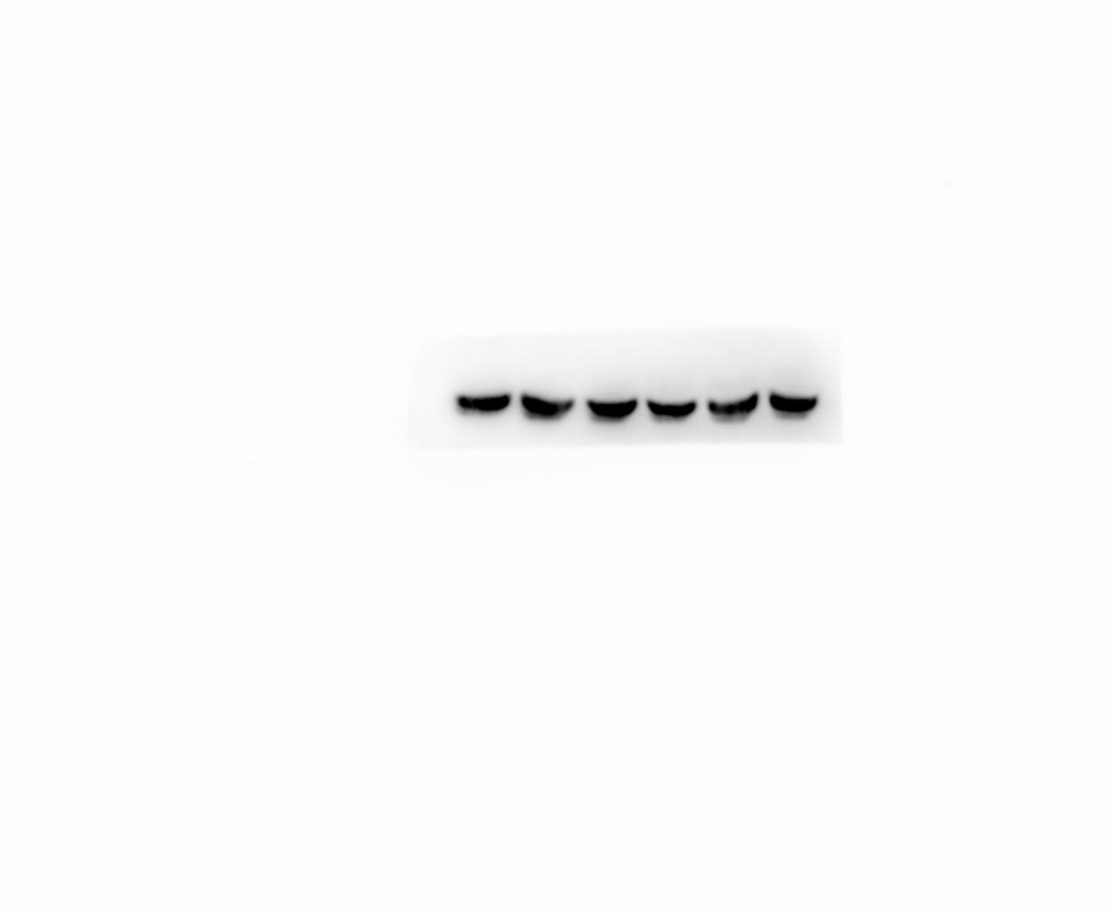

5.fig.1C-3-gap

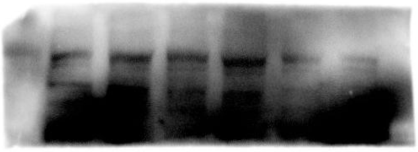

6.fig.1C-3-pigr

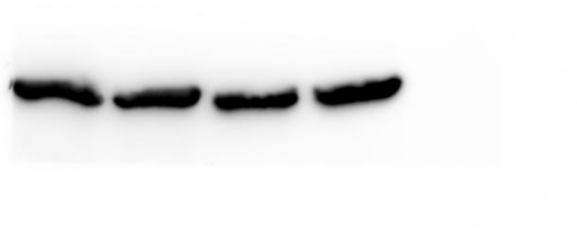

7.fig.1G-1-gap

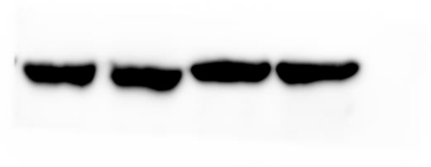

8.fig.1G-2-gap

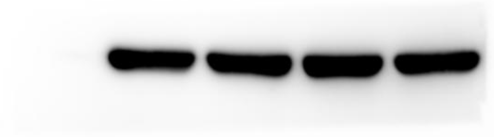

9.fig.1G-3-gap

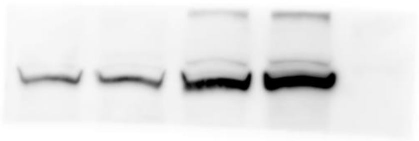

10.fig.1G-1-pigr

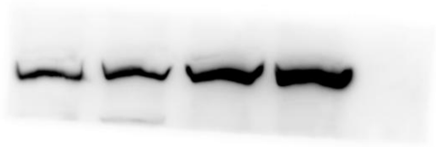

11.fig.1G-2-pigr

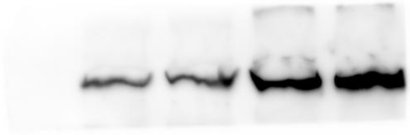

12.fig.1G-3-pigr

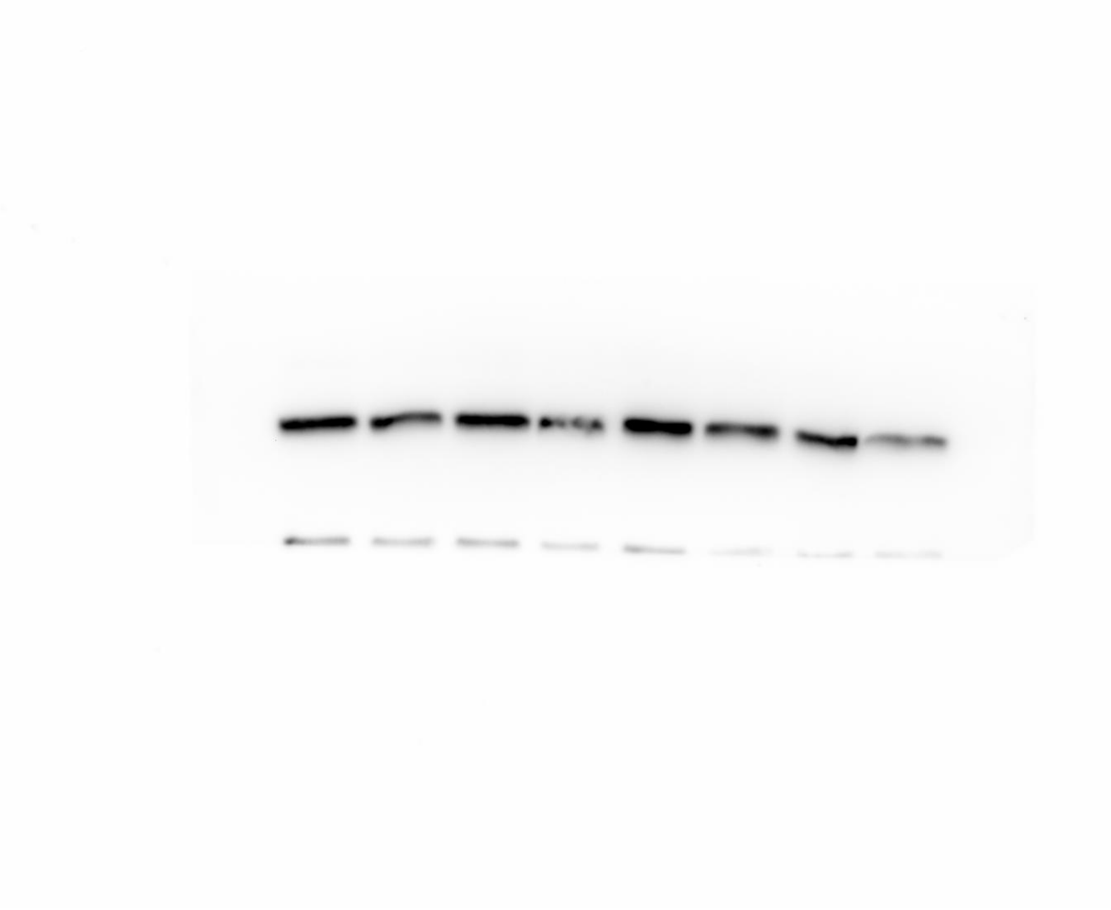

13.fig.4E-1-claudin-left

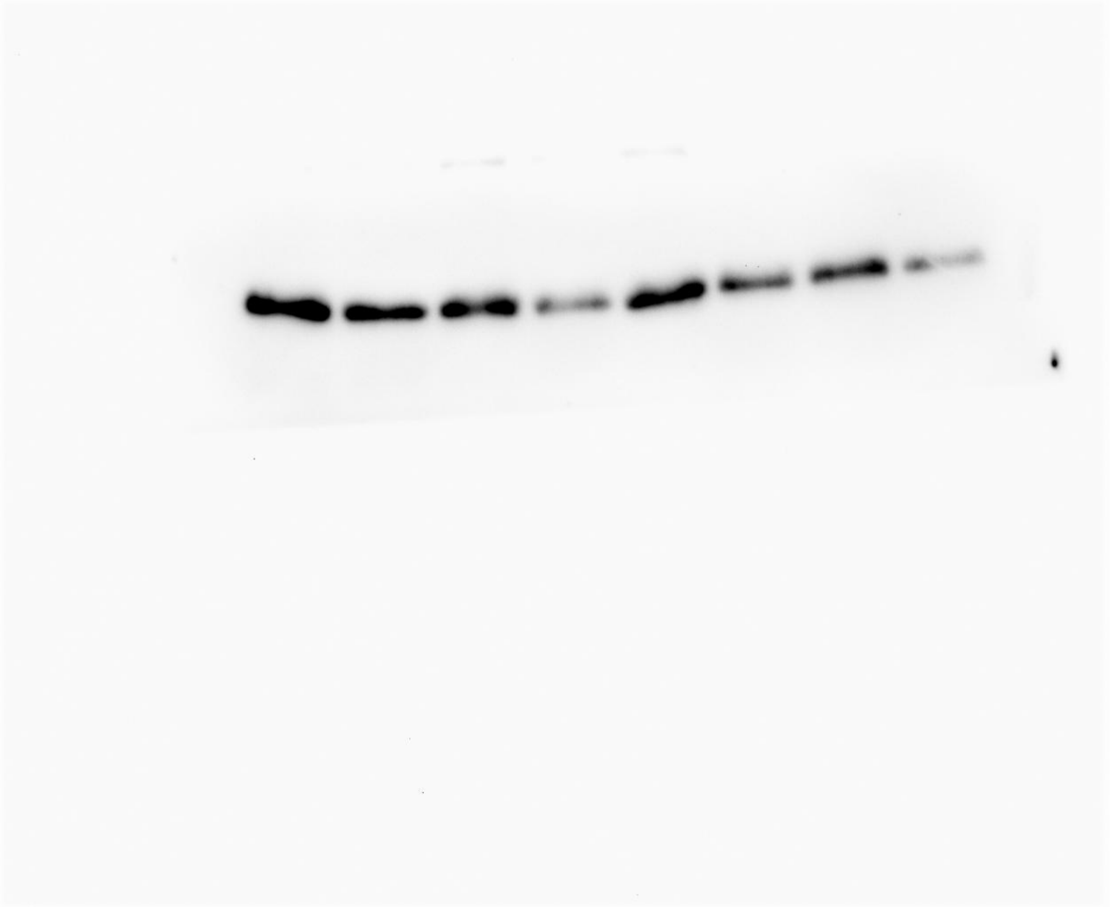

14.fig.4E-2-claudin-left

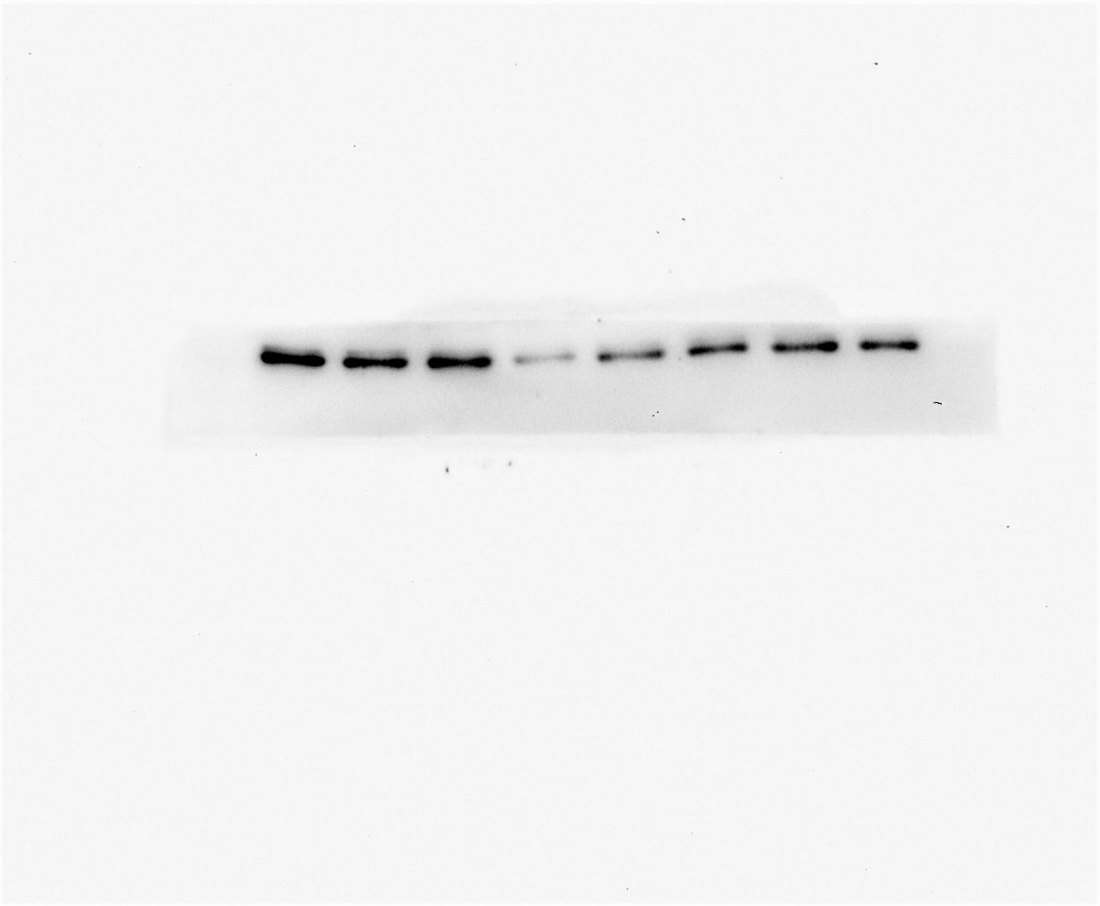

15.fig.4E-3-claudin-left

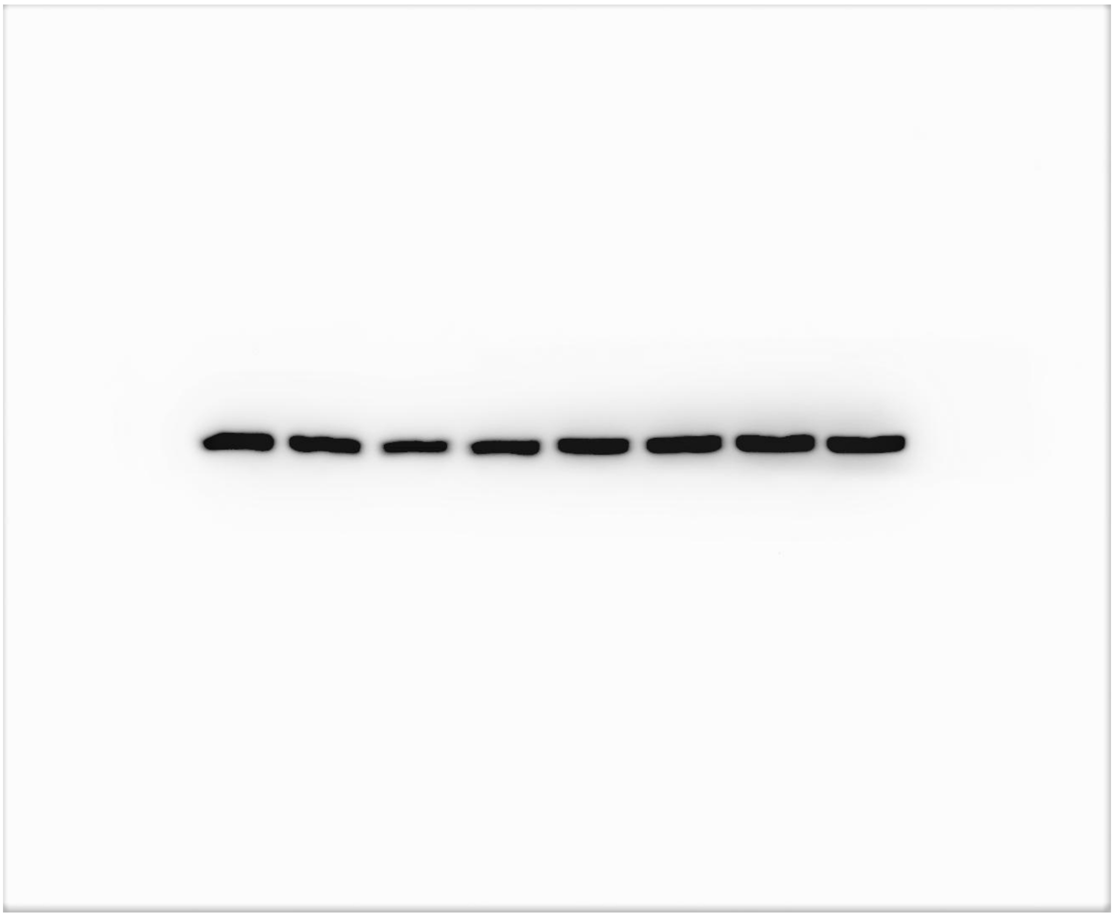

16.fig.4E-1-gap-right

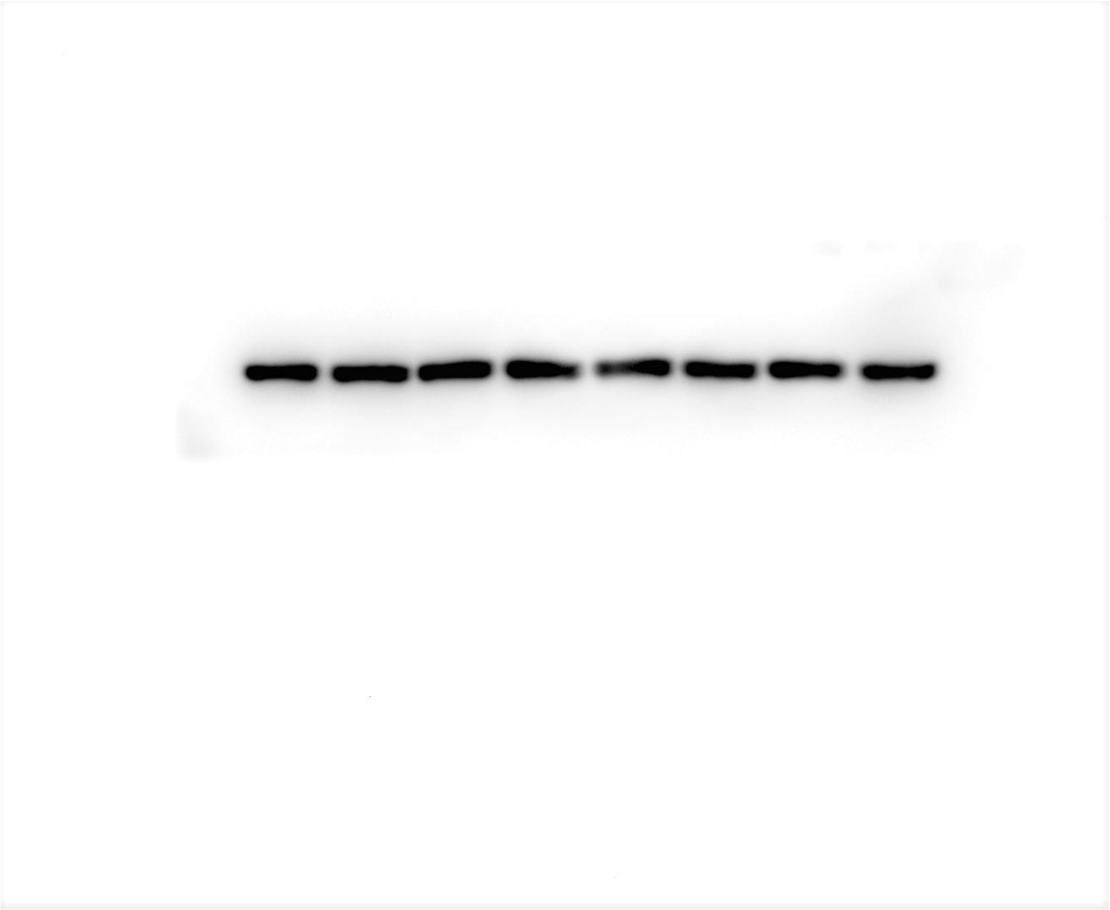

17.fig.4E-2-gap-left

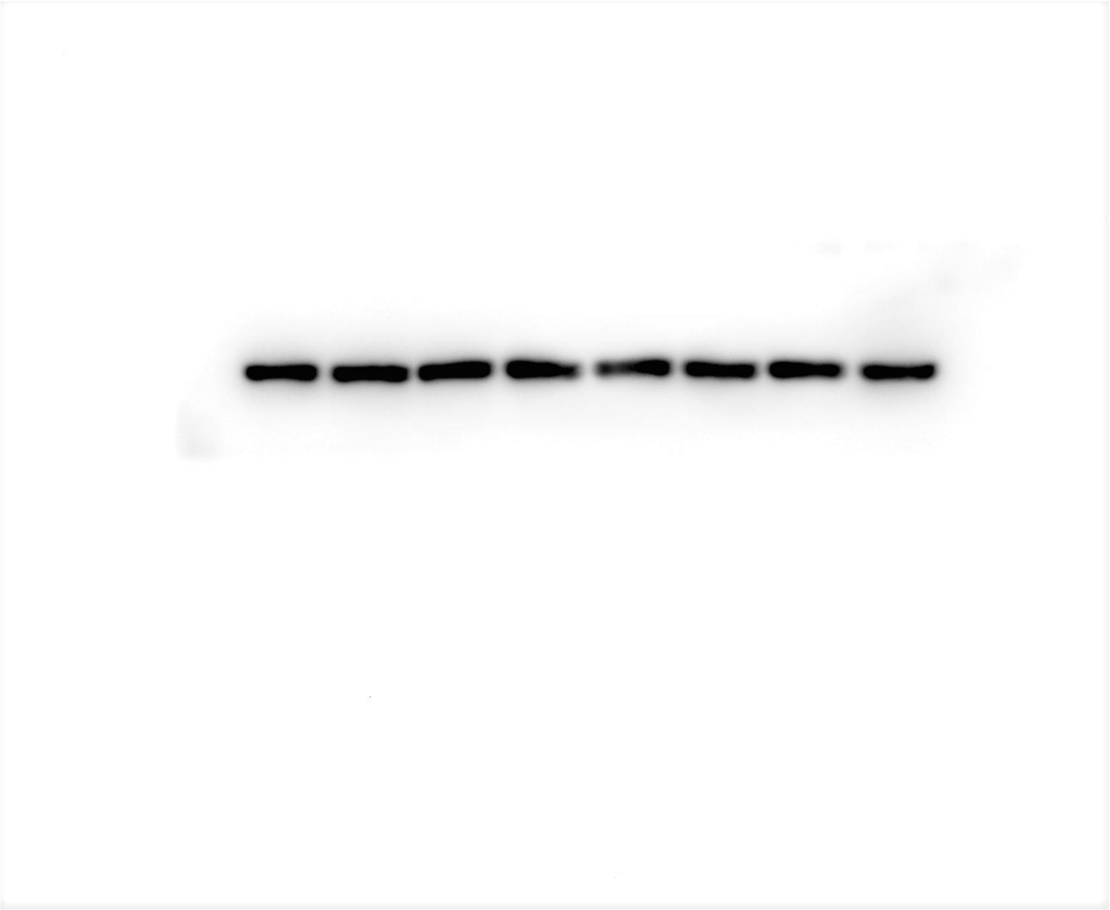

18.fig.4E-3-gap-right

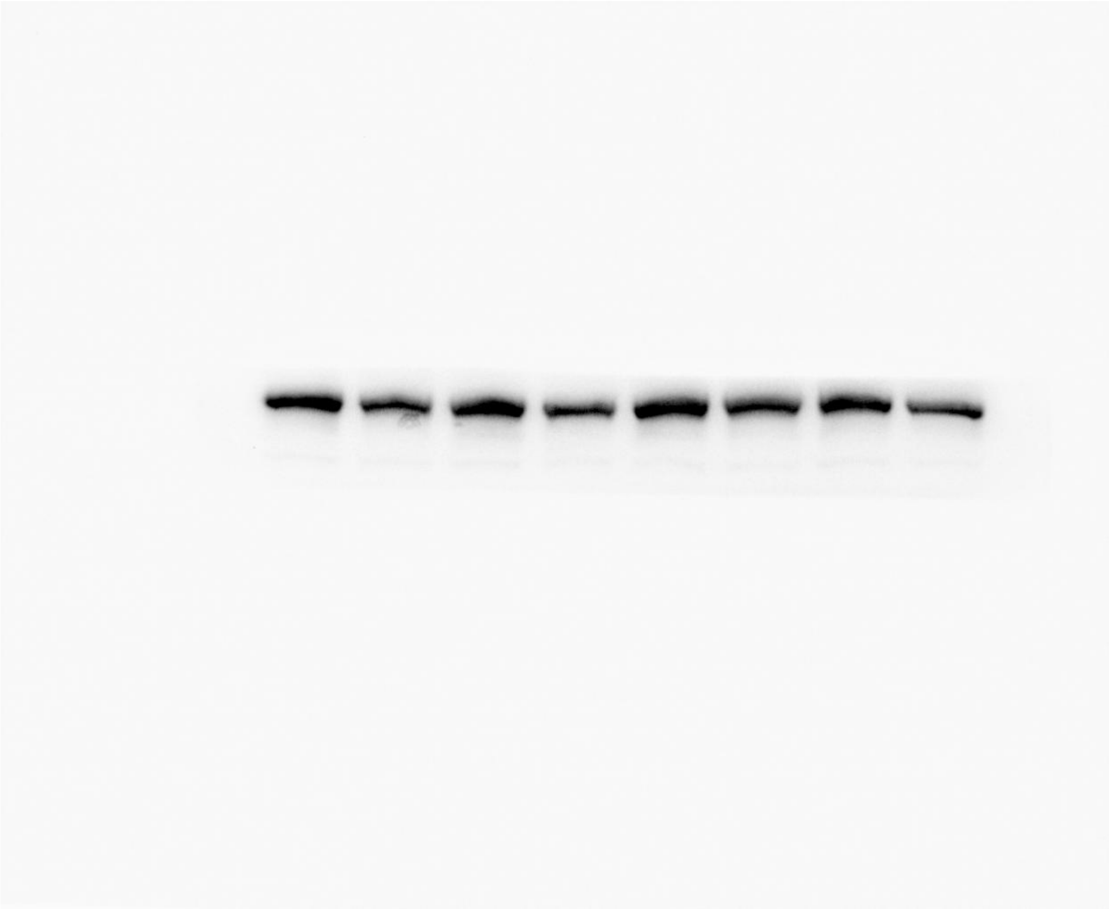

19.fig.4E-1-occludin-right

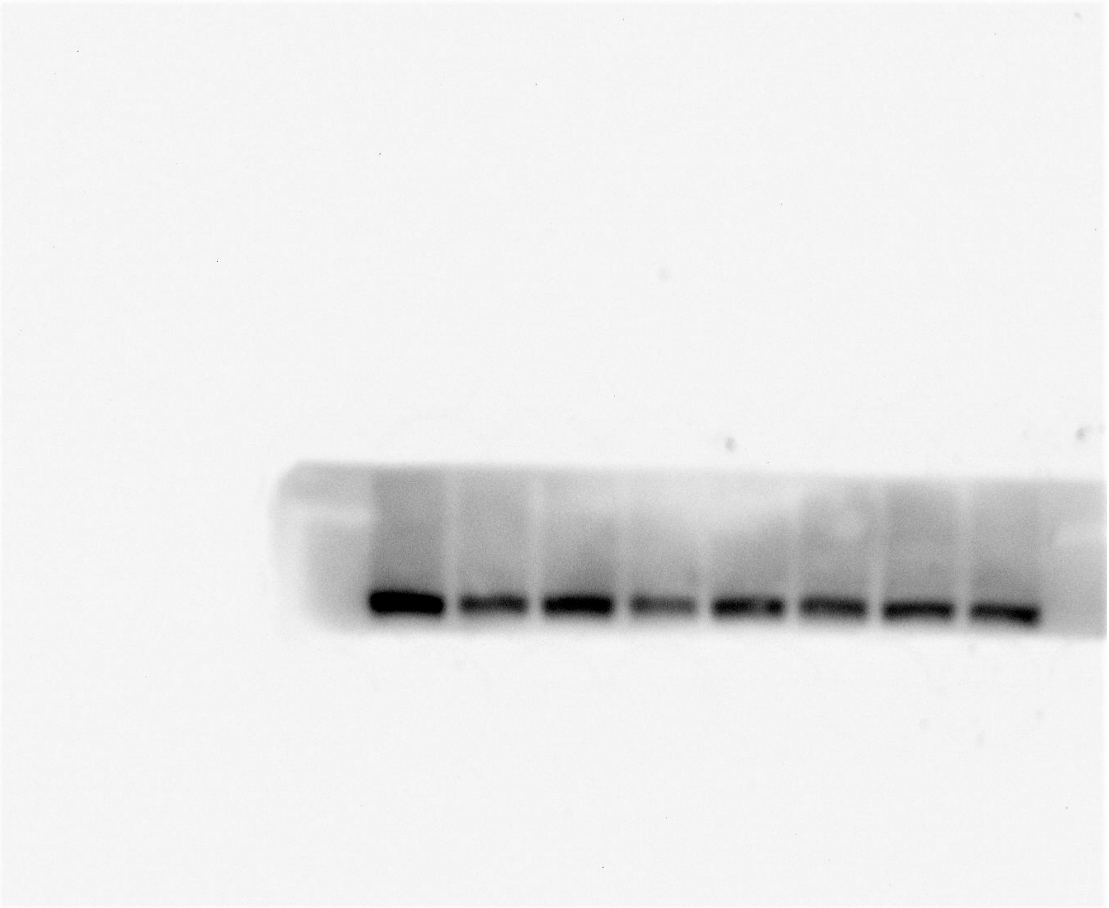

20.fig.4E-2-occludin-left

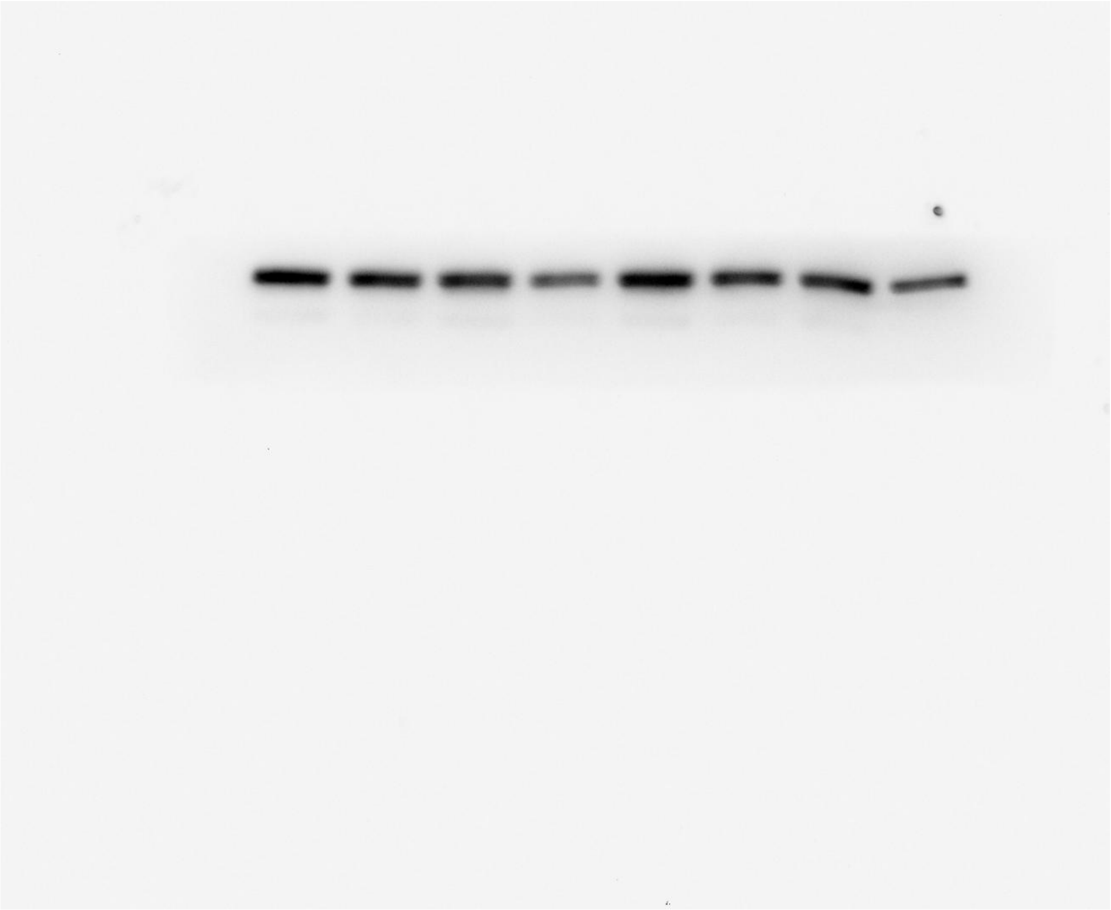

21.fig.4E-3-occludin-right

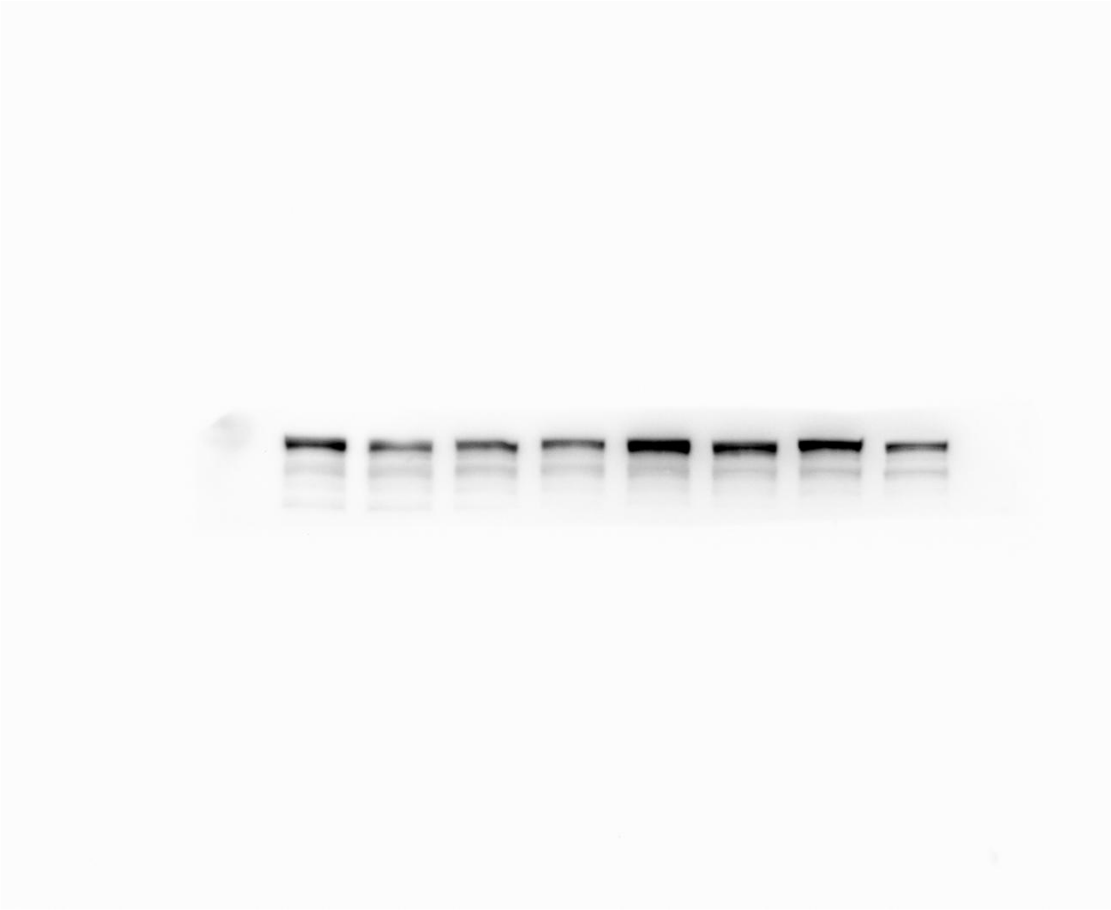

22.fig.4E-1-zo-right

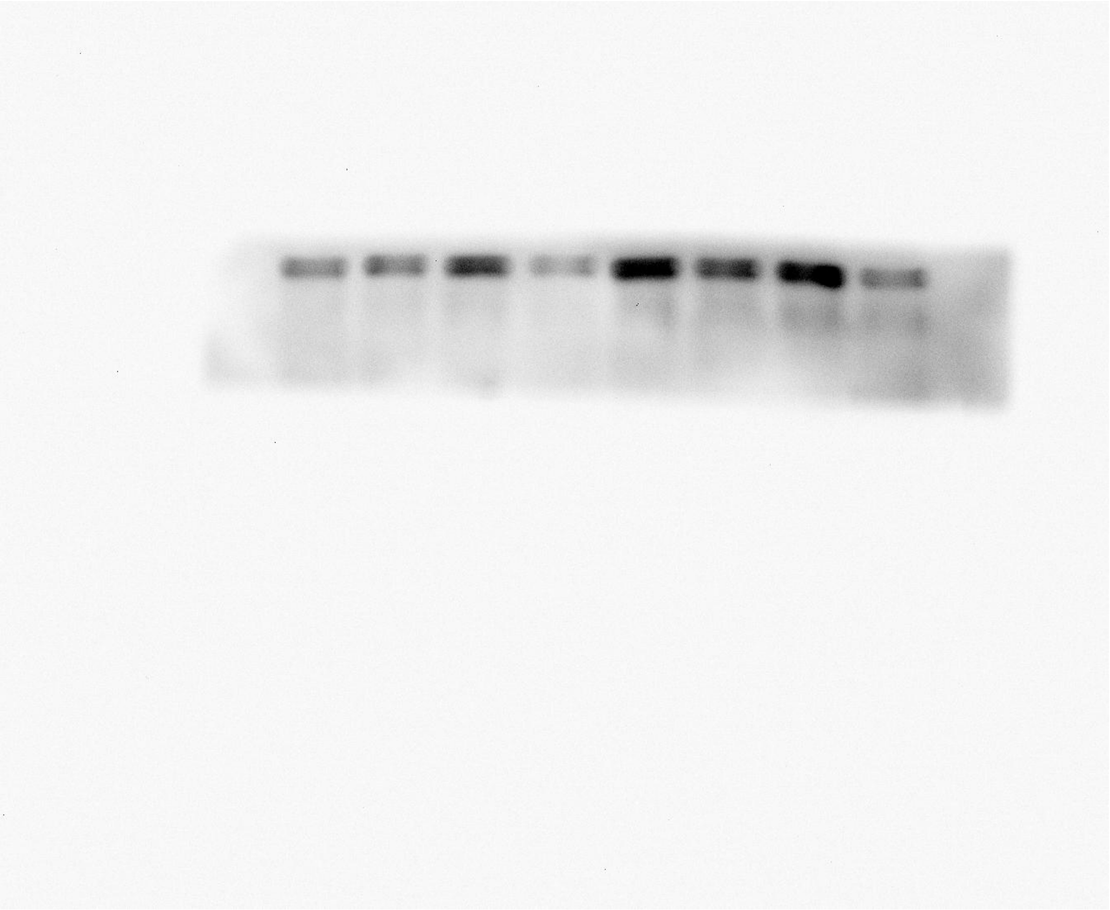

23.fig.4E-2-zo-right

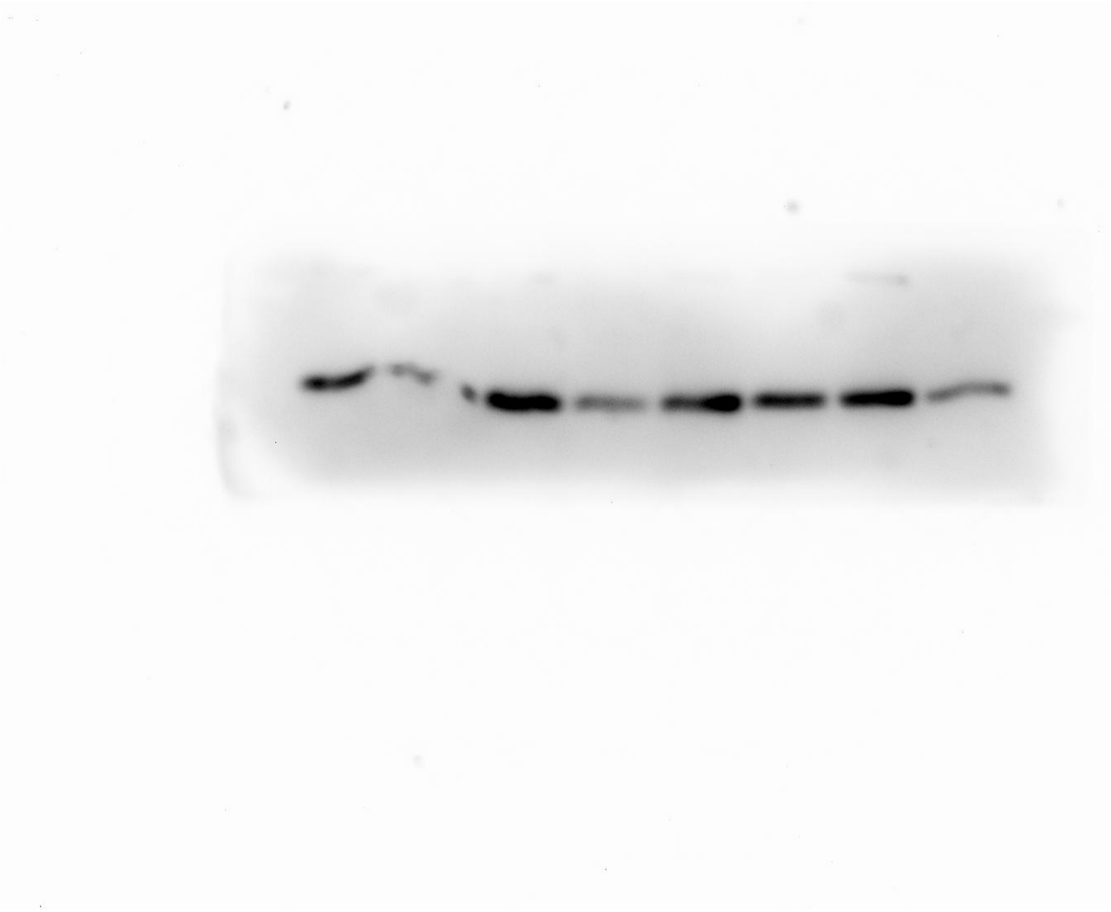

24.fig.4E-3-zo-right

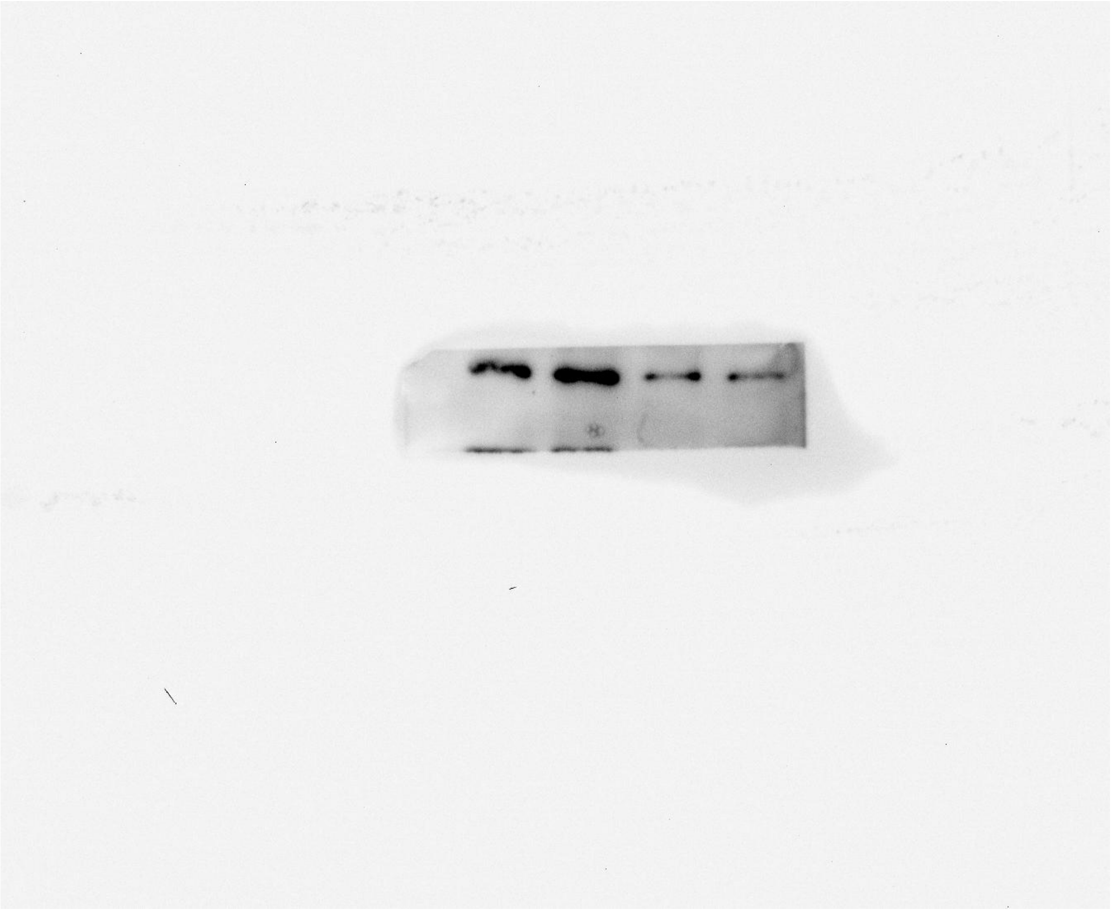

25.fig.5J-1-claudin

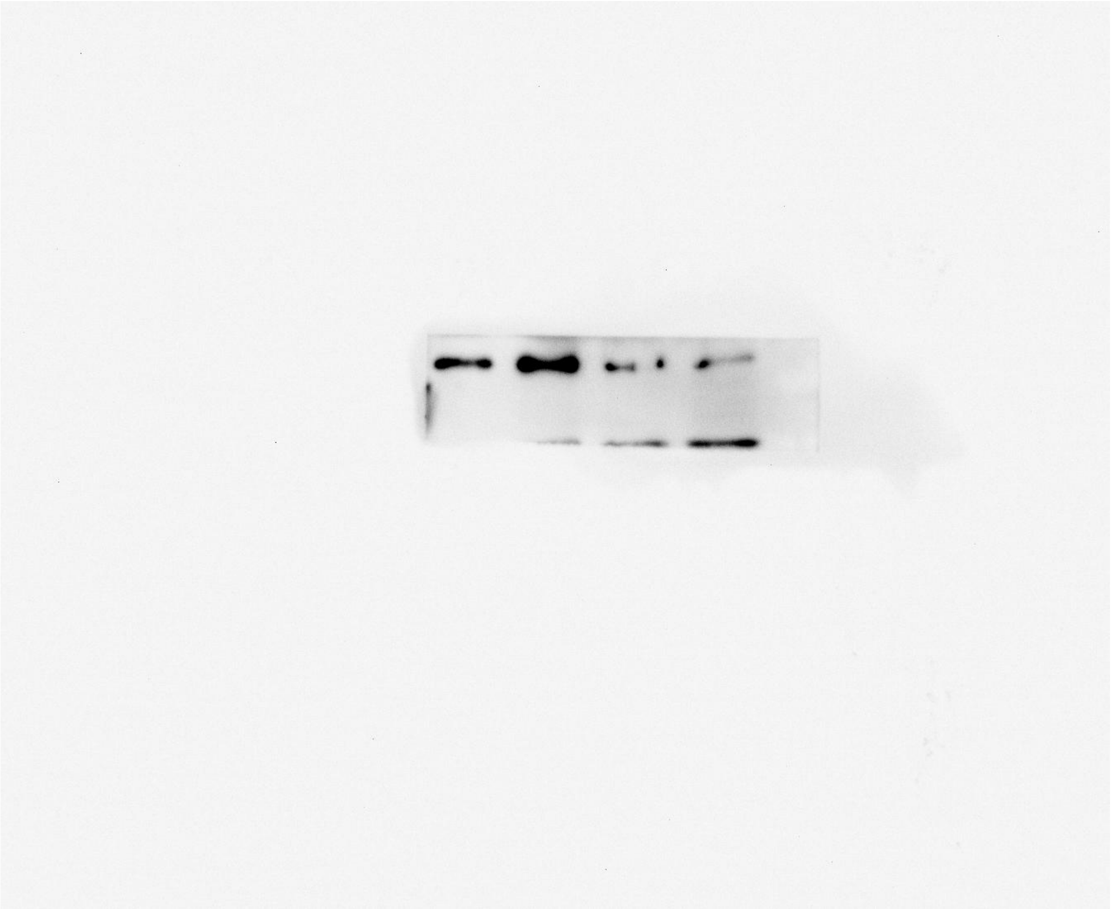

26.fig.5J-2-claudin

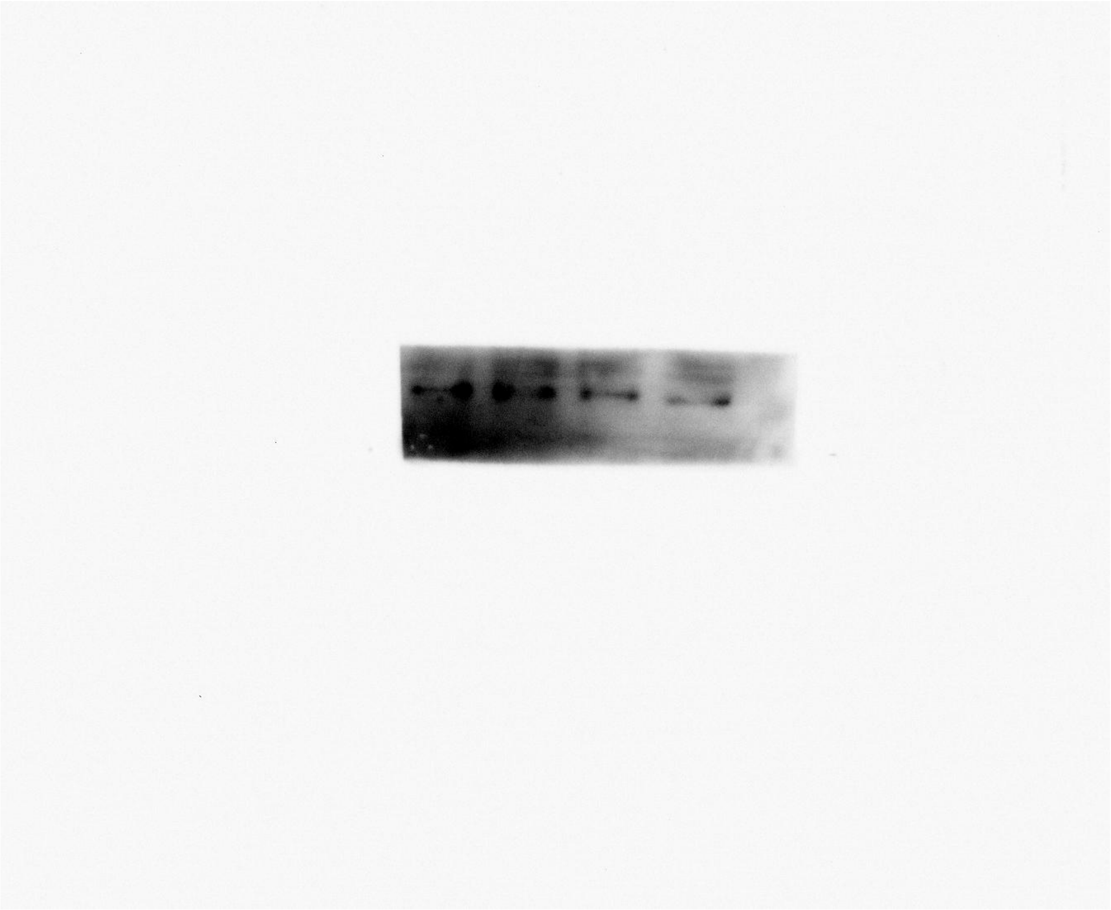

27.fig.5J-3-claudin

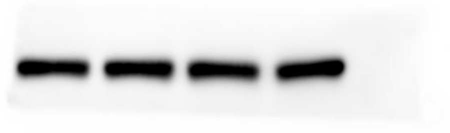

28.fig.5J-1-gap

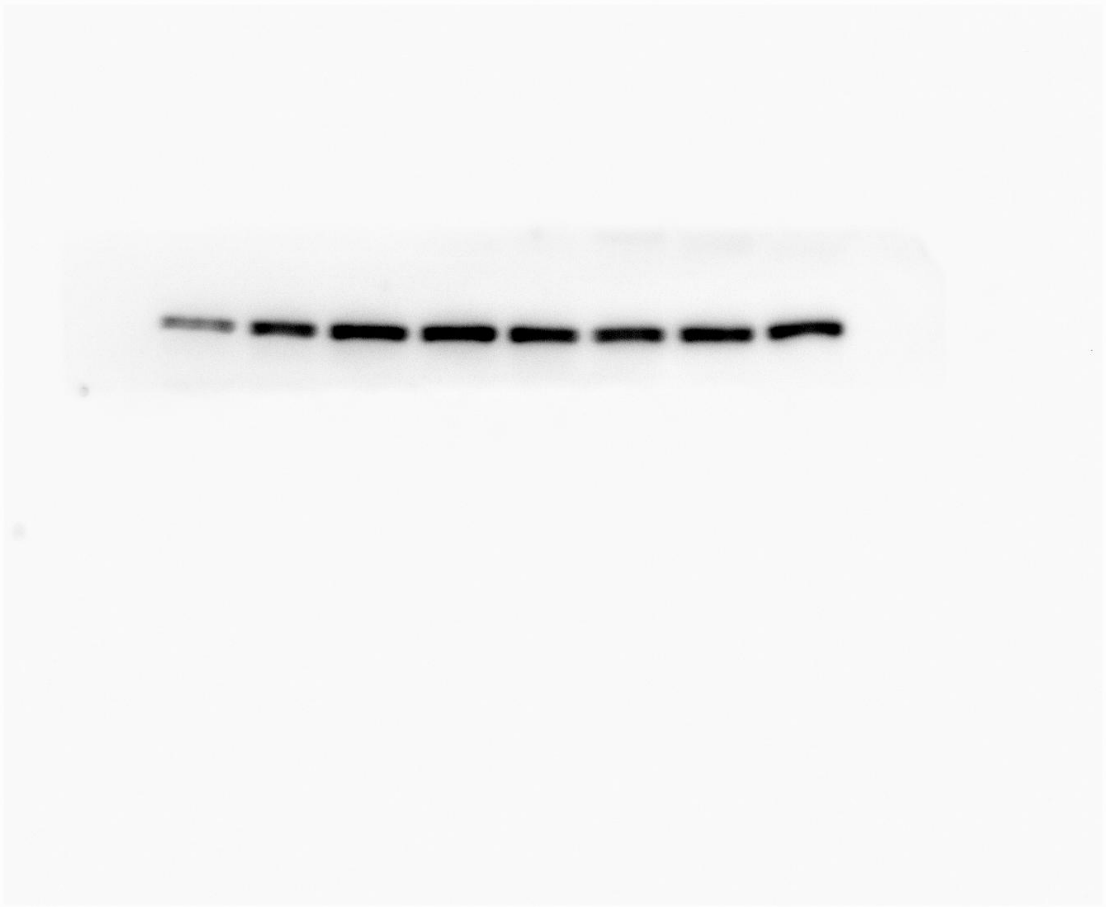

29.fig.5J-2-gap-right

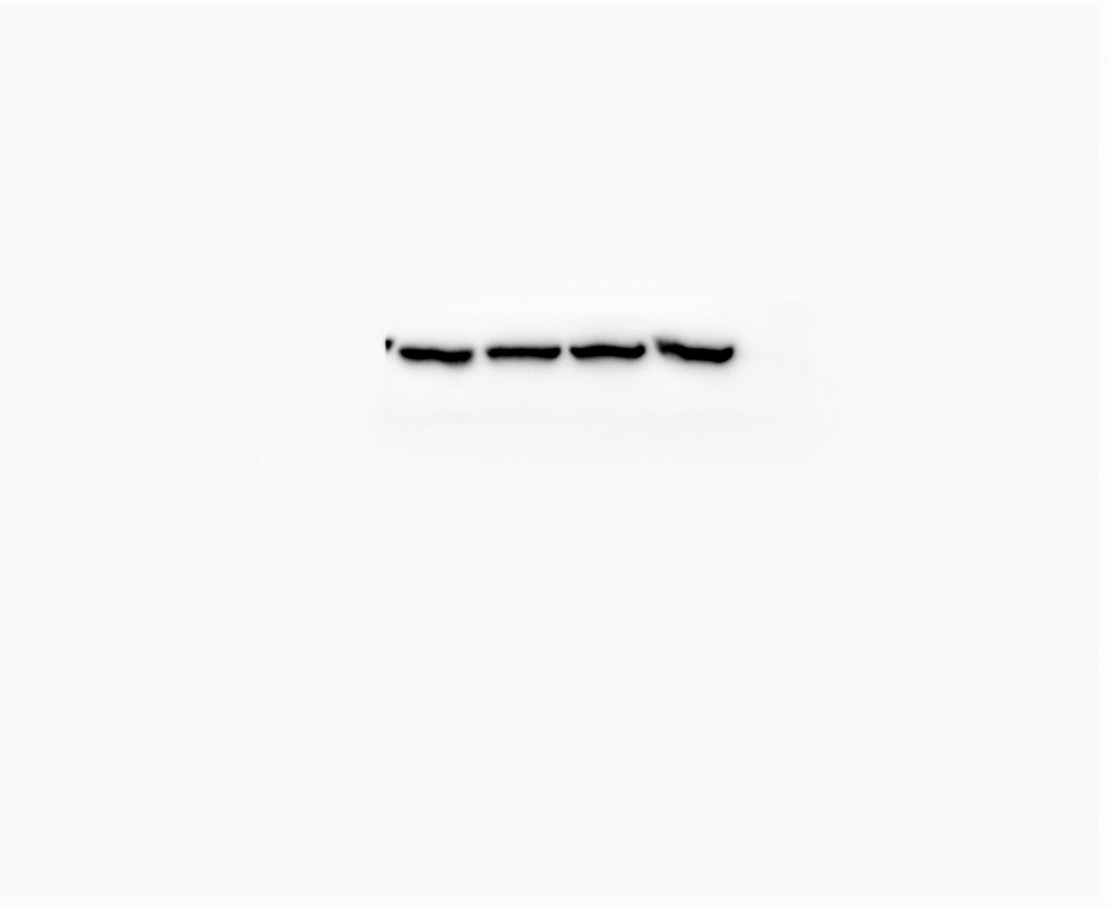

30.fig.5J-3-gap

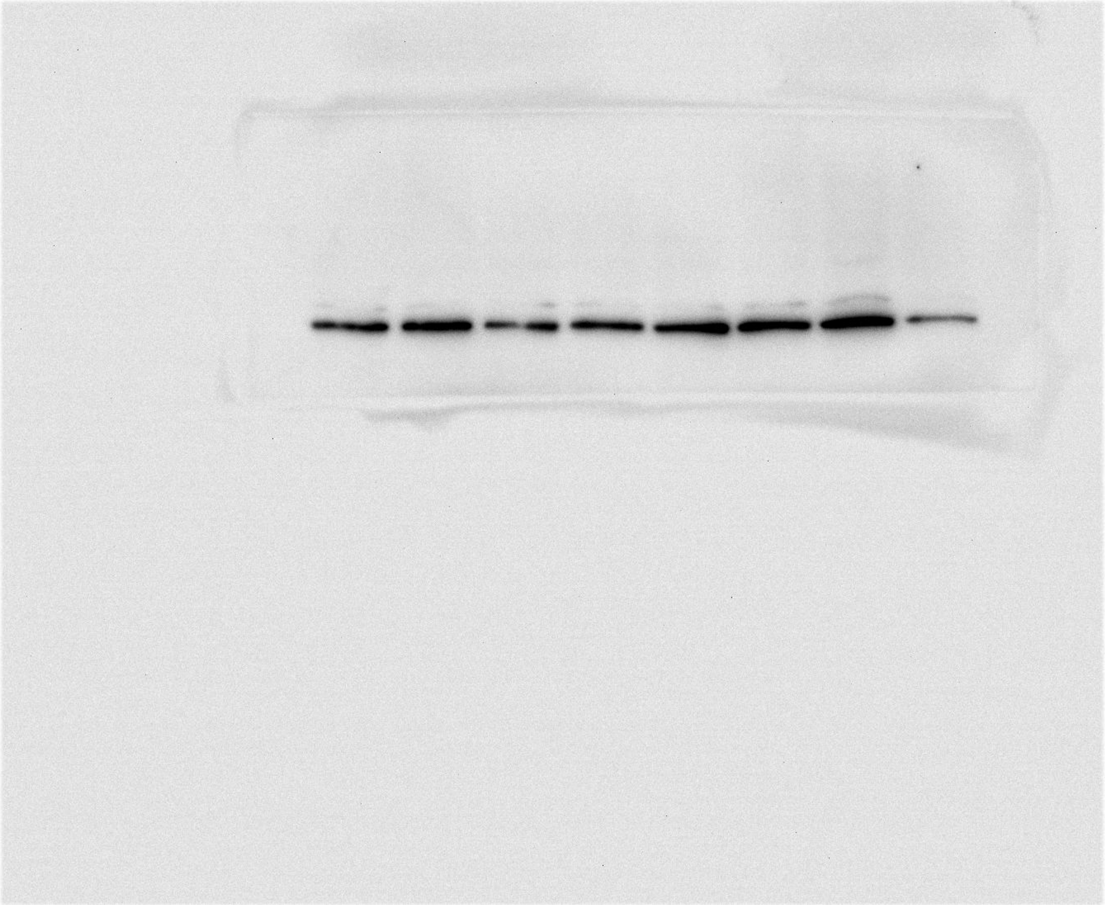

31.fig.5J-1-occludin-left

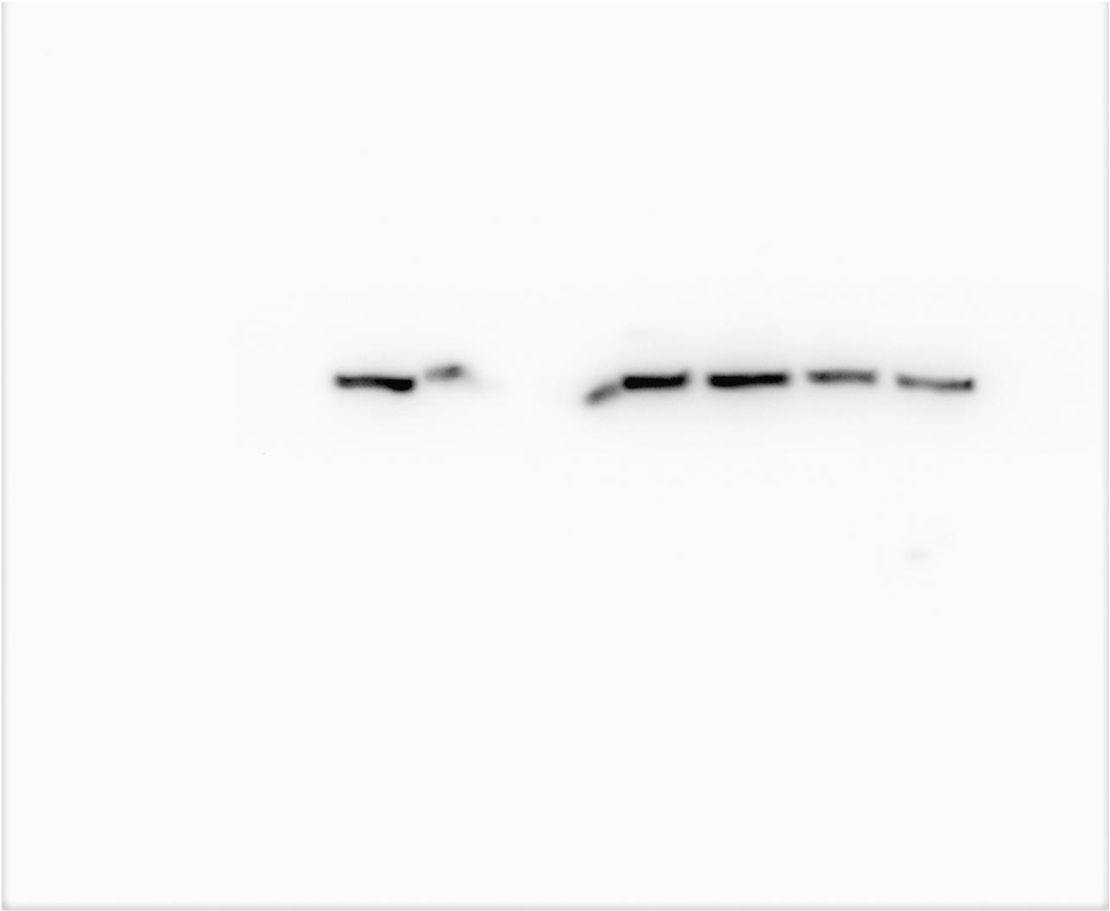

32.fig.5J-2-occludin-right

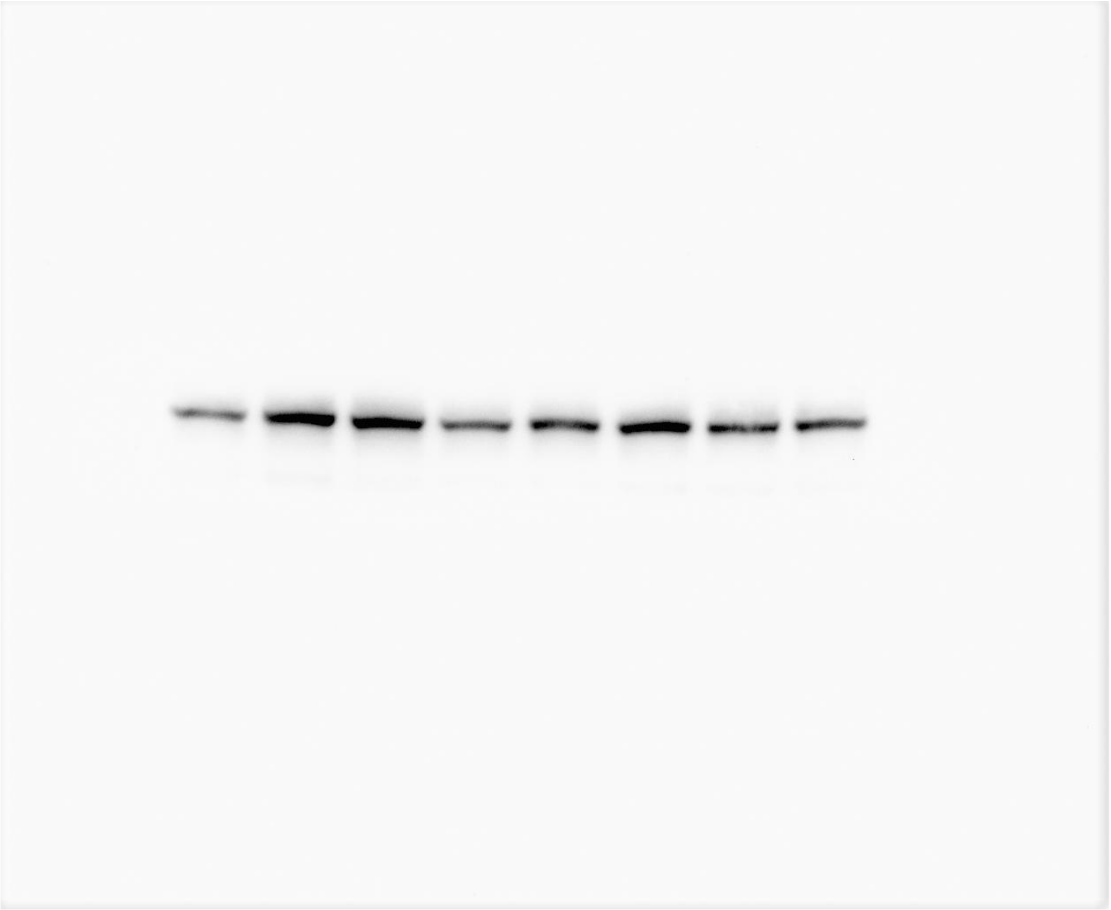

33.fig.5J-3-occludin-right

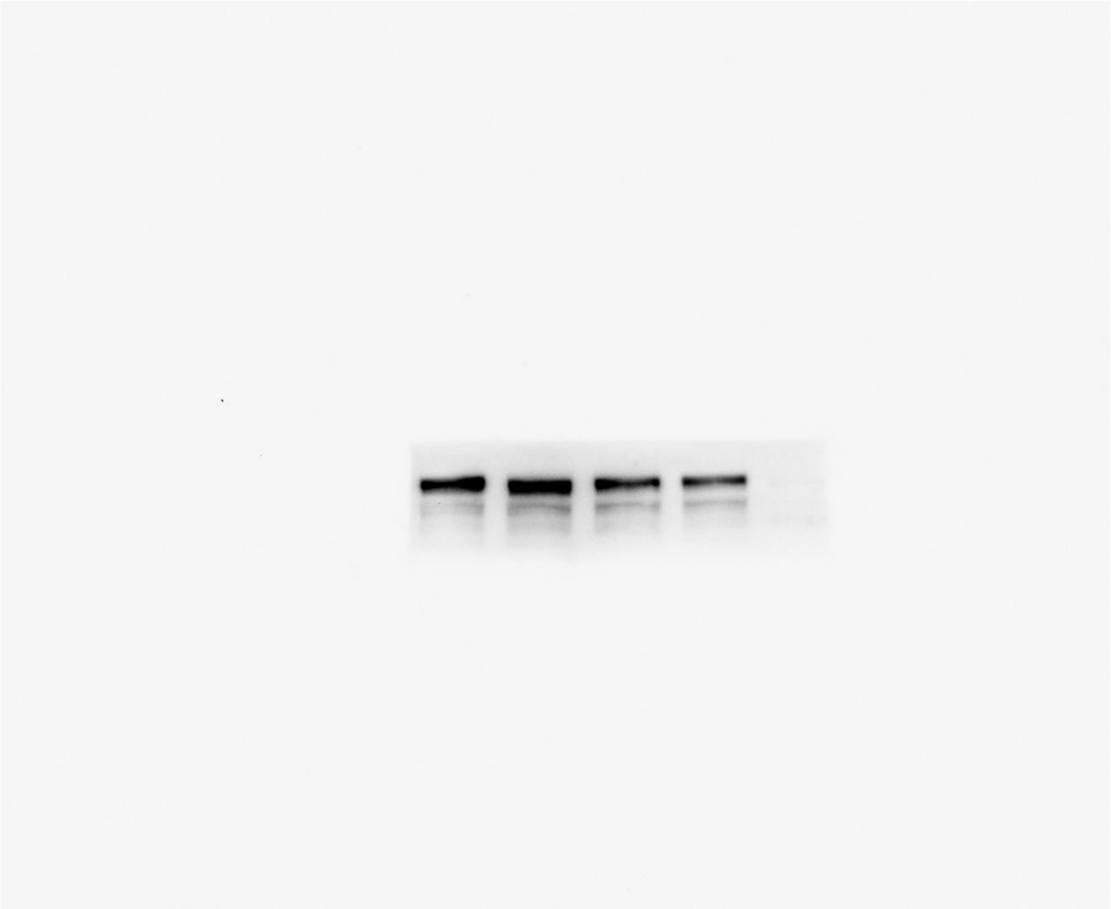

34.fig.5J-1-zo

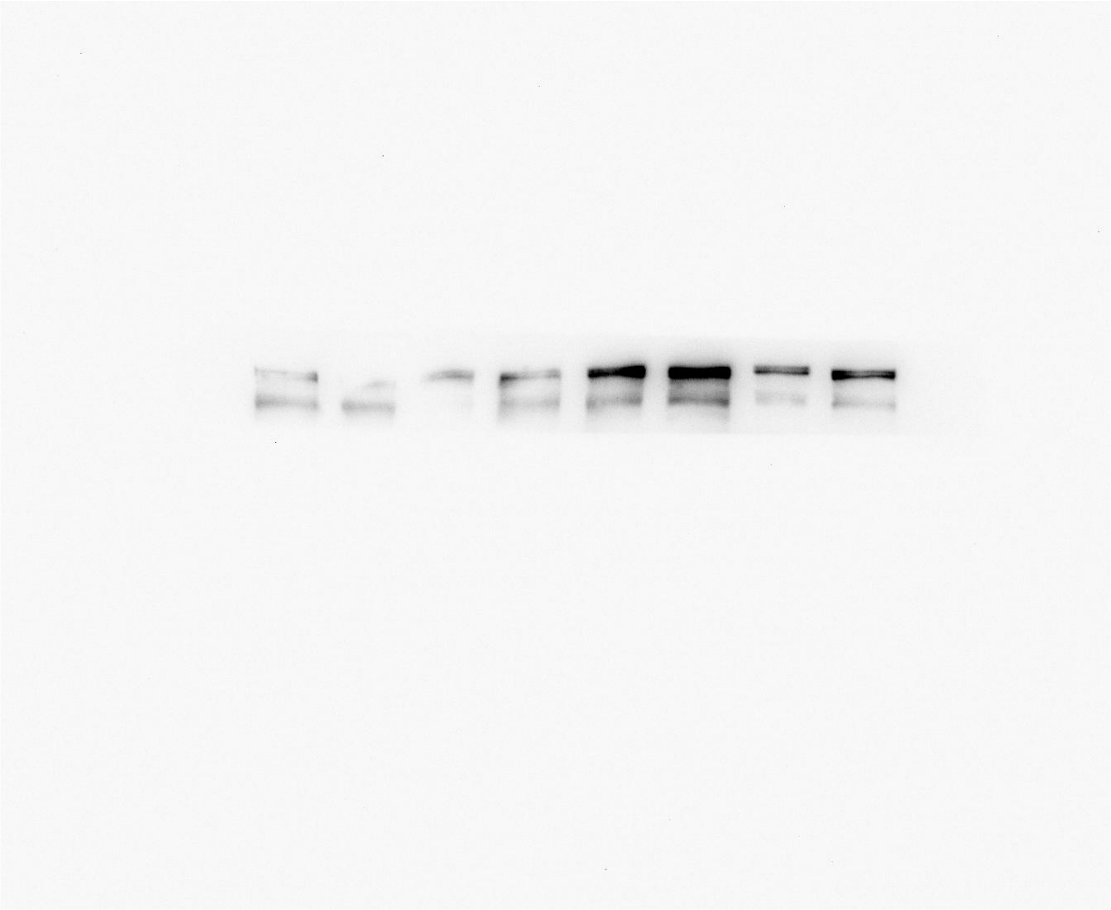

35.fig.5J-2-zo-right

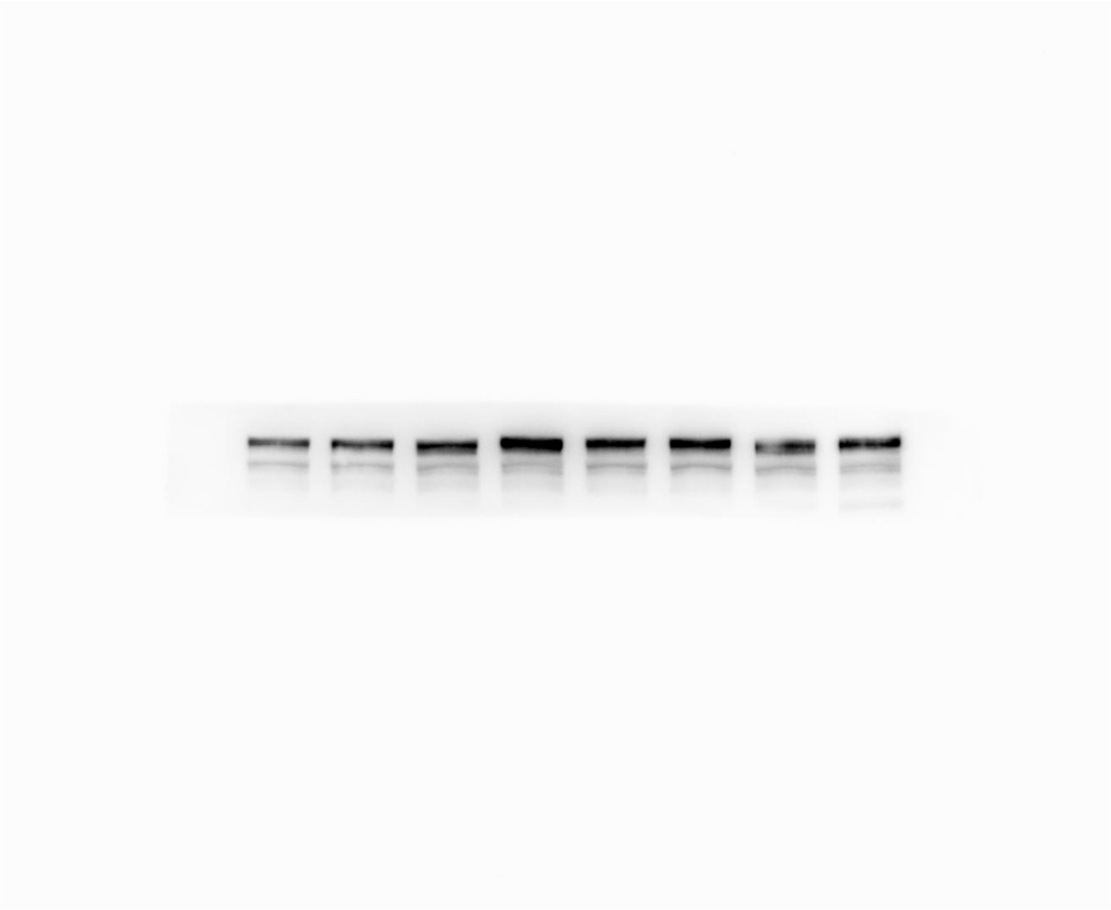

36.fig.5J-3-zo-right

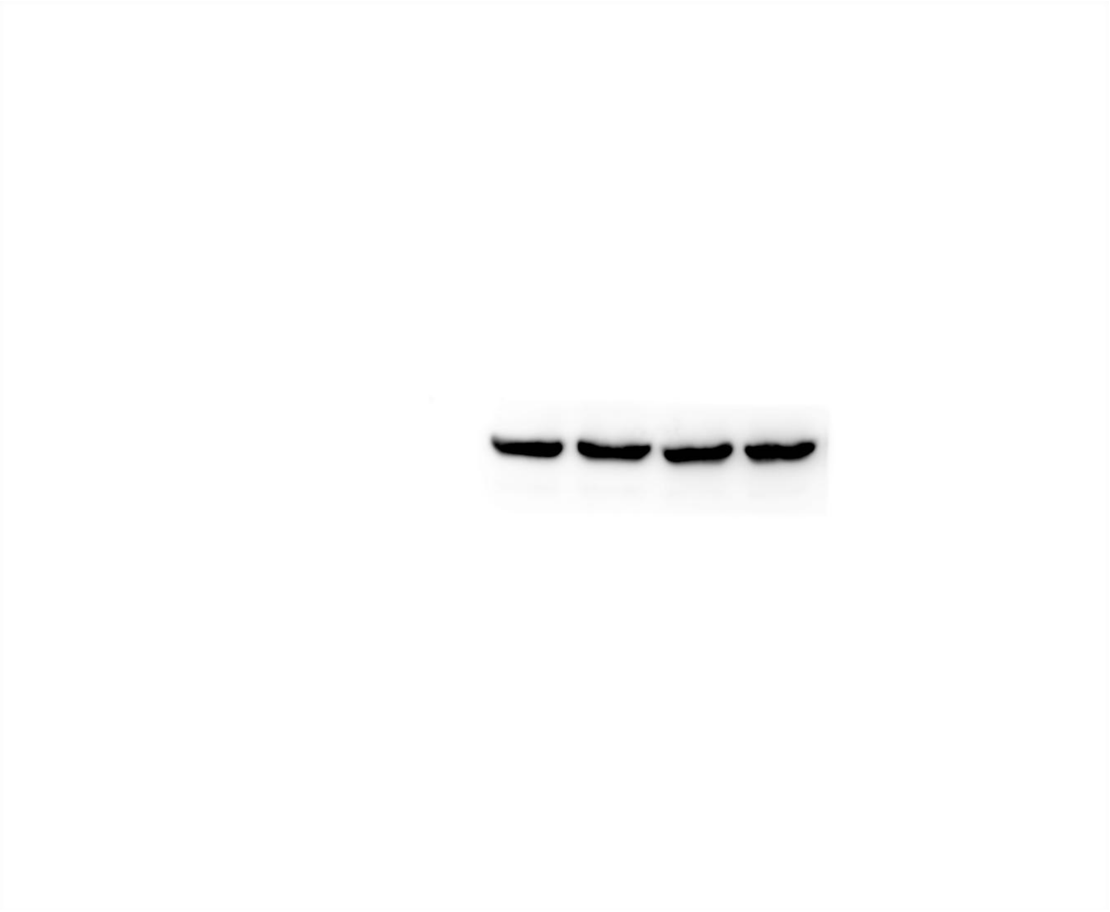

37.fig.6A-1-gap

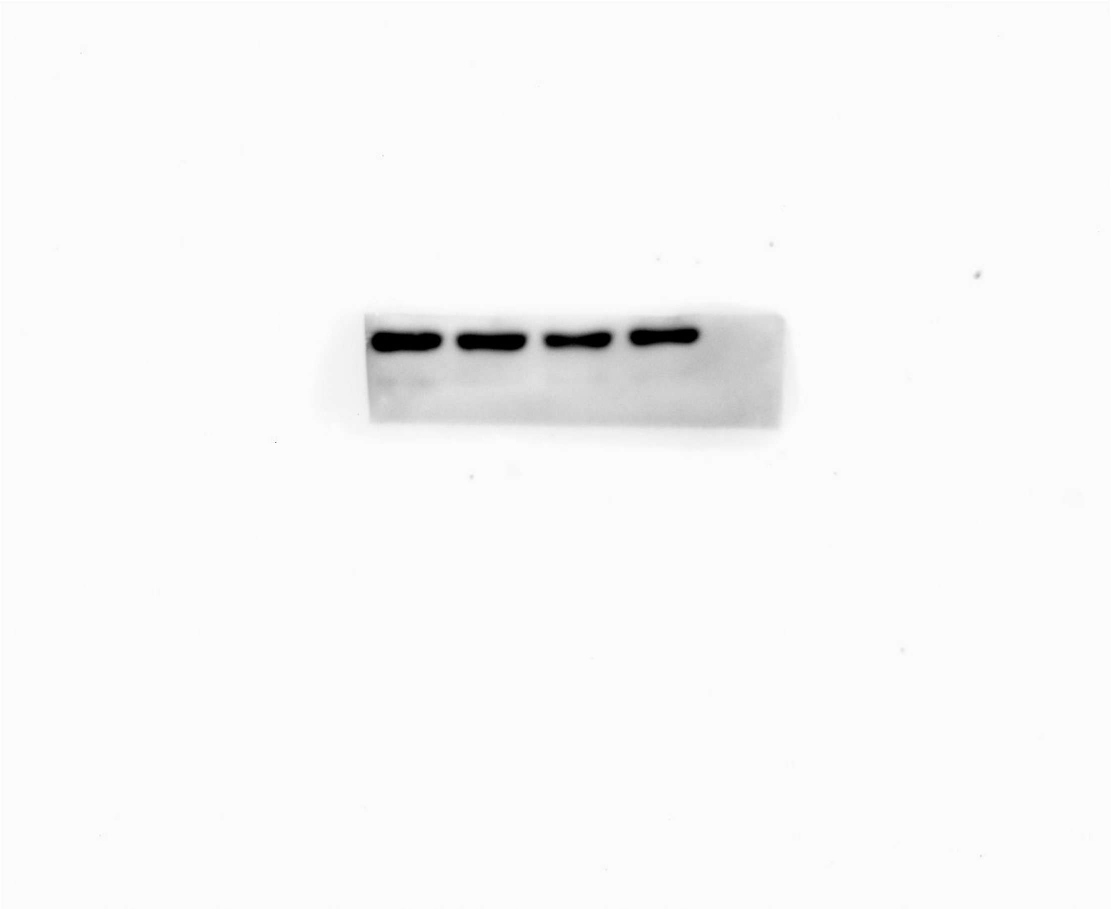

38.fig.6A-2-gap

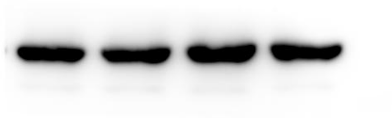

39.fig.6A-3-gap

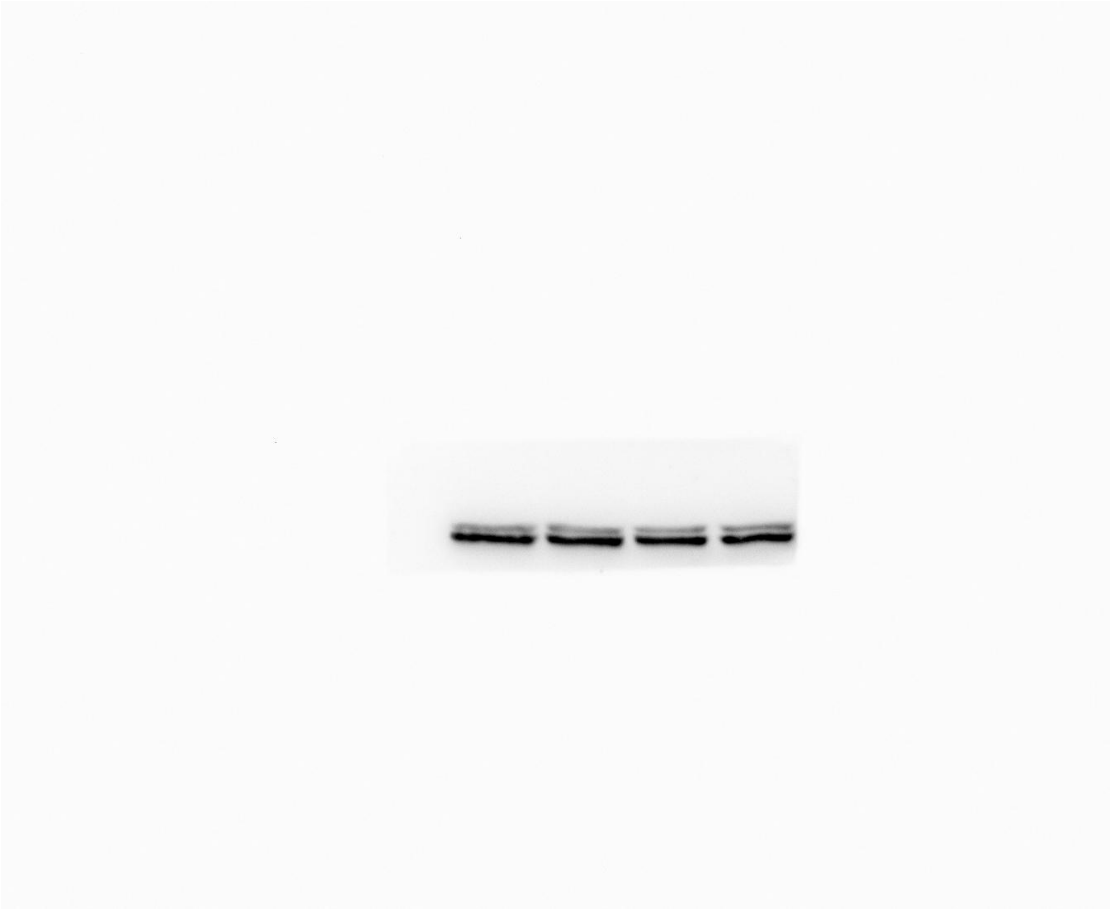

40.fig.6A-1-erk

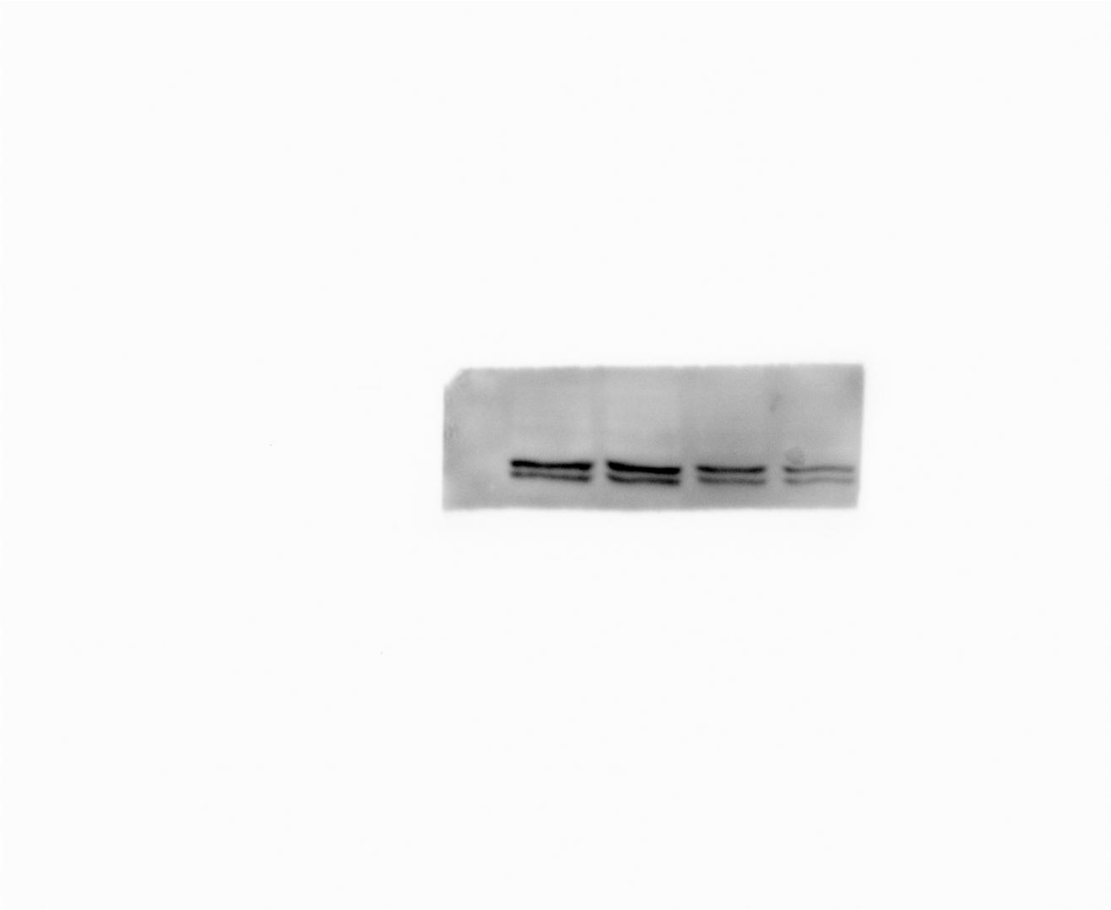

41.fig.6A-1-p-erk

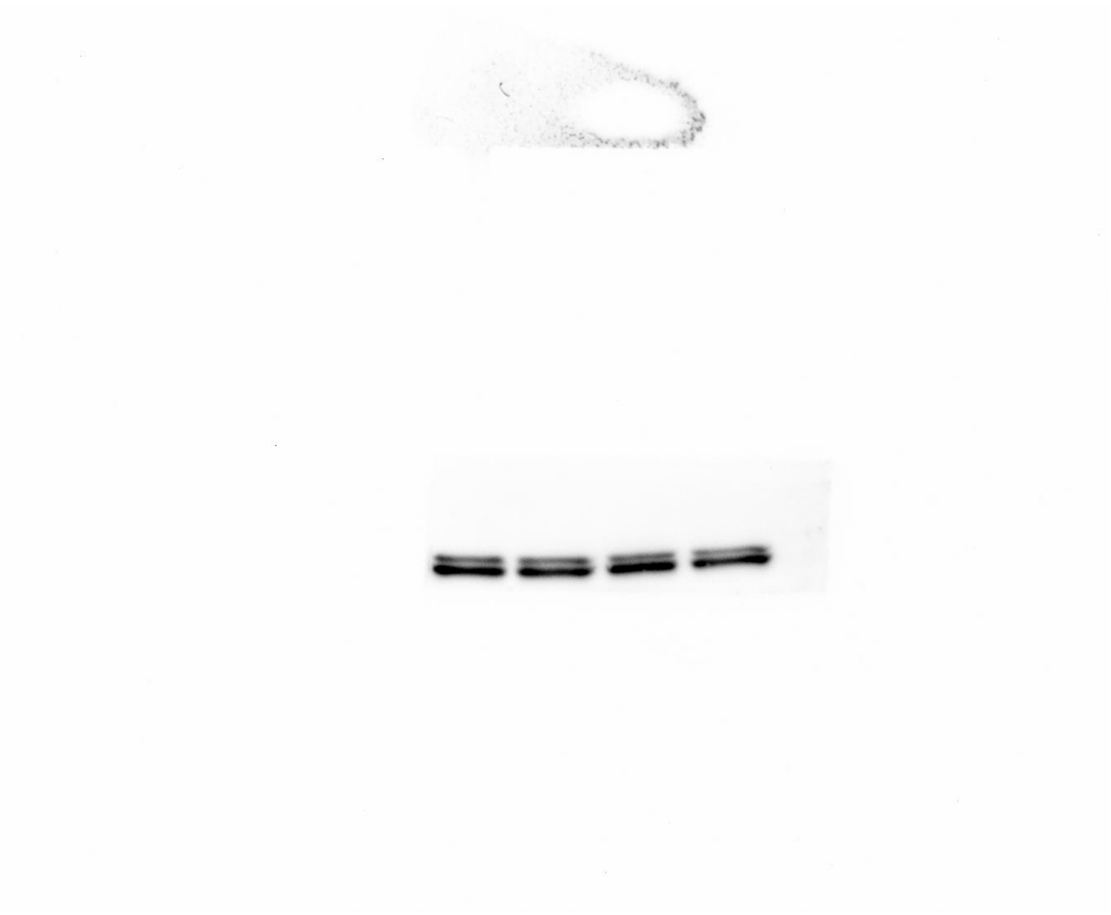

42.fig.6A-2-erk

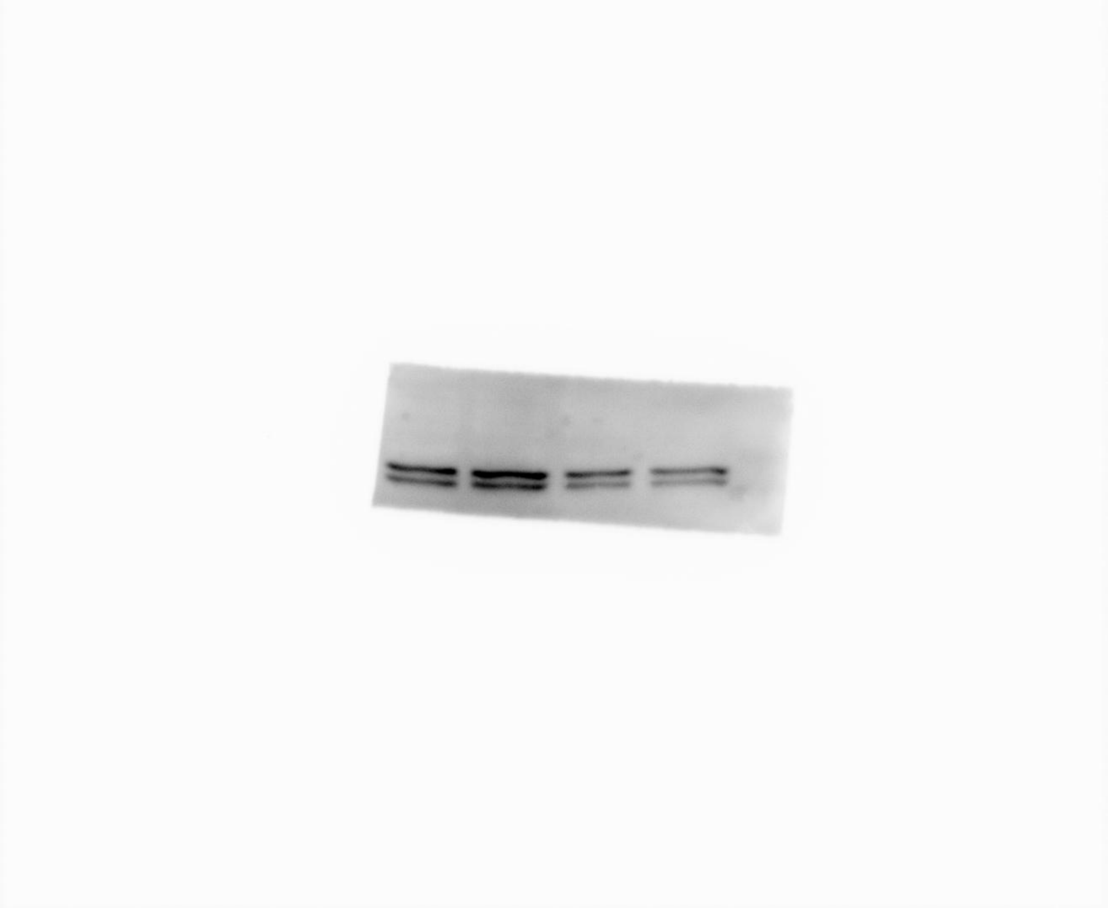

43.fig.6A-2-p-erk

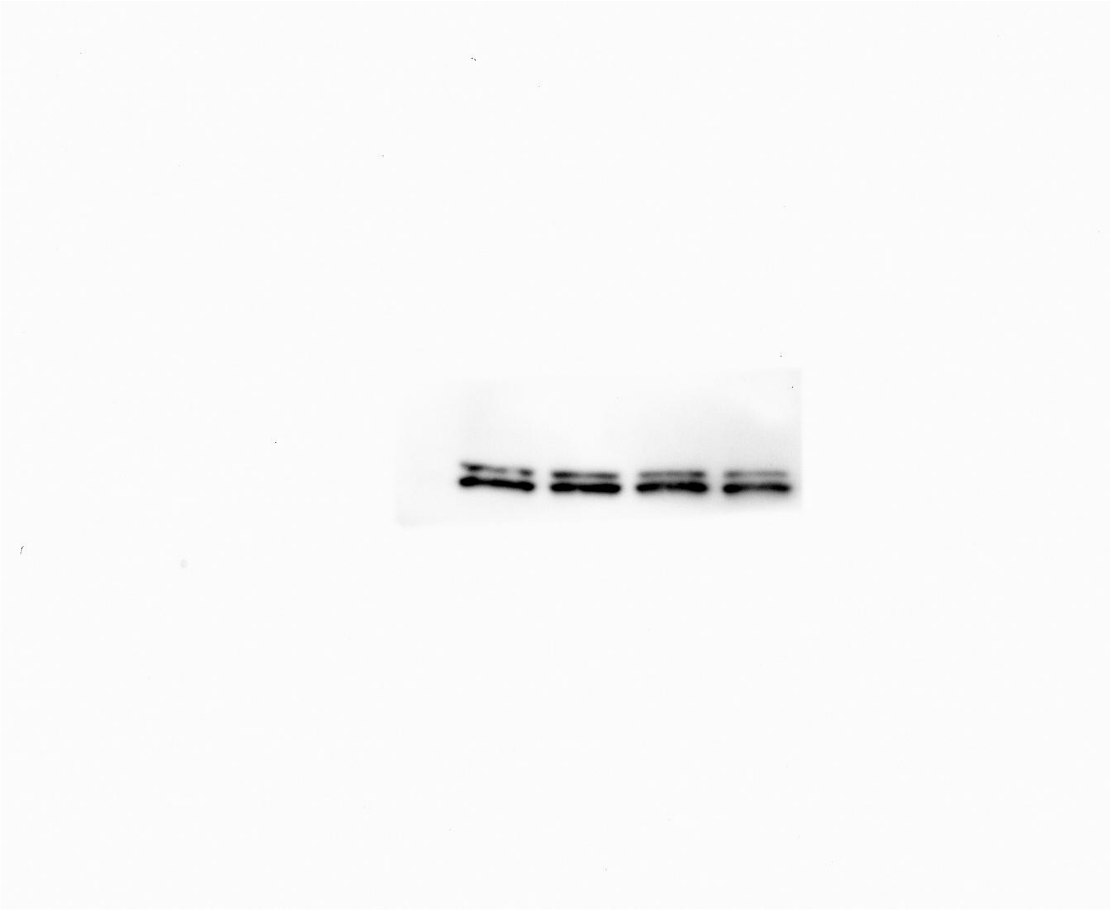

44.fig.6A-3-erk

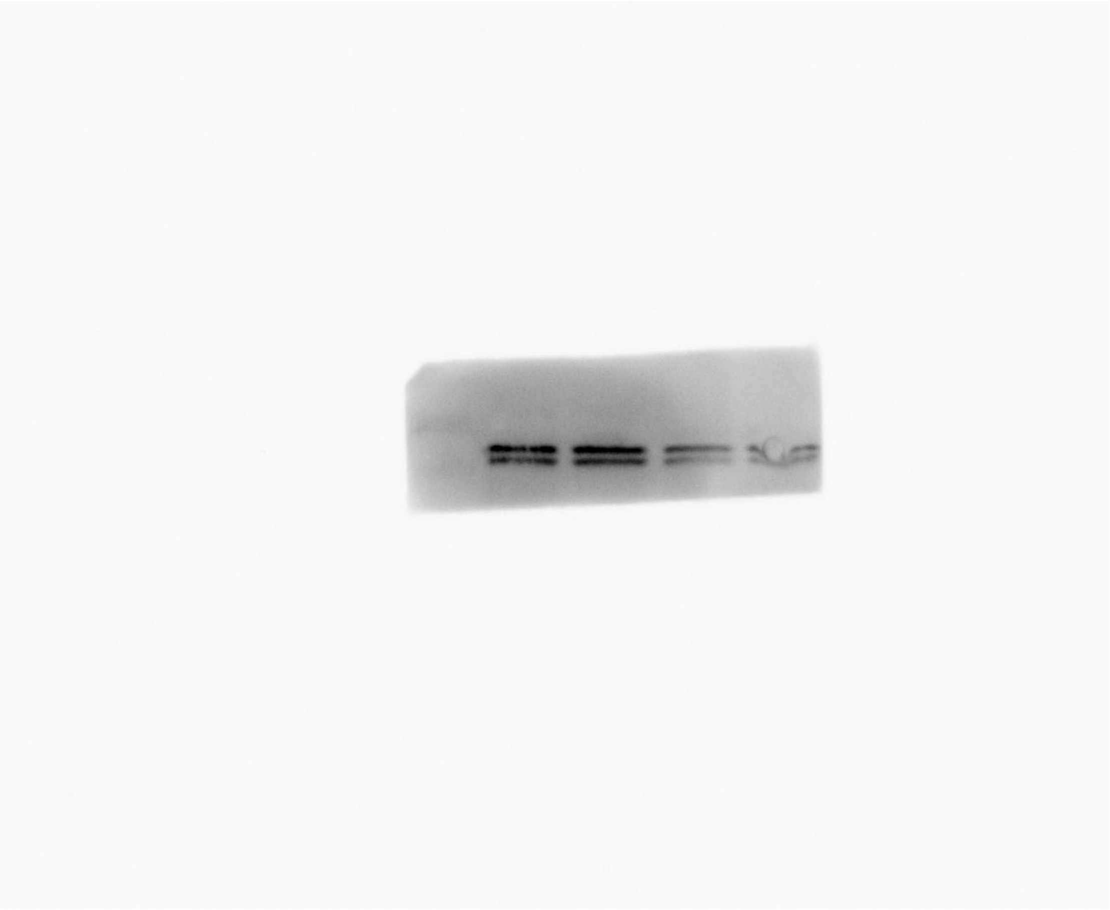

45.fig.6A-3-p-erk

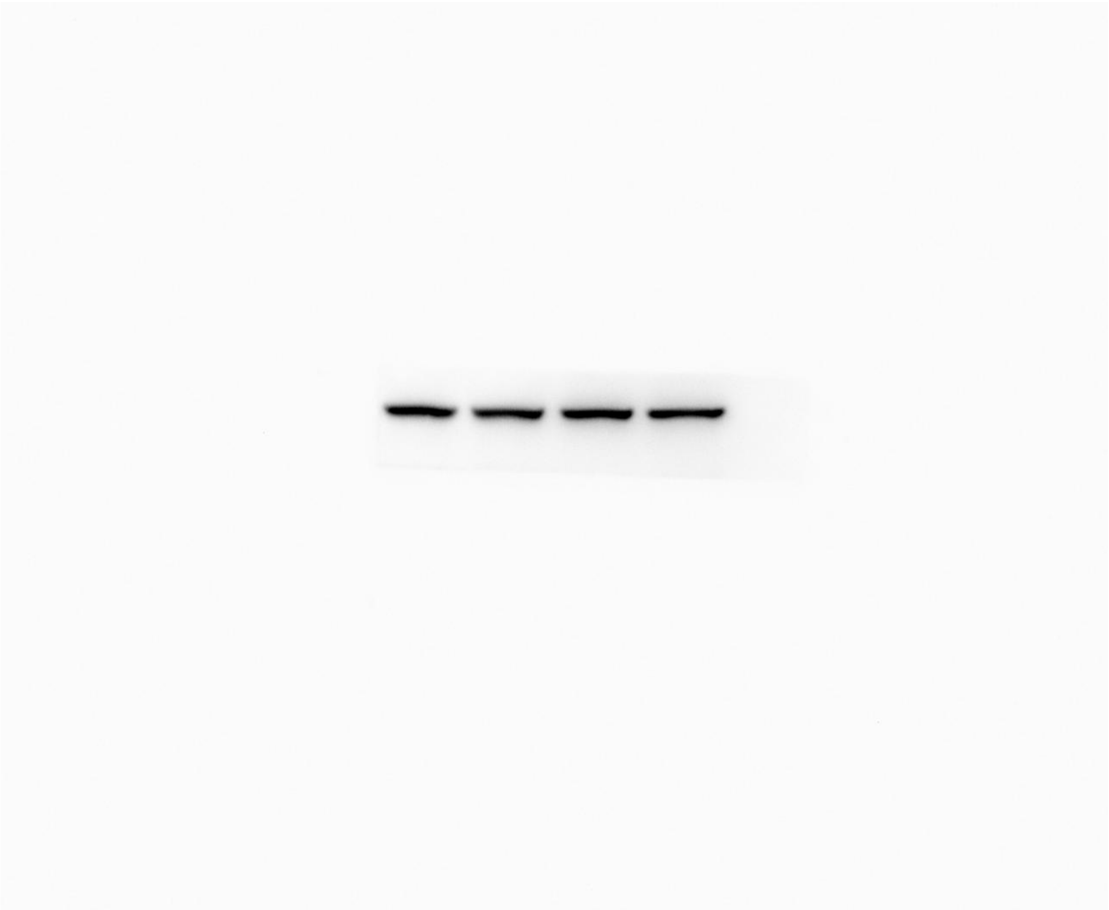

46.fig.6A-1-mek

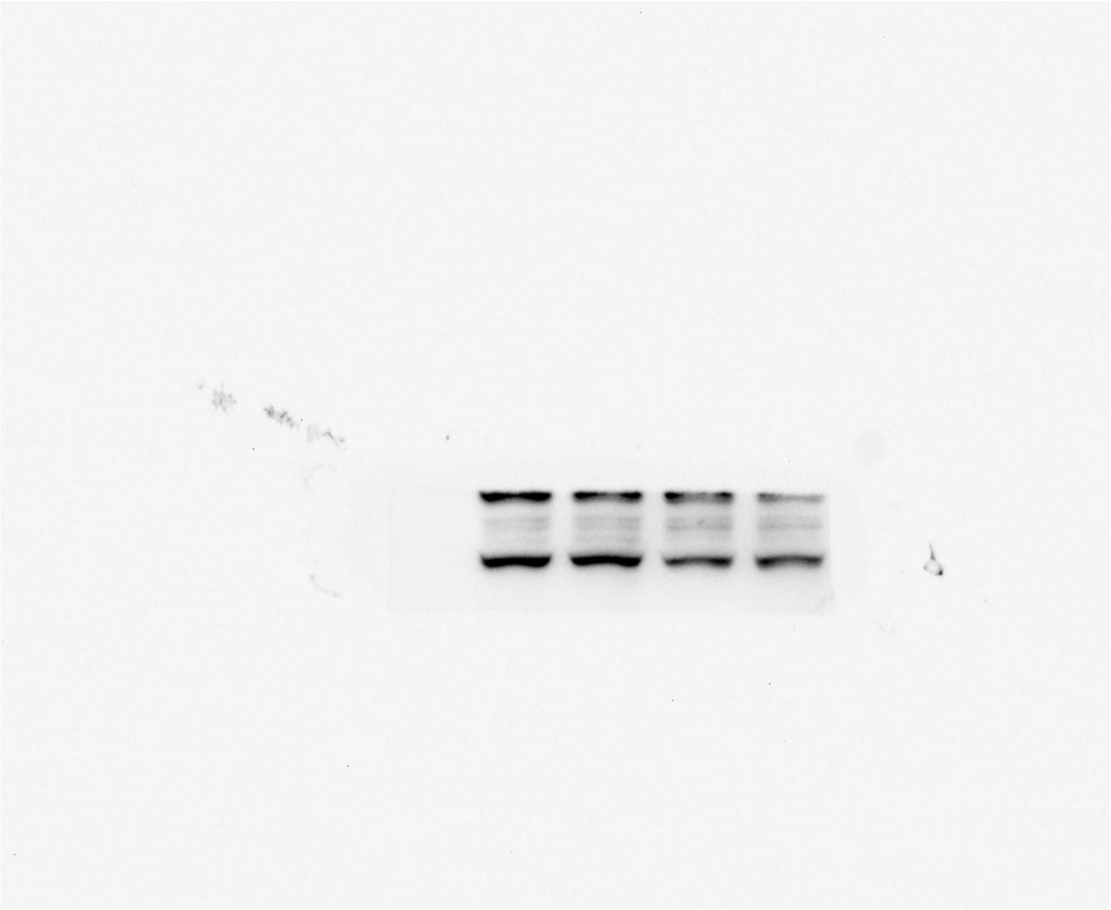

47.fig.6A-1-p-mek

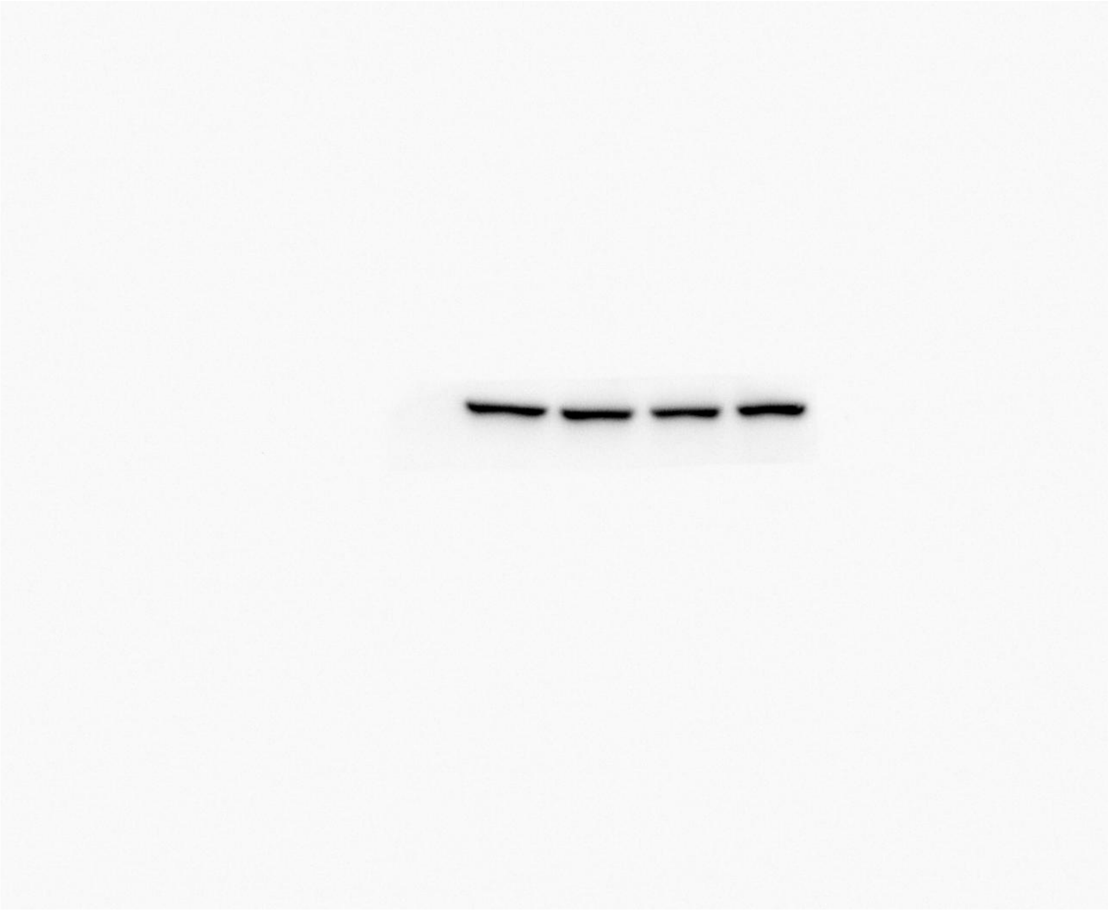

48.fig.6A-2-mek

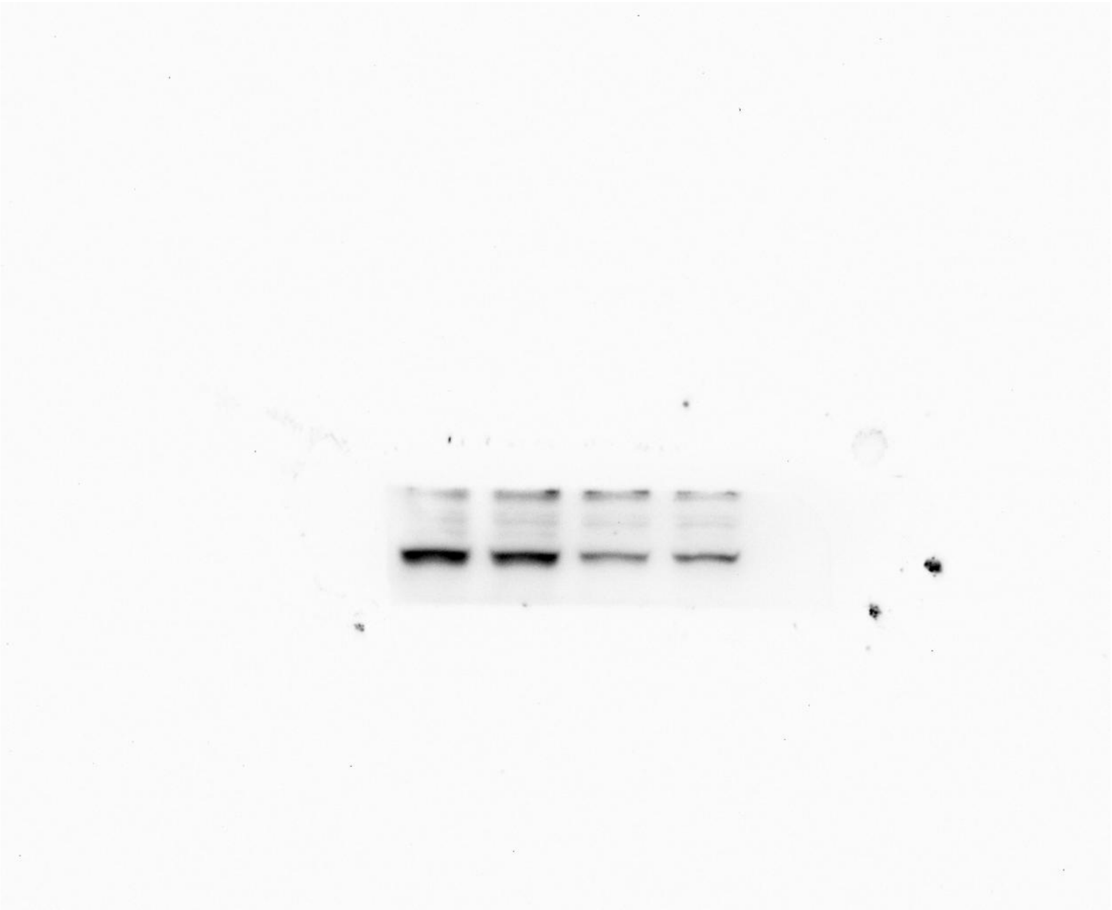

49.fig.6A-2-p-mek

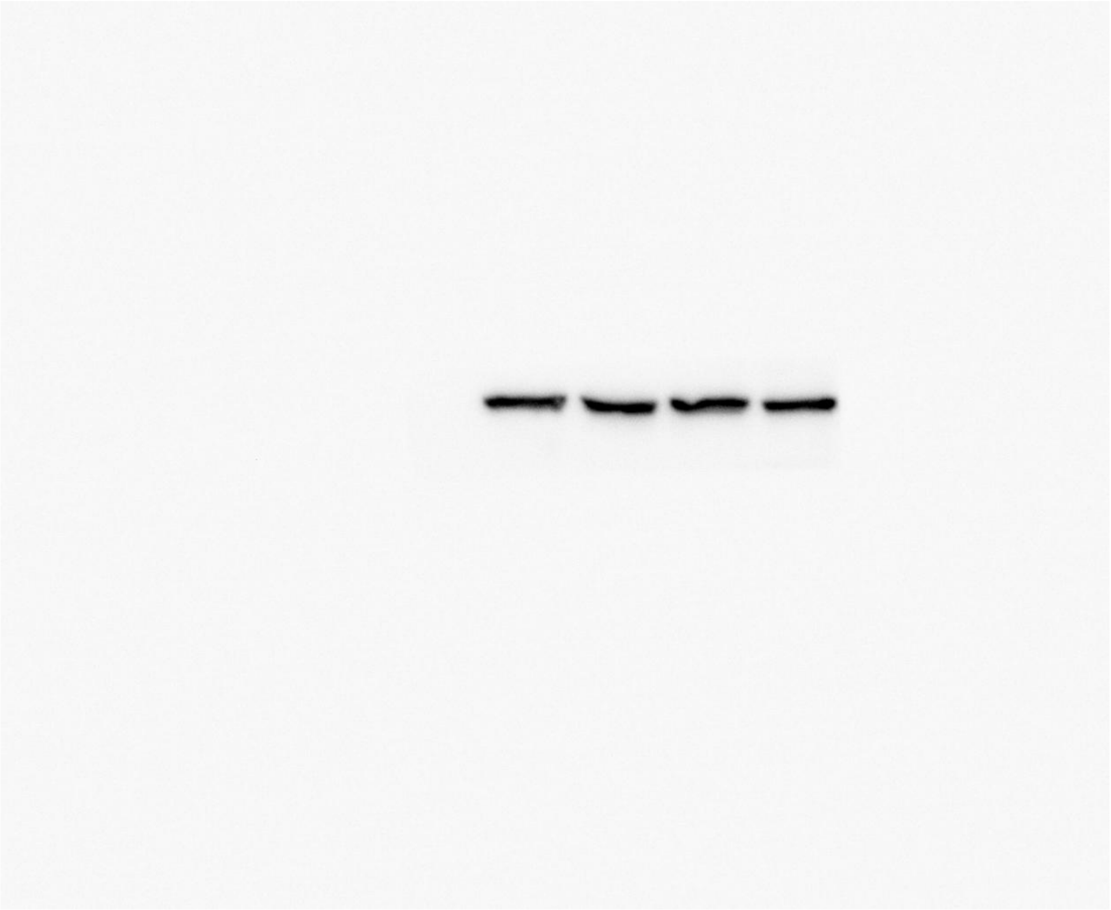

50.fig.6A-3-mek

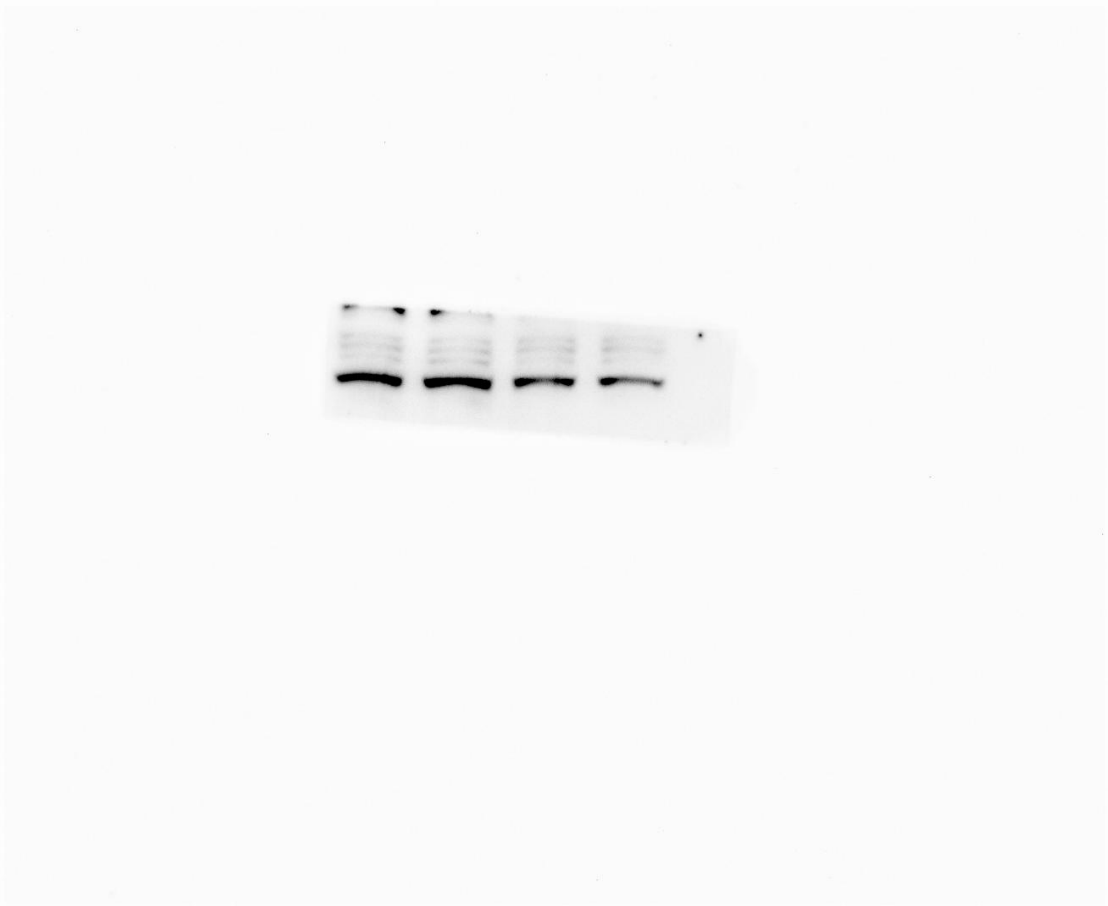

51.fig.6A-3-p-mek

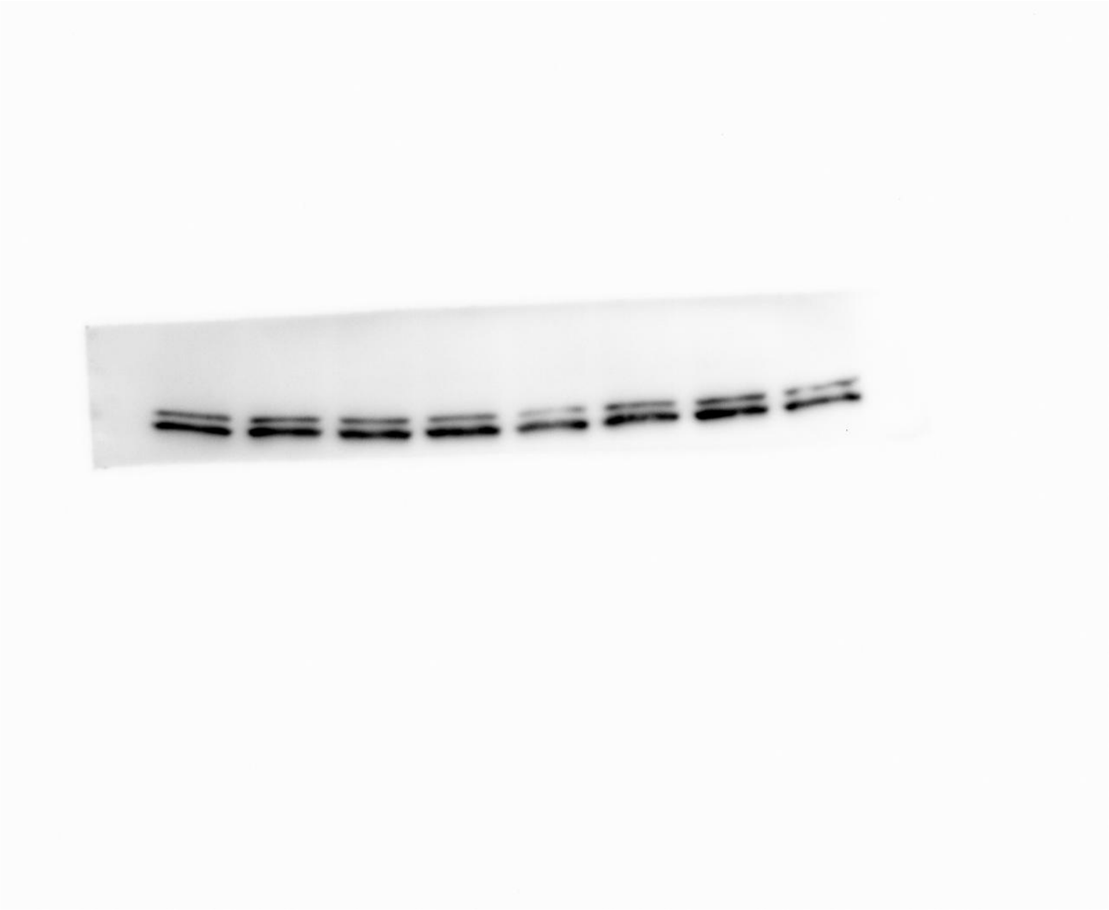

52.fig.6B-1-erk-left

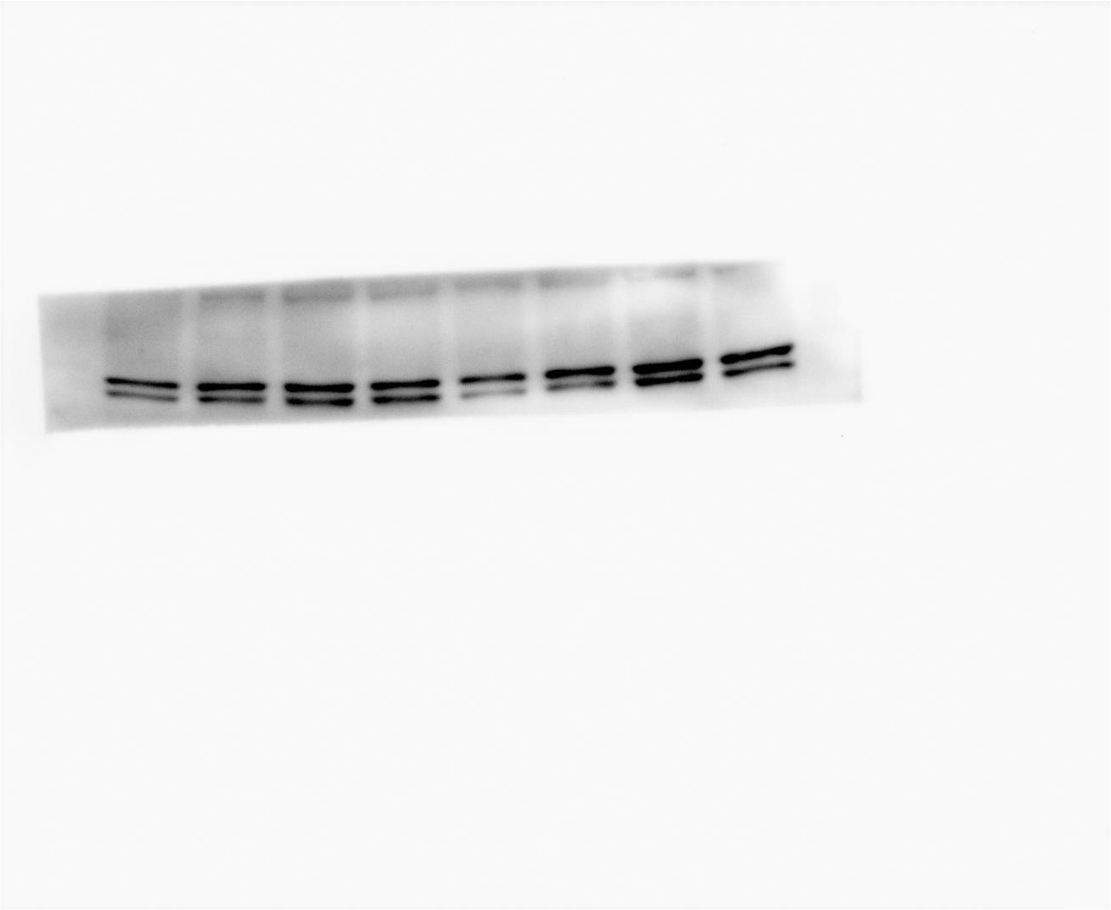

53.fig.6B-1-p-erk-left

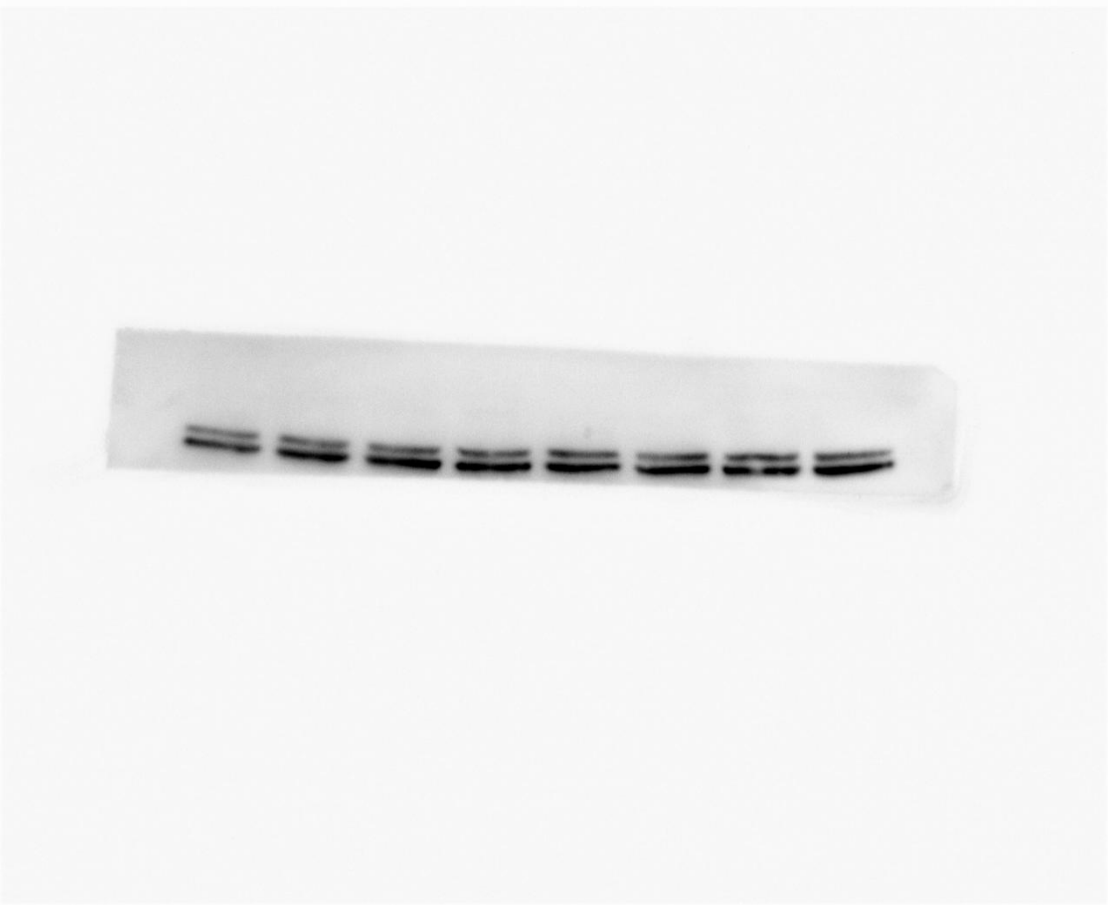

54.fig.6B-2-erk-right

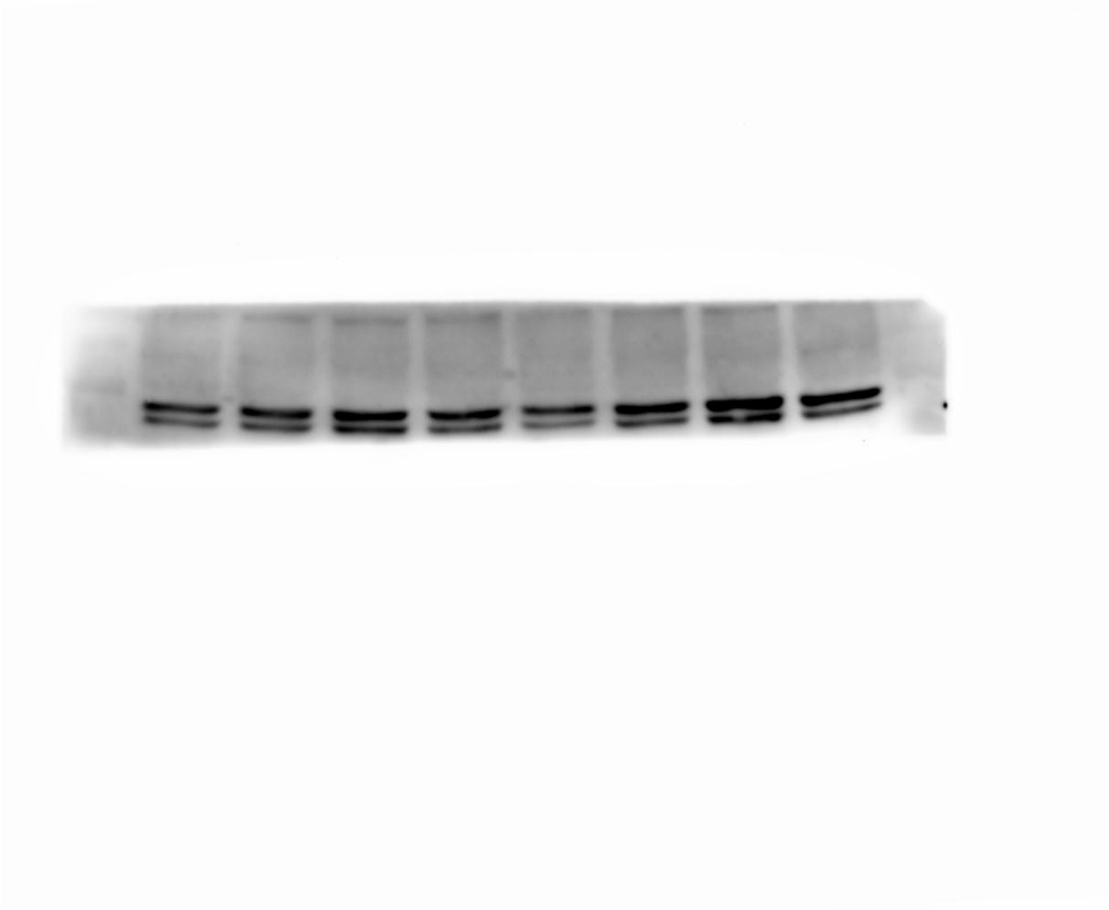

55.fig.6B-2-p-erk-right

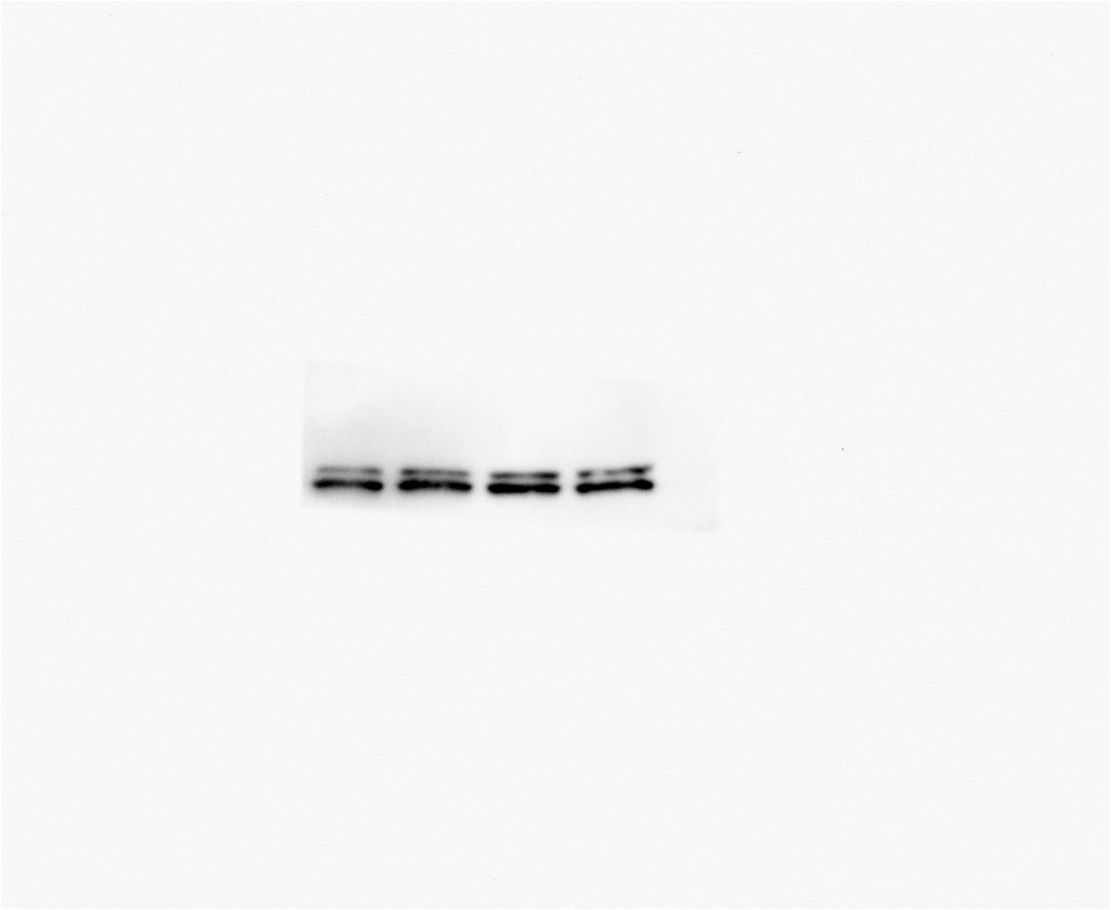

56.fig.6B-3-erk

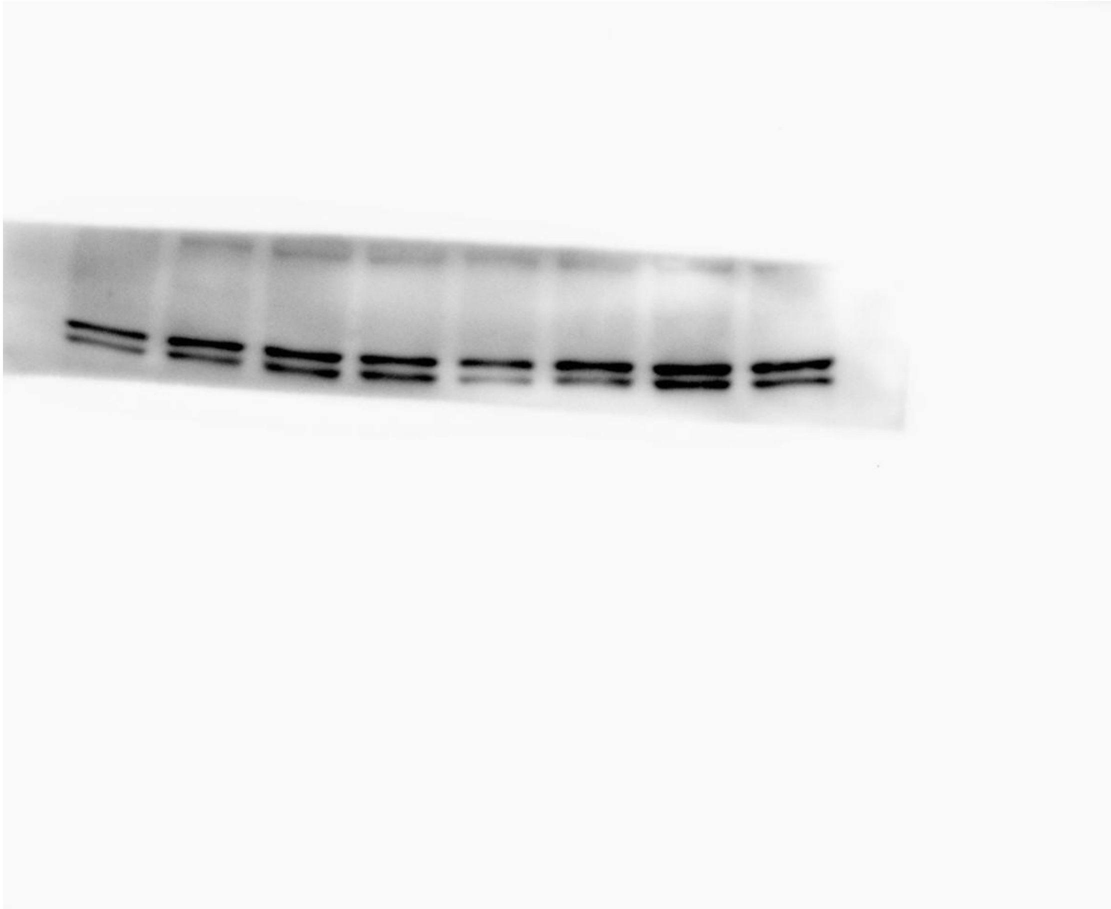

57.fig.6B-3-p-erk-right

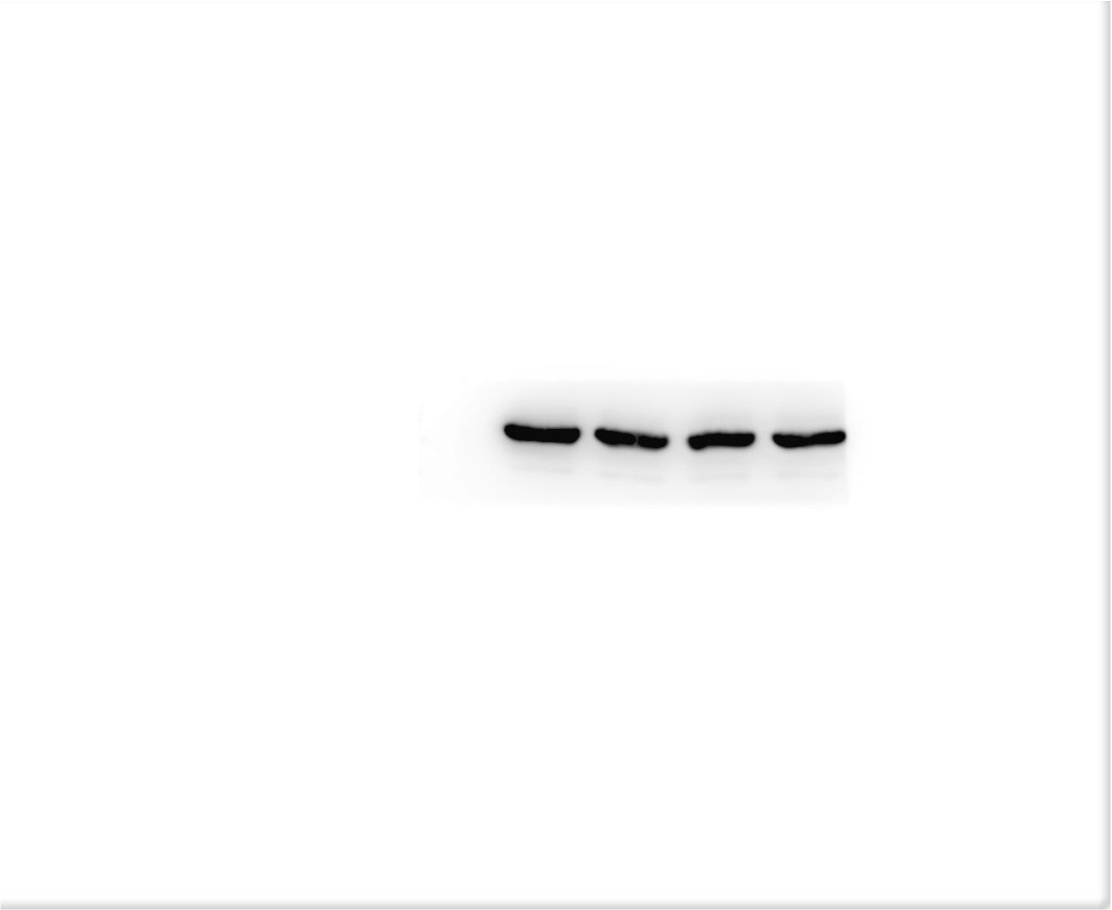

58.fig.6B-1-gap

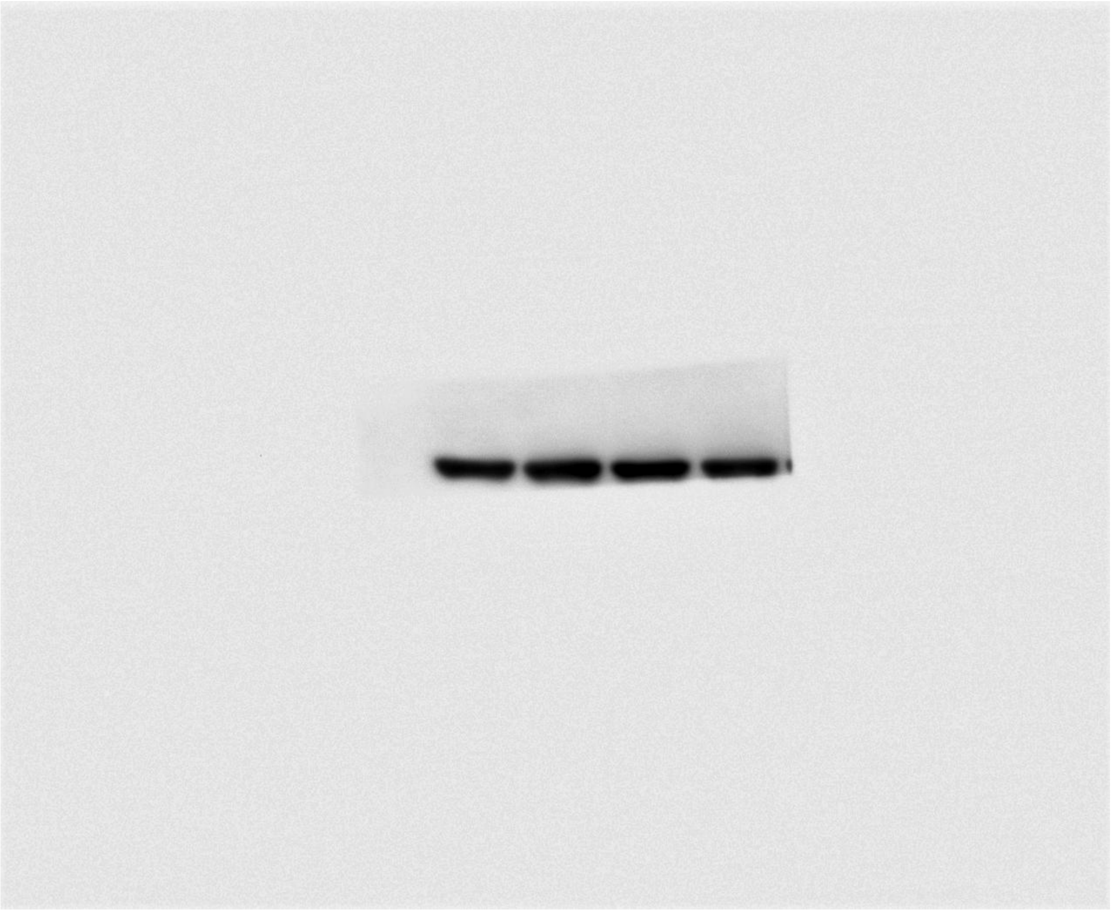

59.fig.6B-2-gap

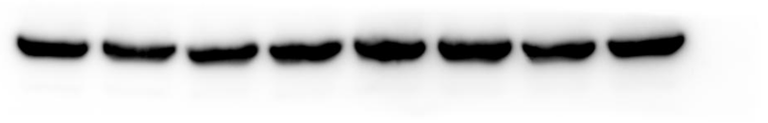

60.fig.6B-3-gap-right

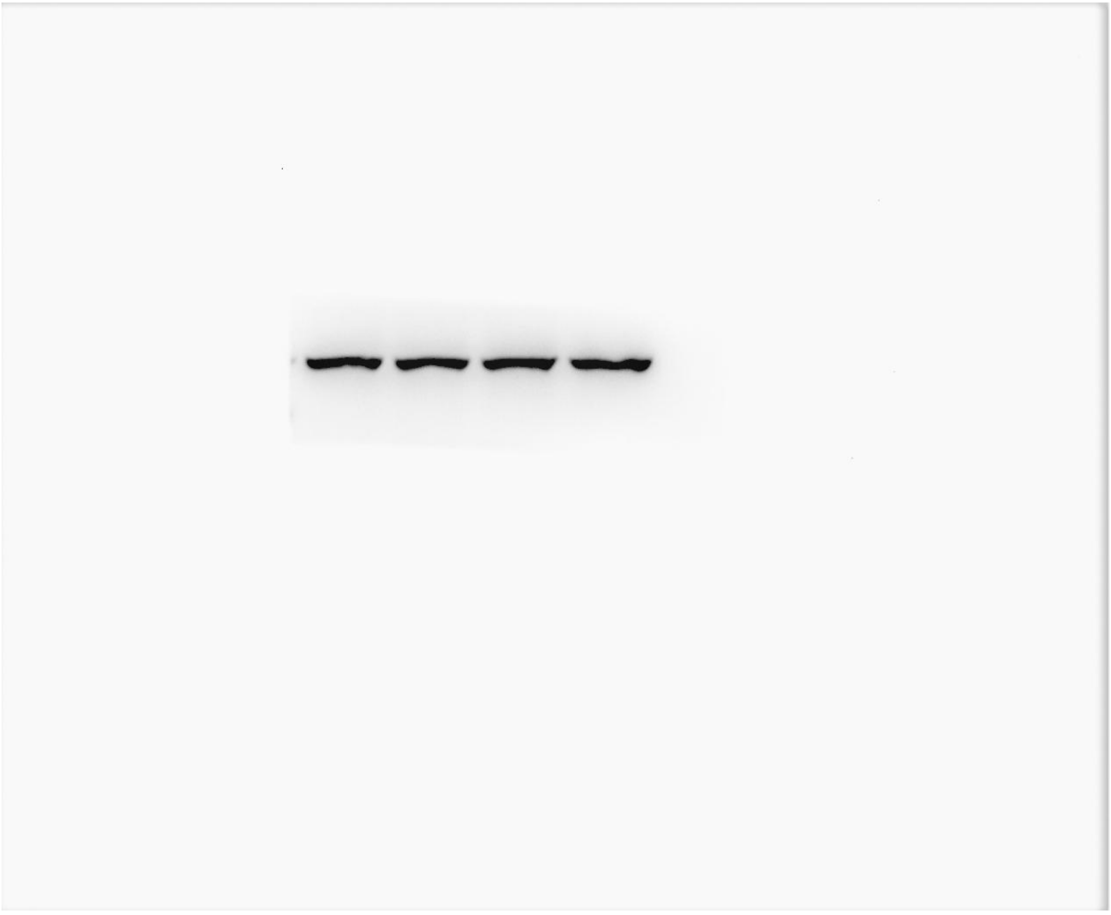

61.fig.6B-1-mek

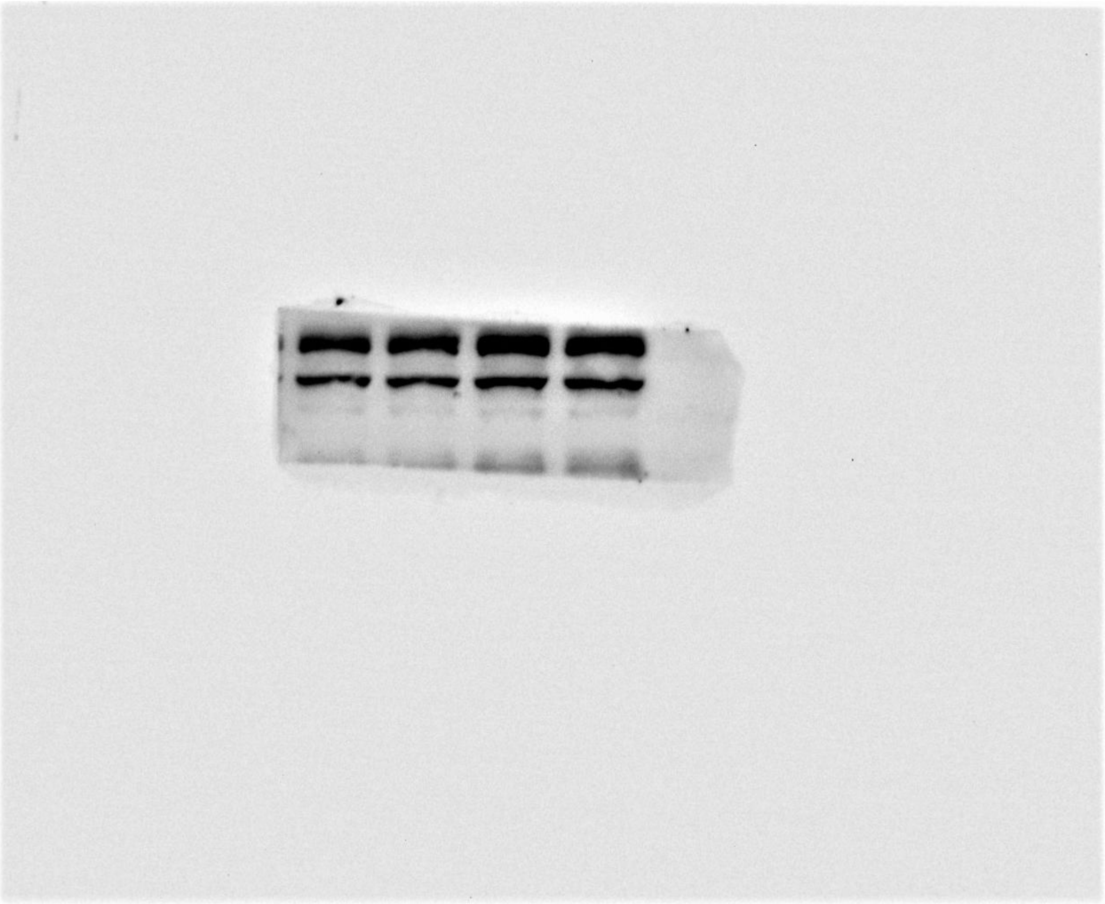

62.fig.6B-1-p-mek-down

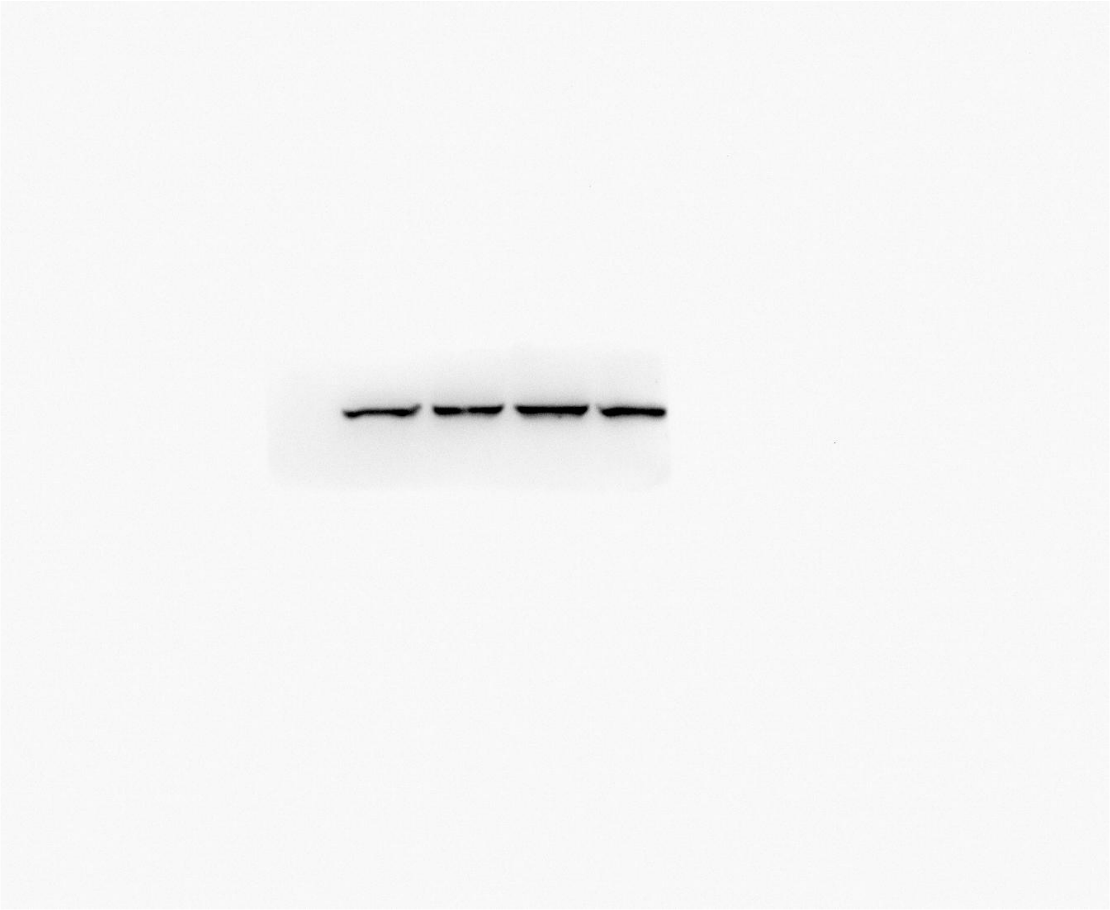

63.fig.6B-2-mek

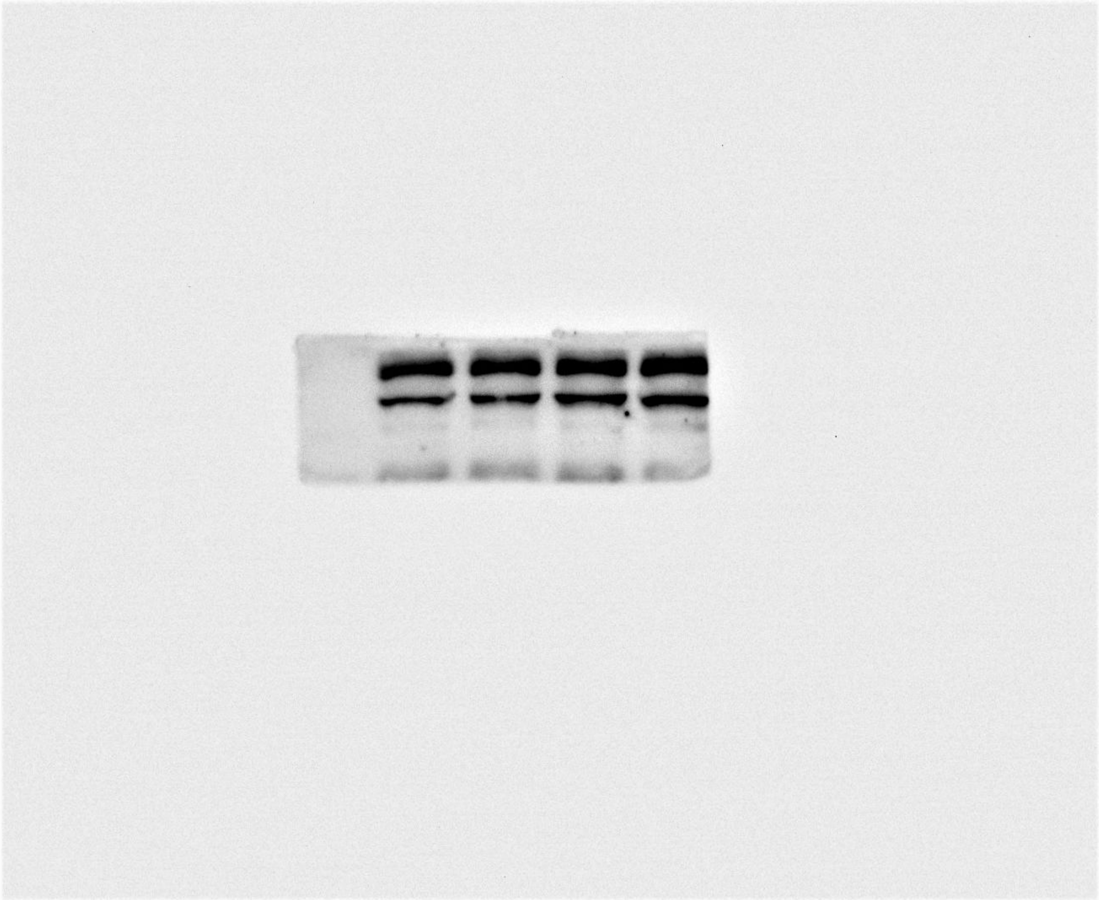

64.fig.6B-2-p-mek-down

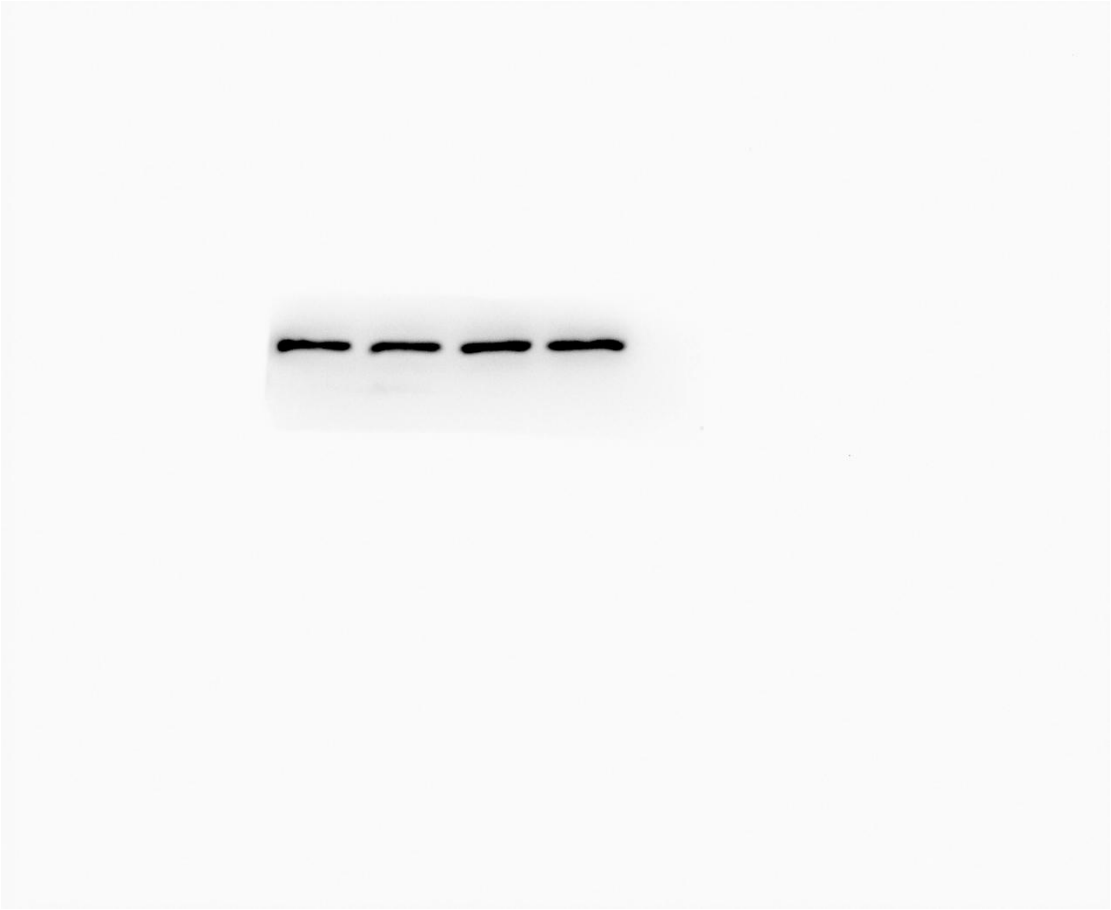

65.fig.6B-3-mek

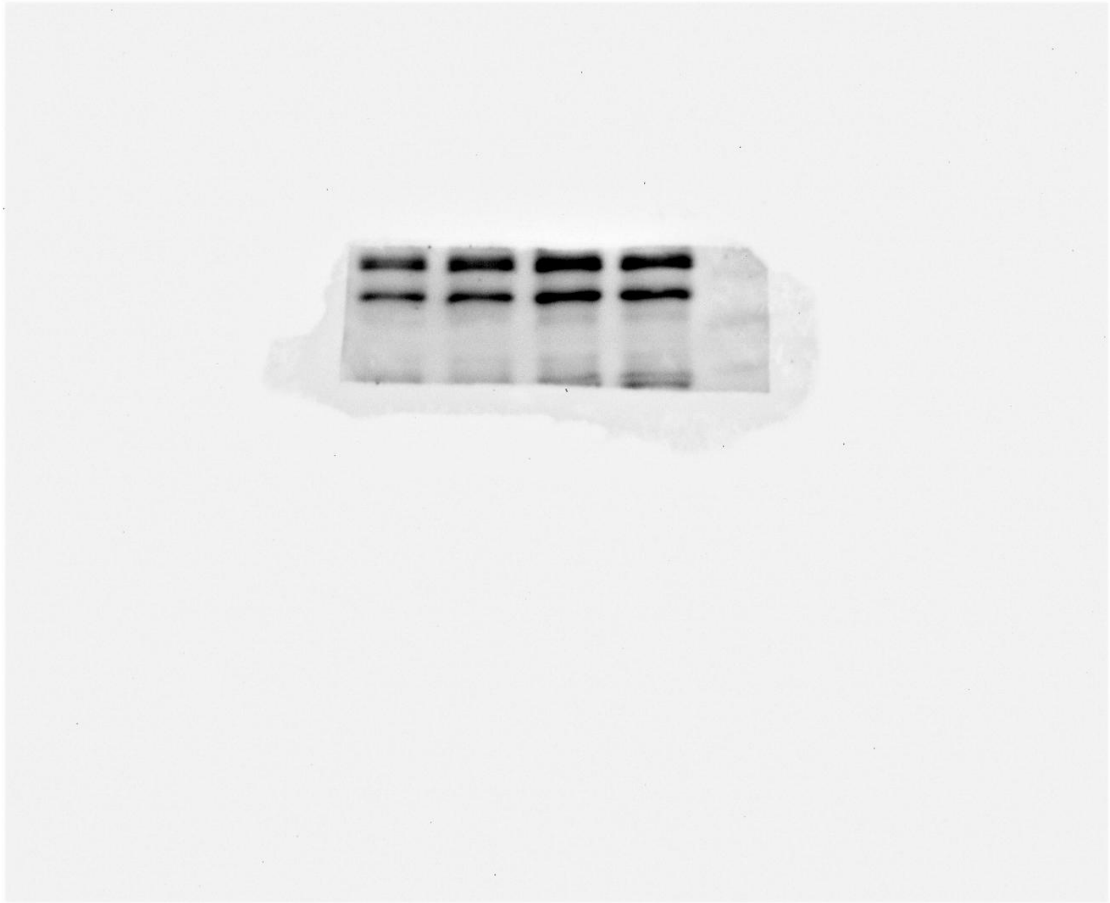

66.fig.6B-3-p-mek-down

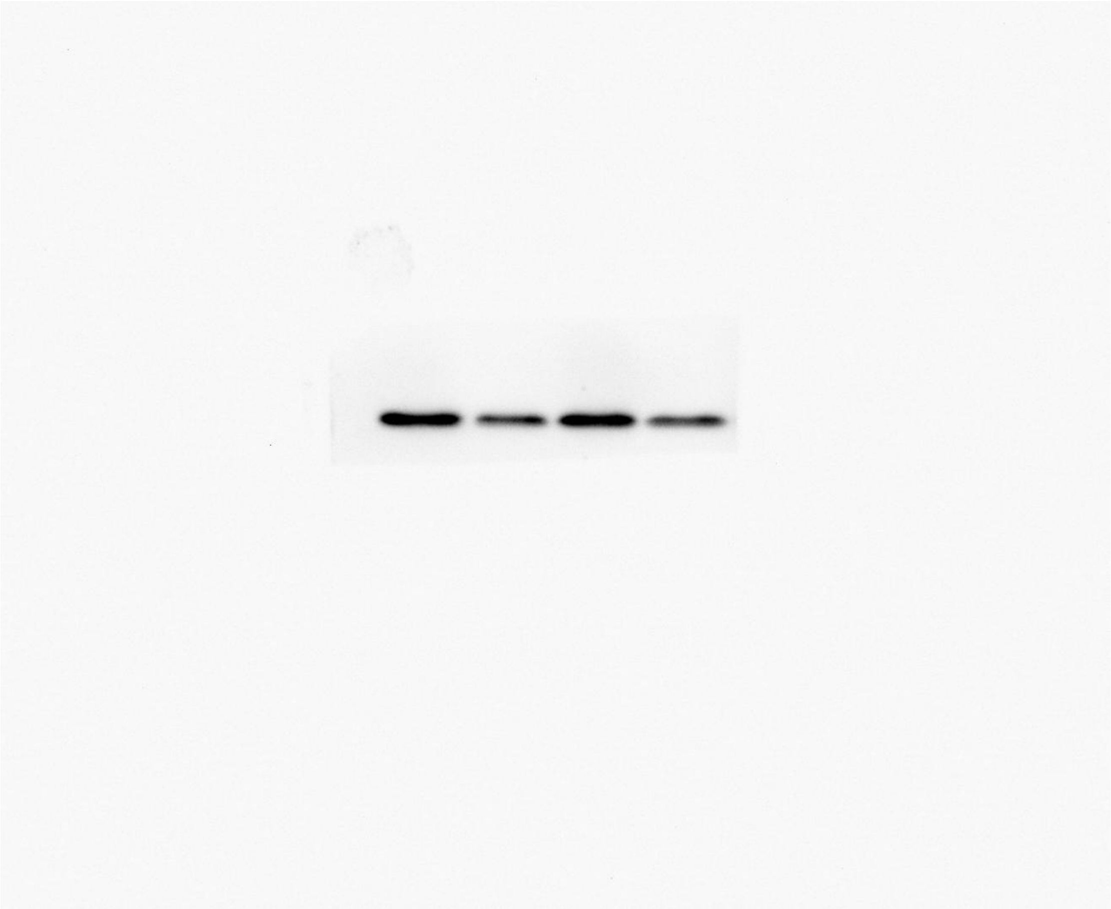

67.fig.7A-1-claudin

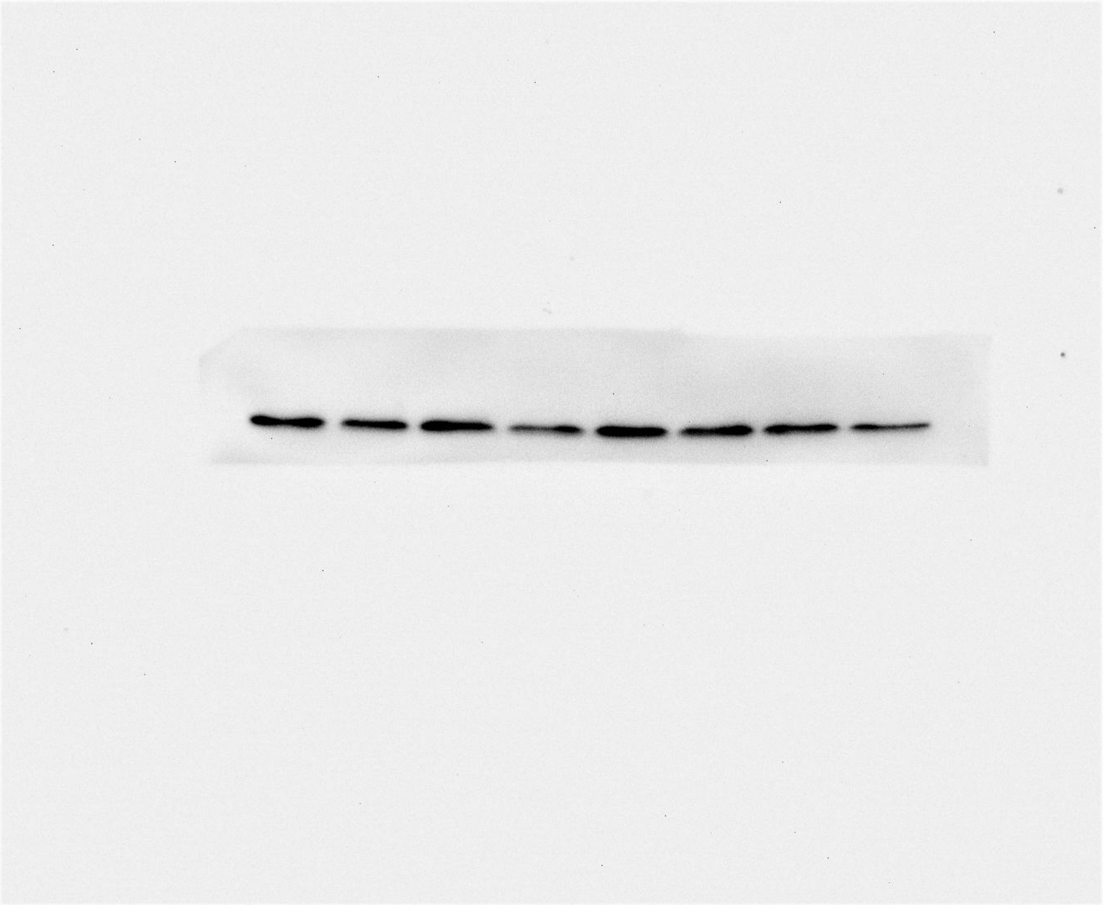

68.fig.7A-2-claudin-left

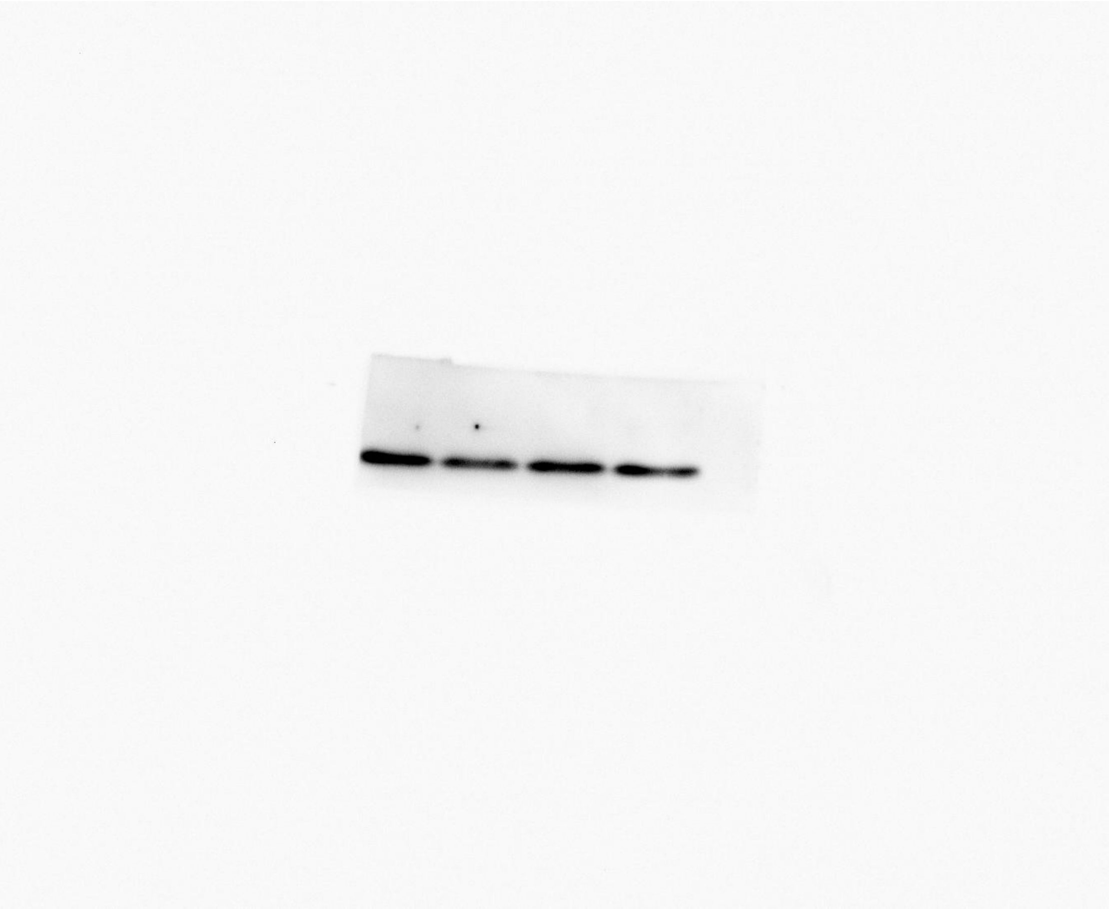

69.fig.7A-3-claudin

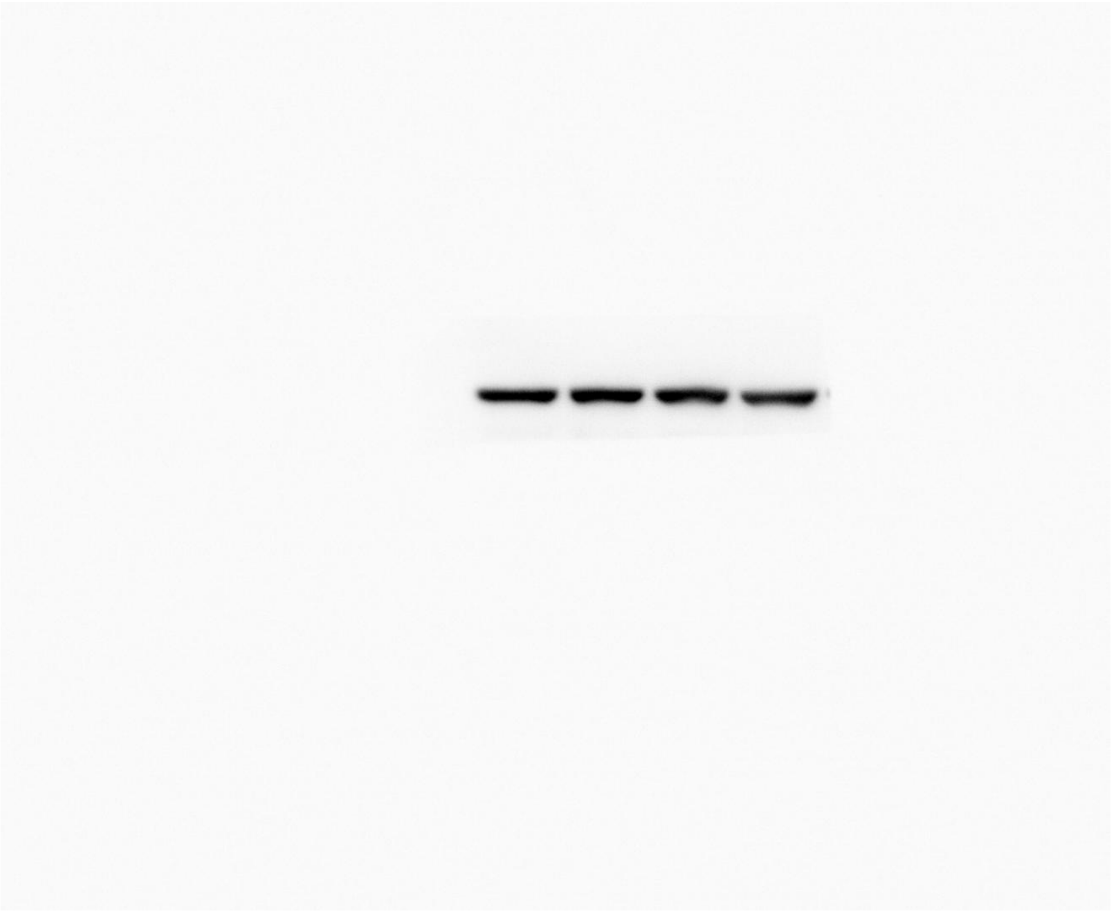

70.fig.7A-1-gap

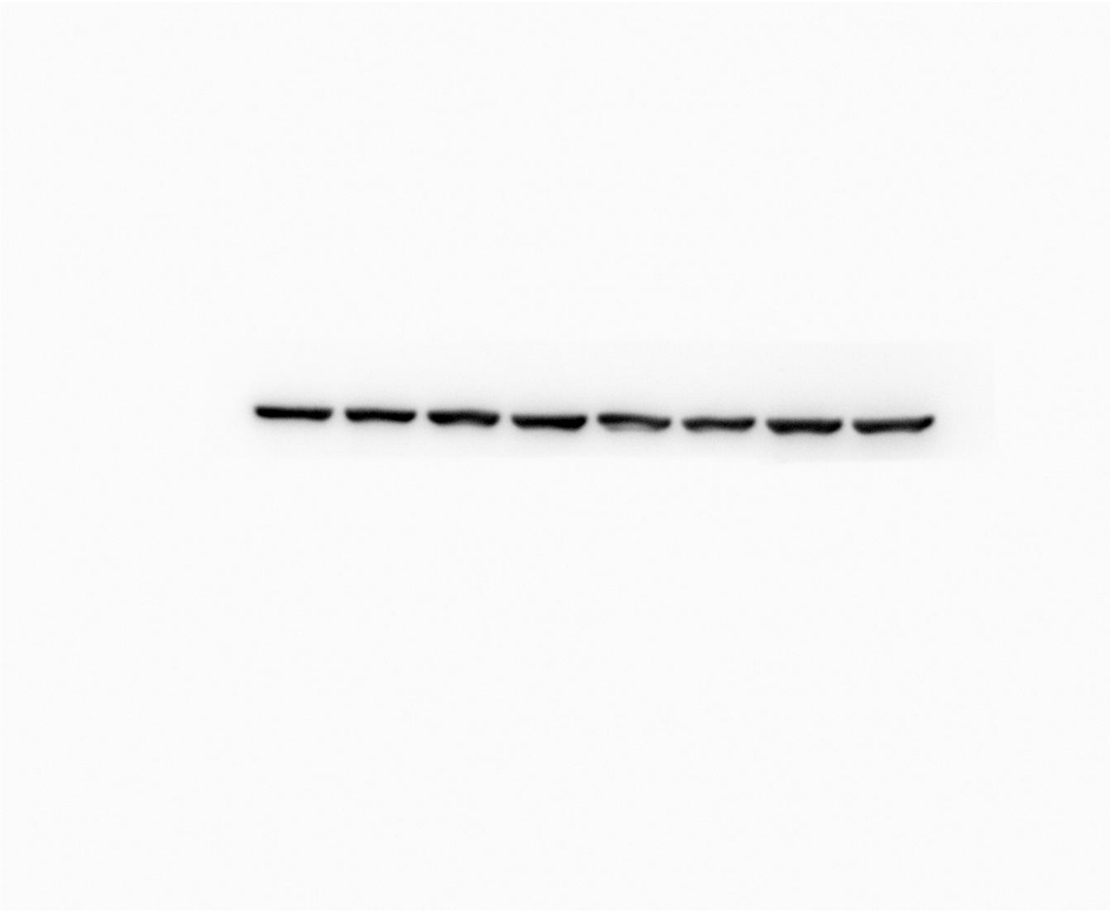

71.fig.7A-2-gap-right

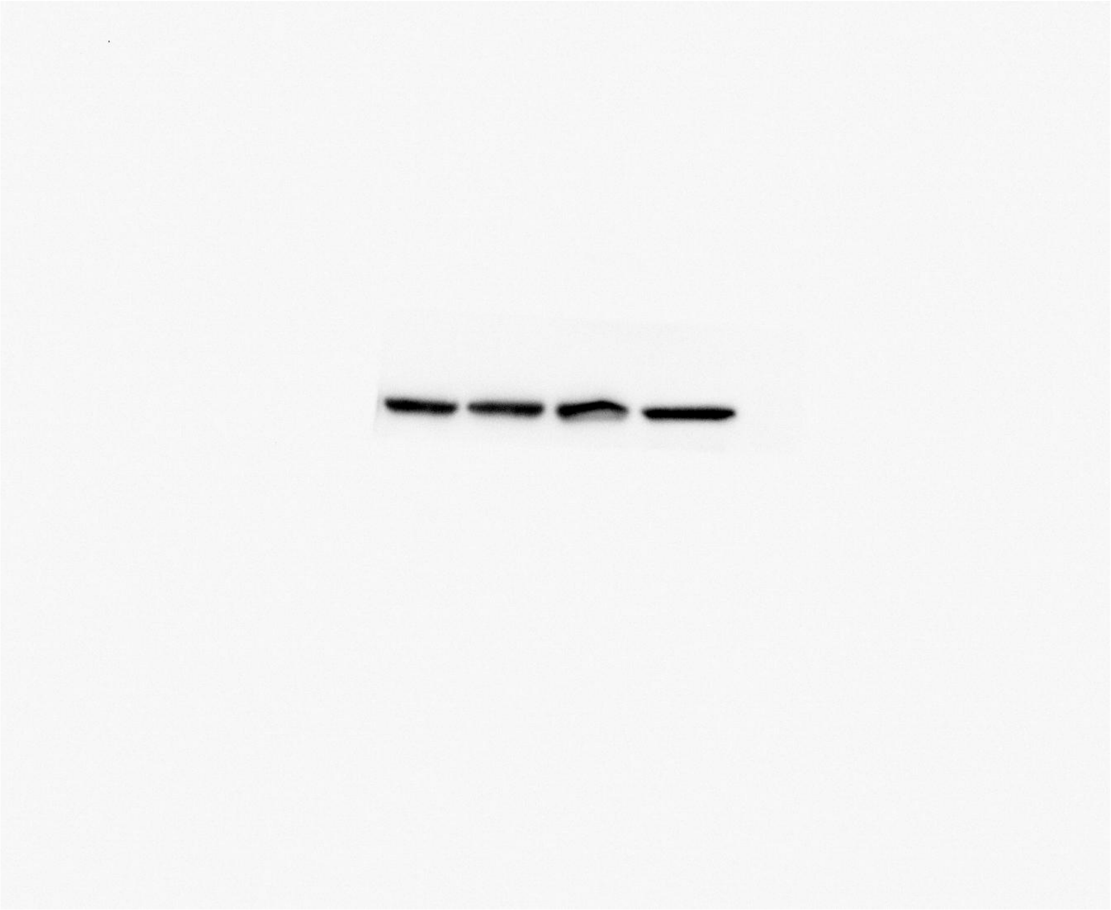

72.fig.7A-3-gap

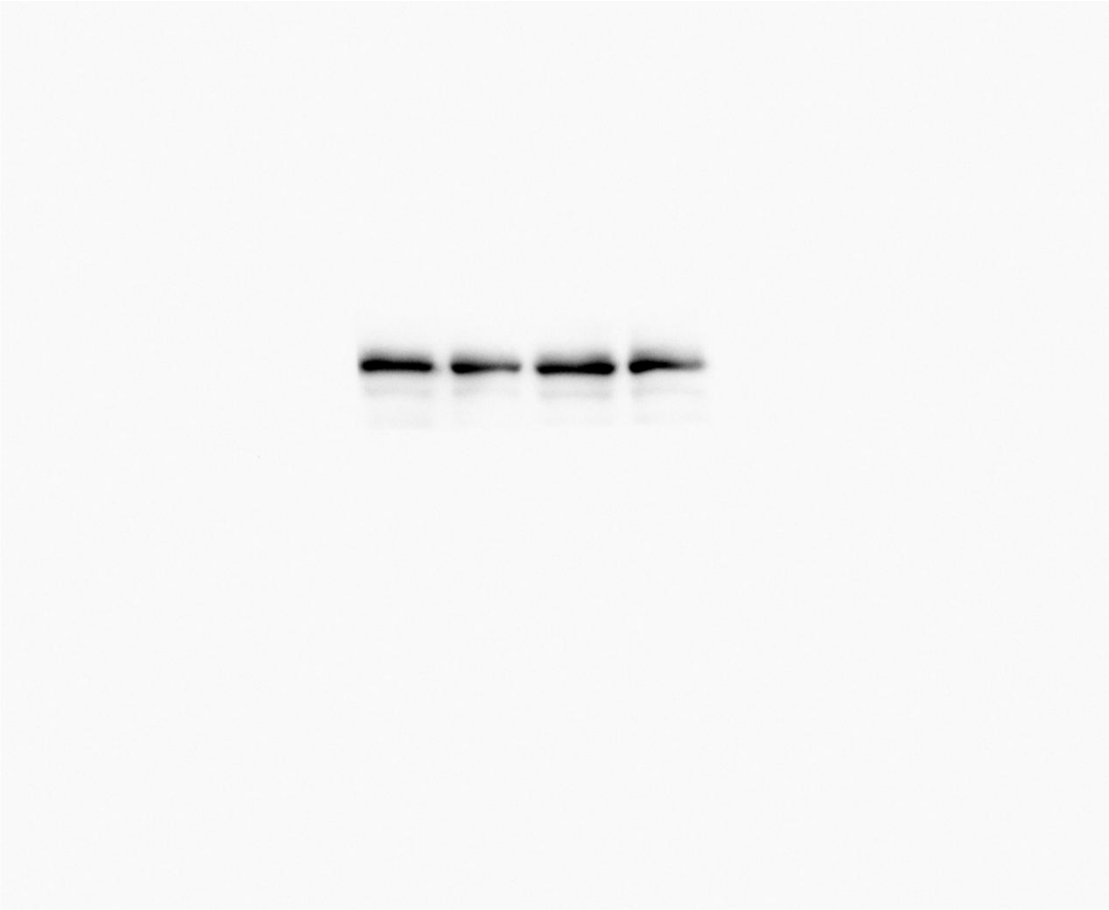

73.fig.7A-1-occludin

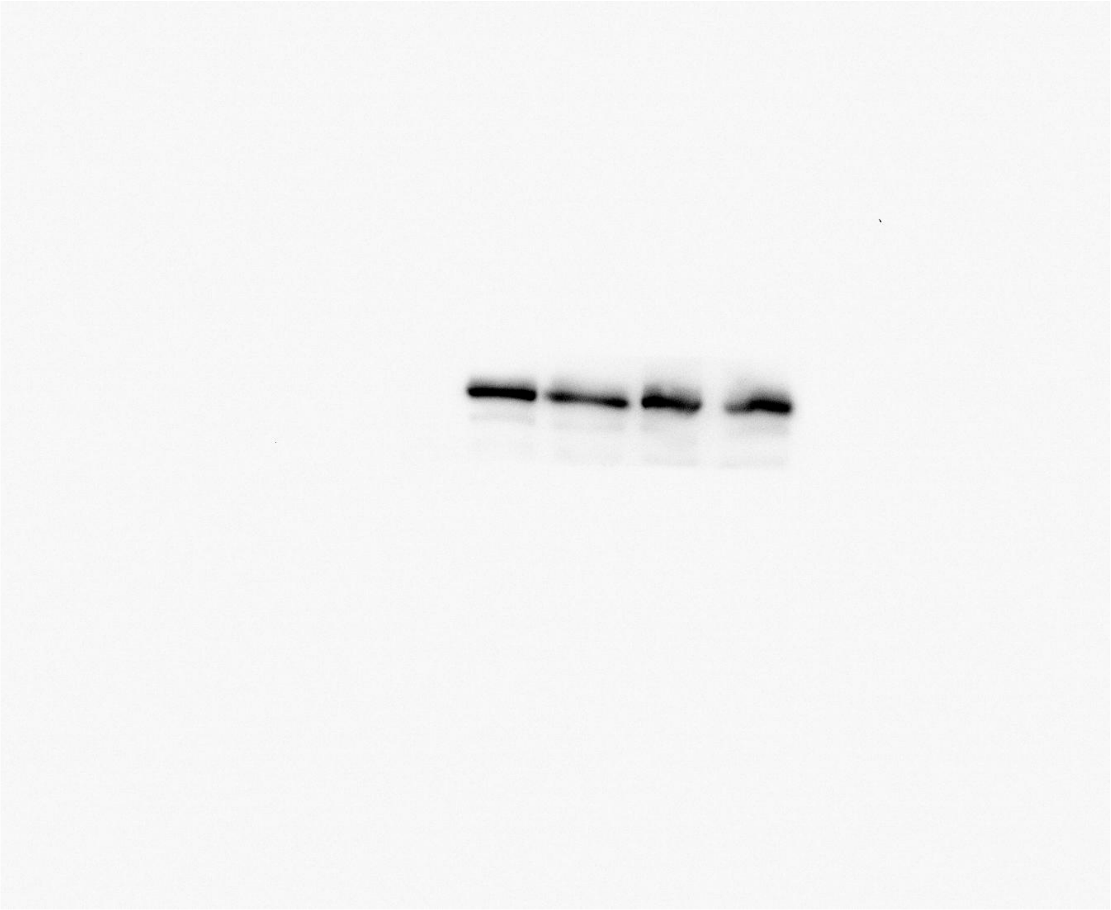

74.fig.7A-2-occludin

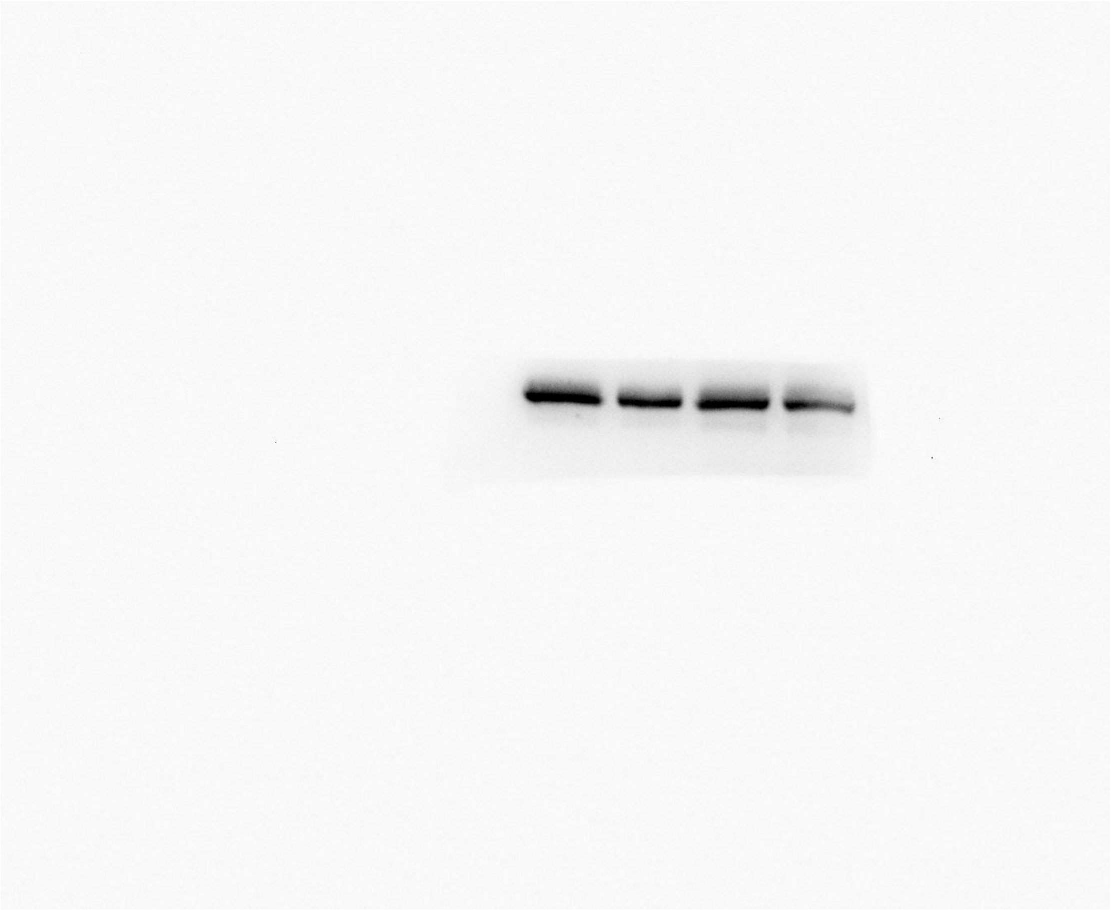

75.fig.7A-3-occludin

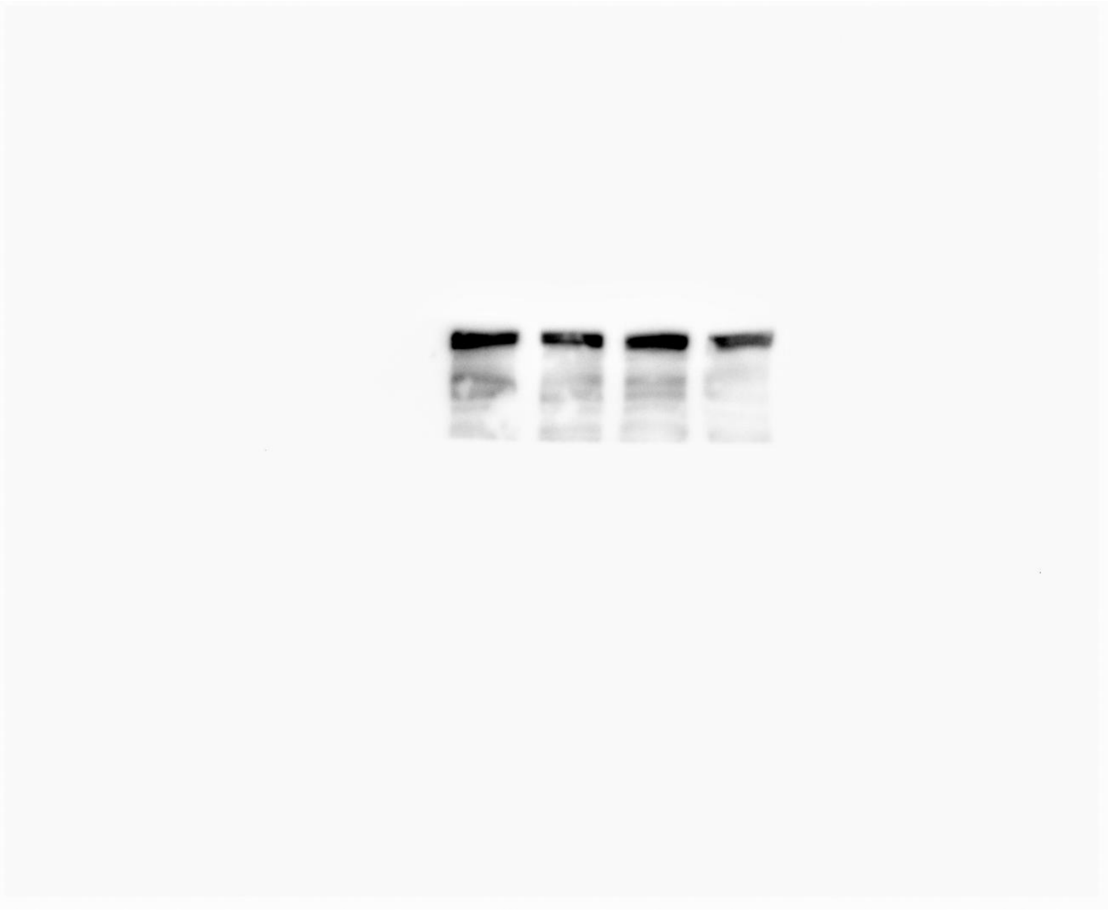

76.fig.7A-1-zo

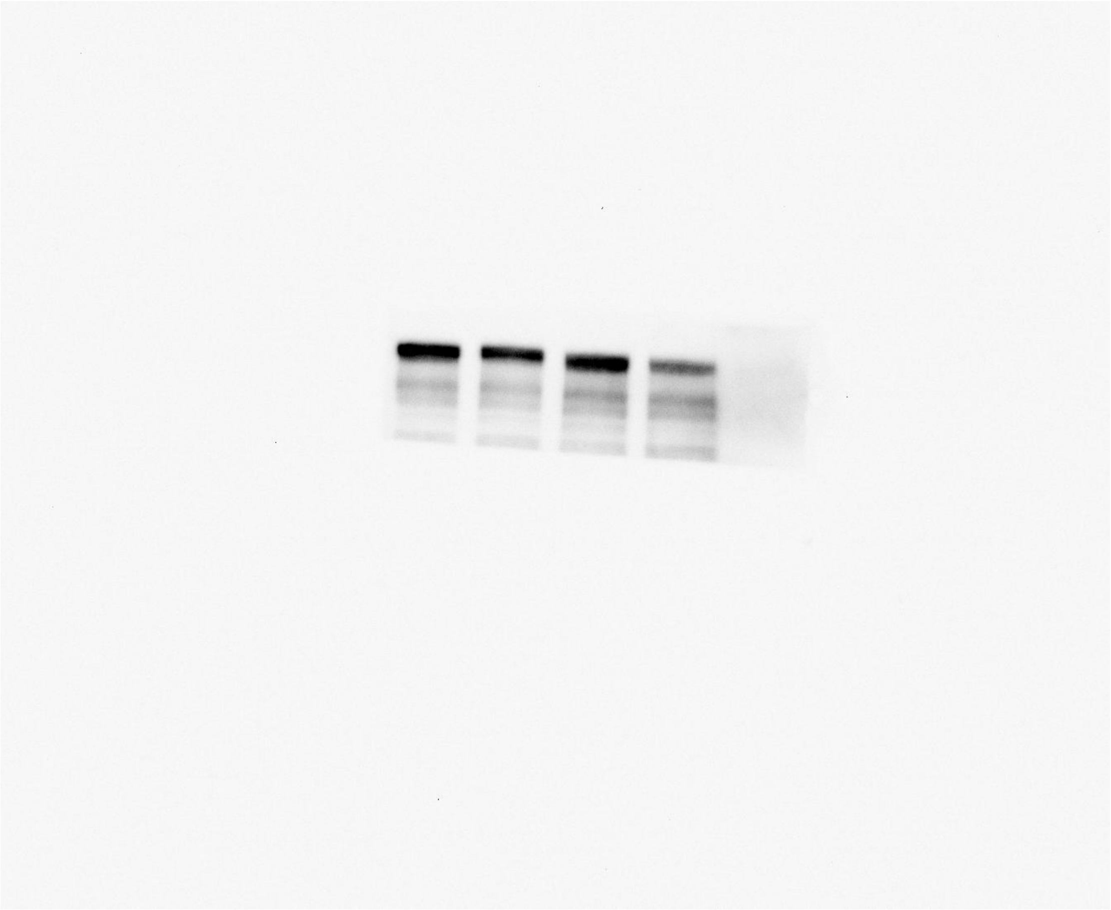

77.fig.7A-2-zo

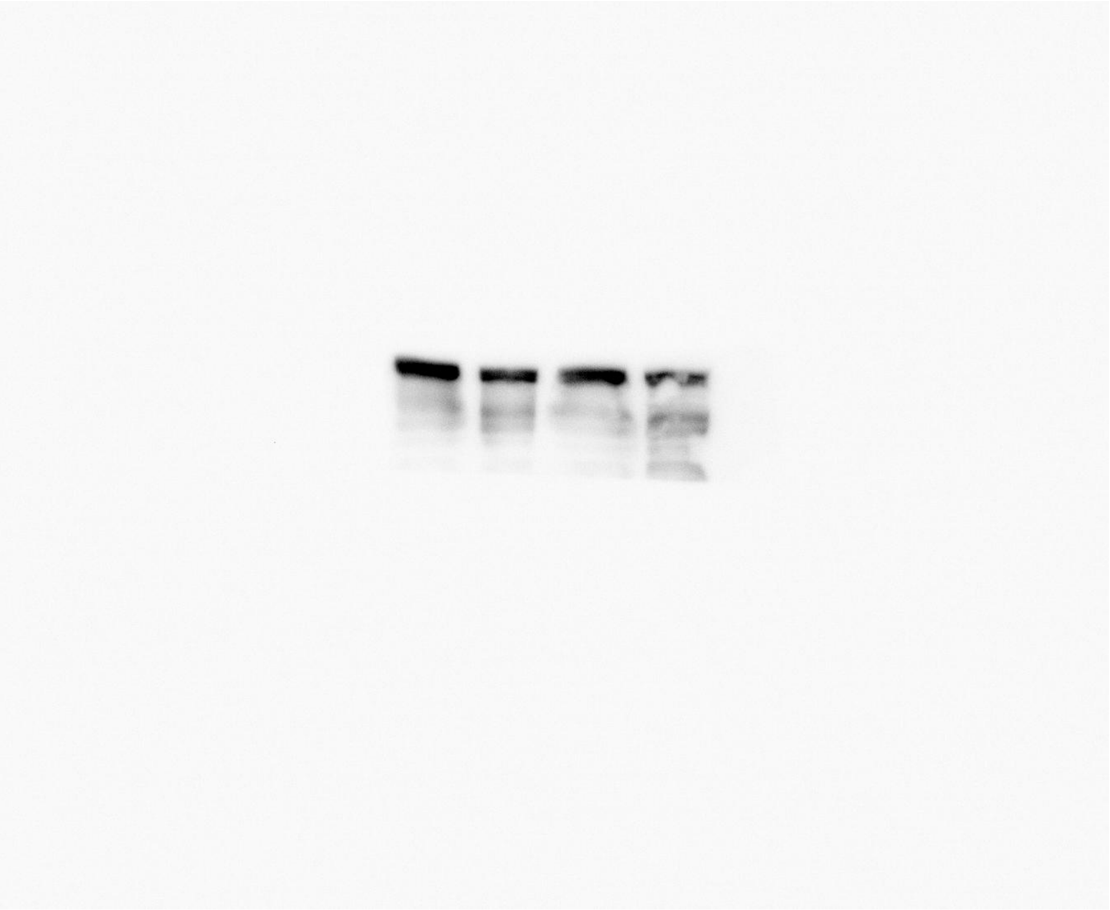

78.fig.7A-3-zo

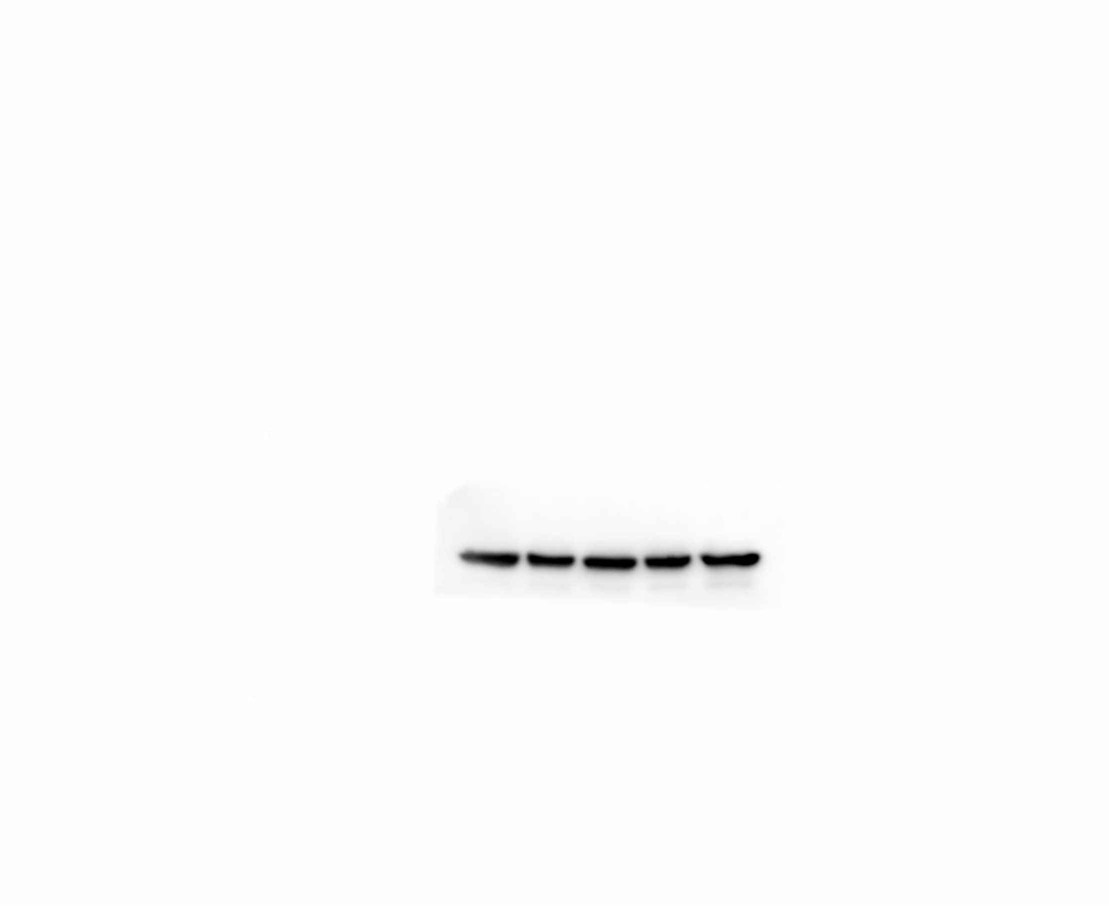

79.fig.S4A-1-gap

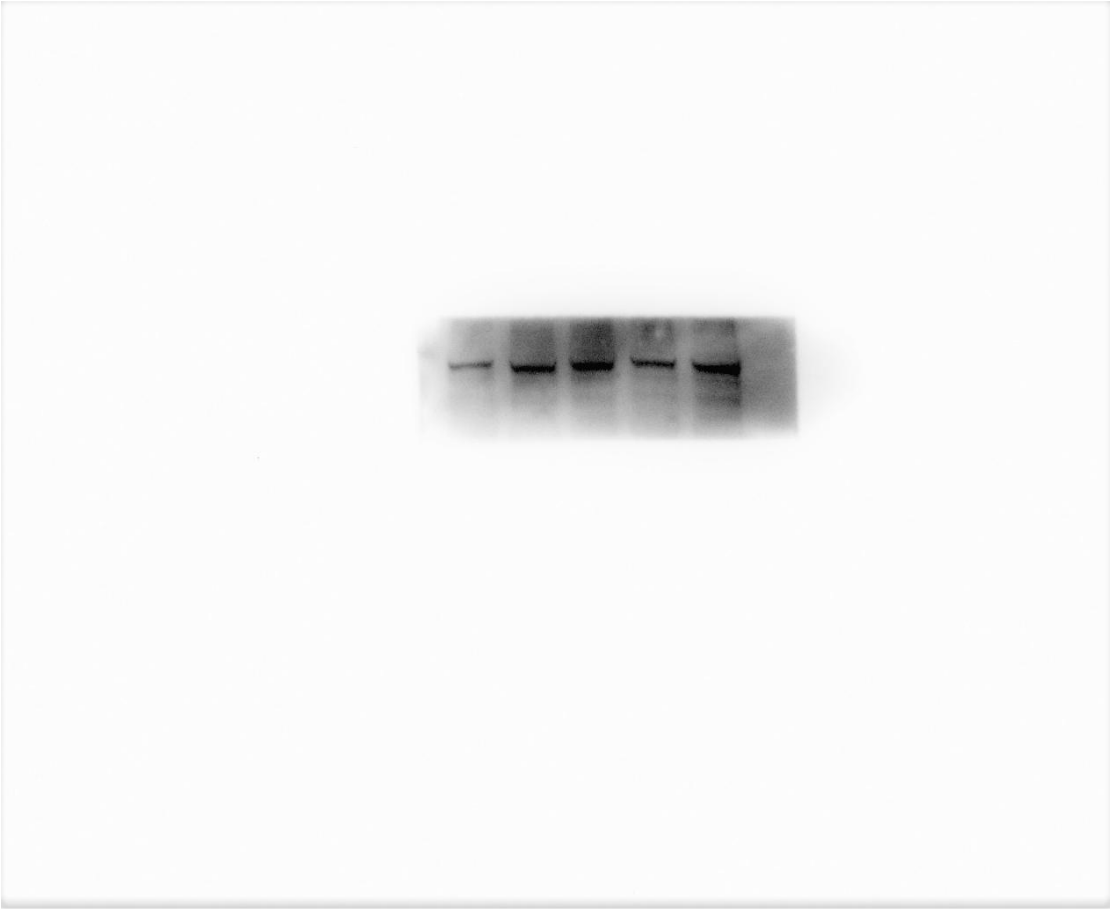

80.fig.S4A-1-pigr

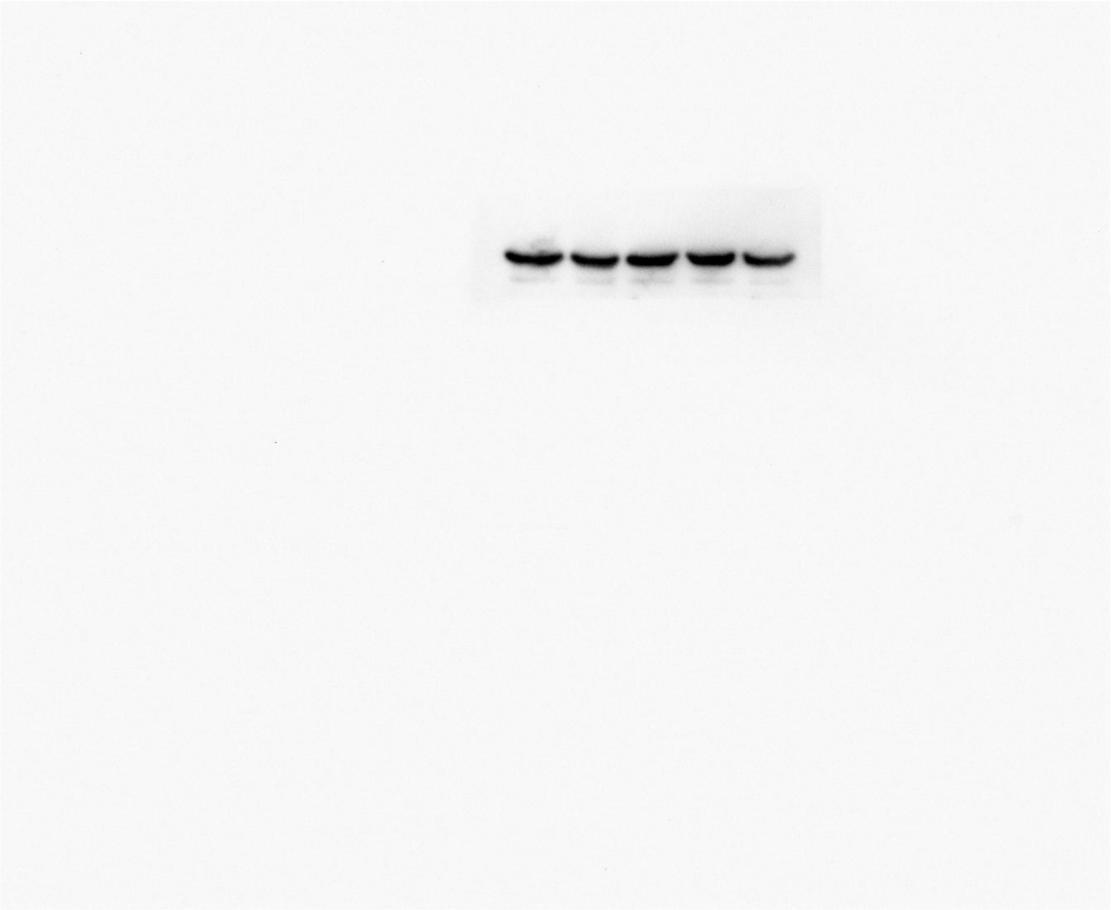

81.fig.S4A-2-gap

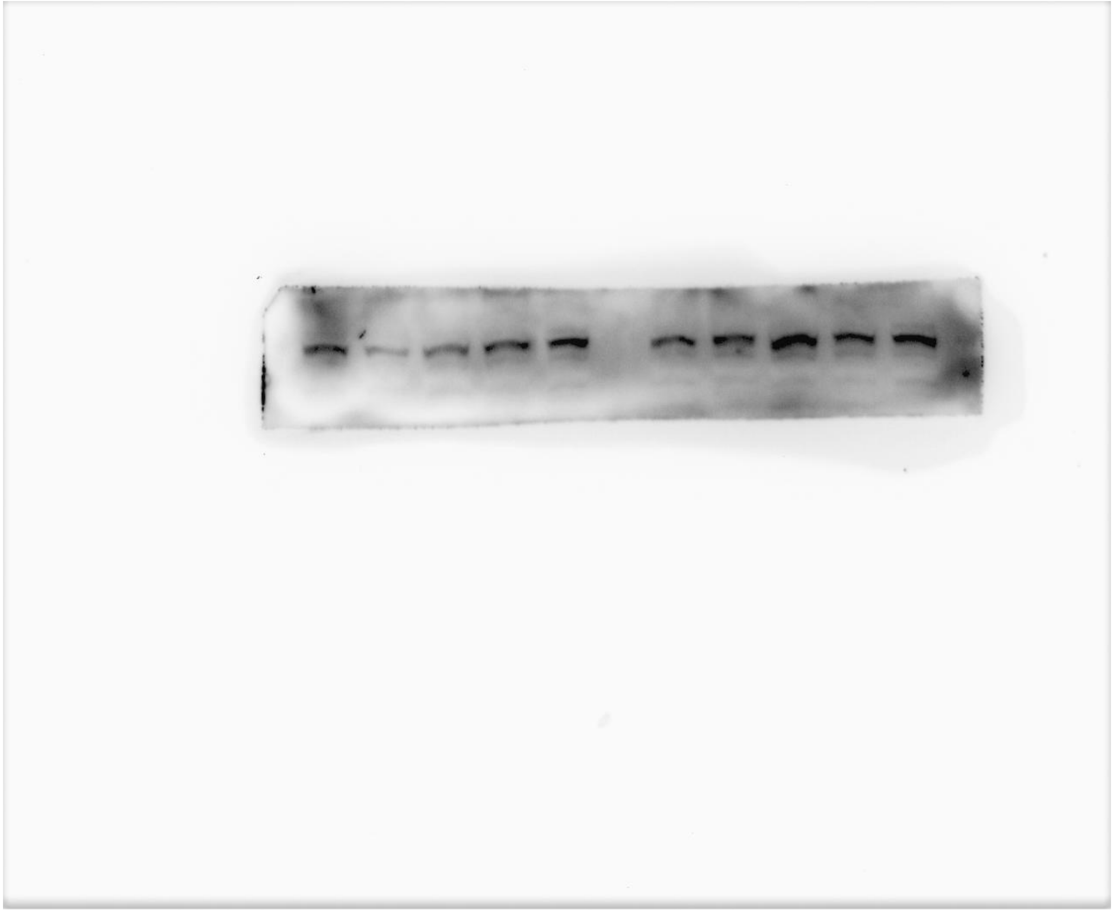

82.fig.S4A-2-pigr-right

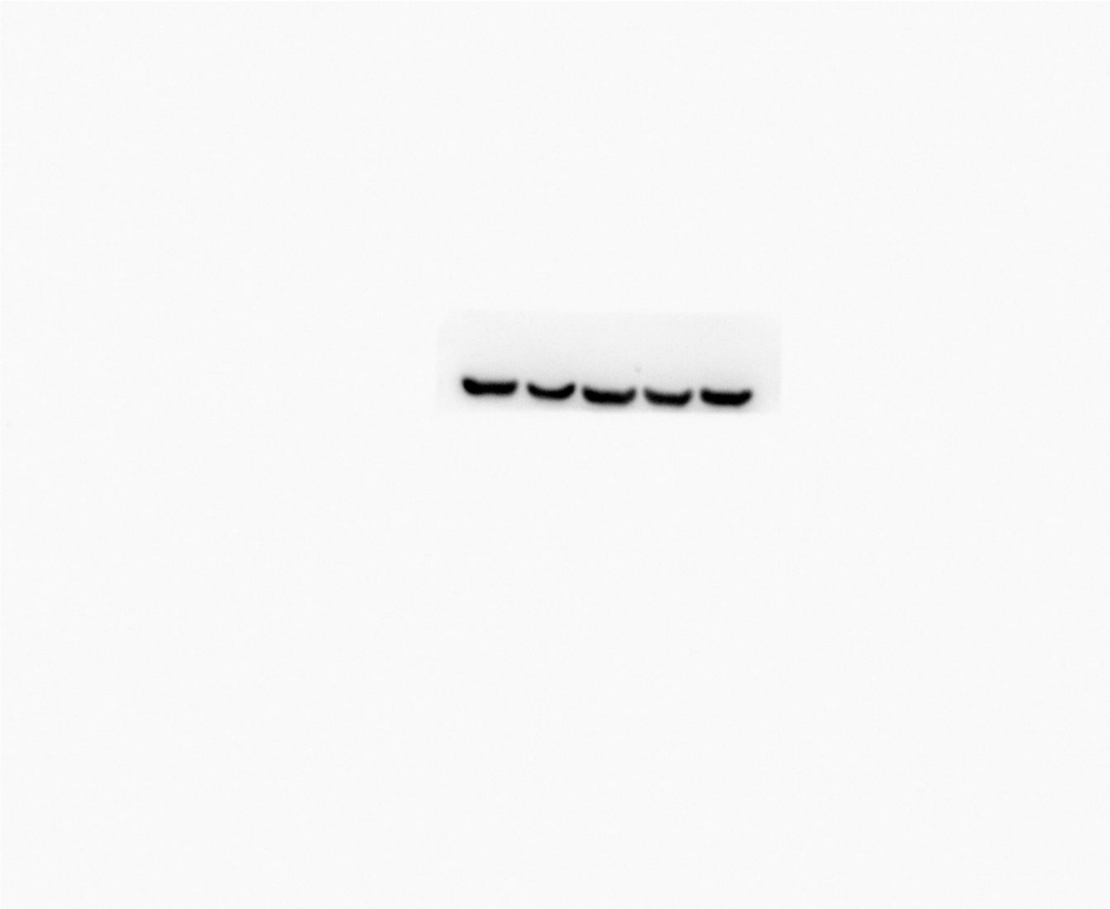

83.fig.S4A-3-gap

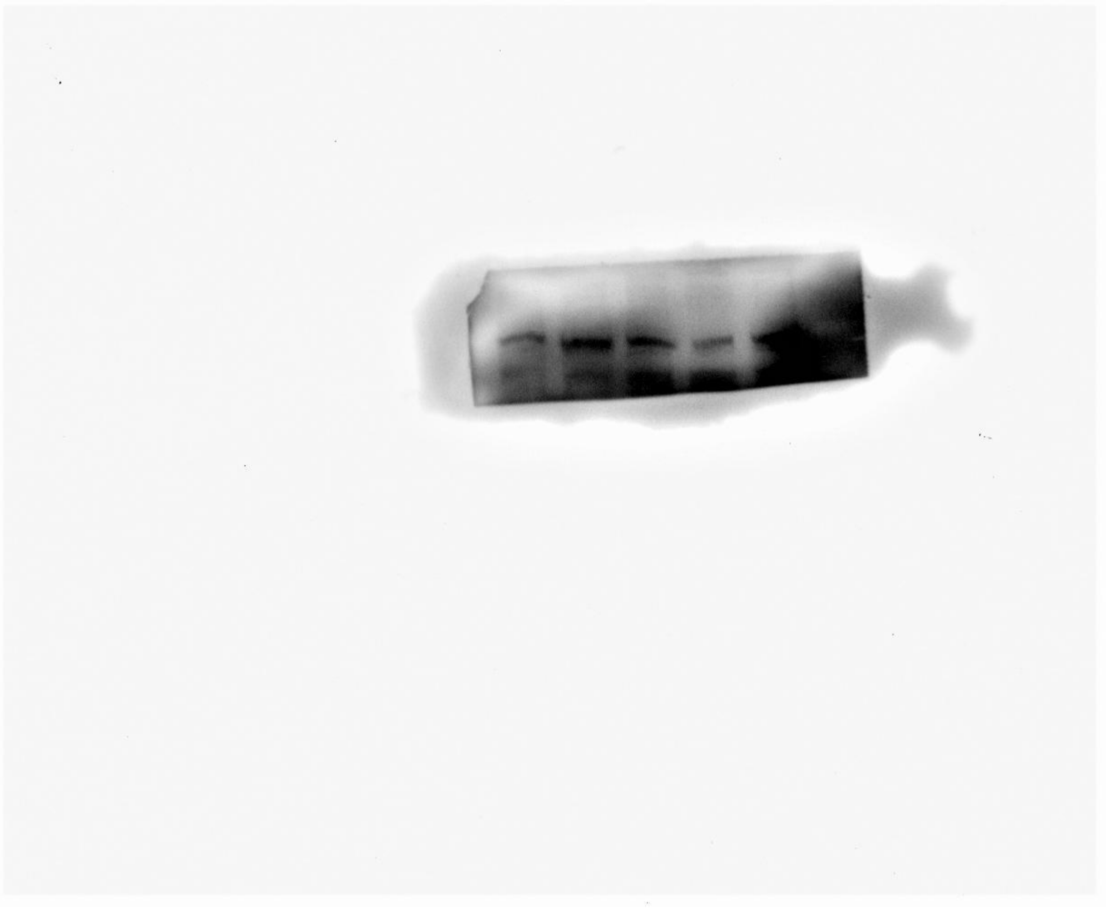

84.fig.S4A-3-pigr

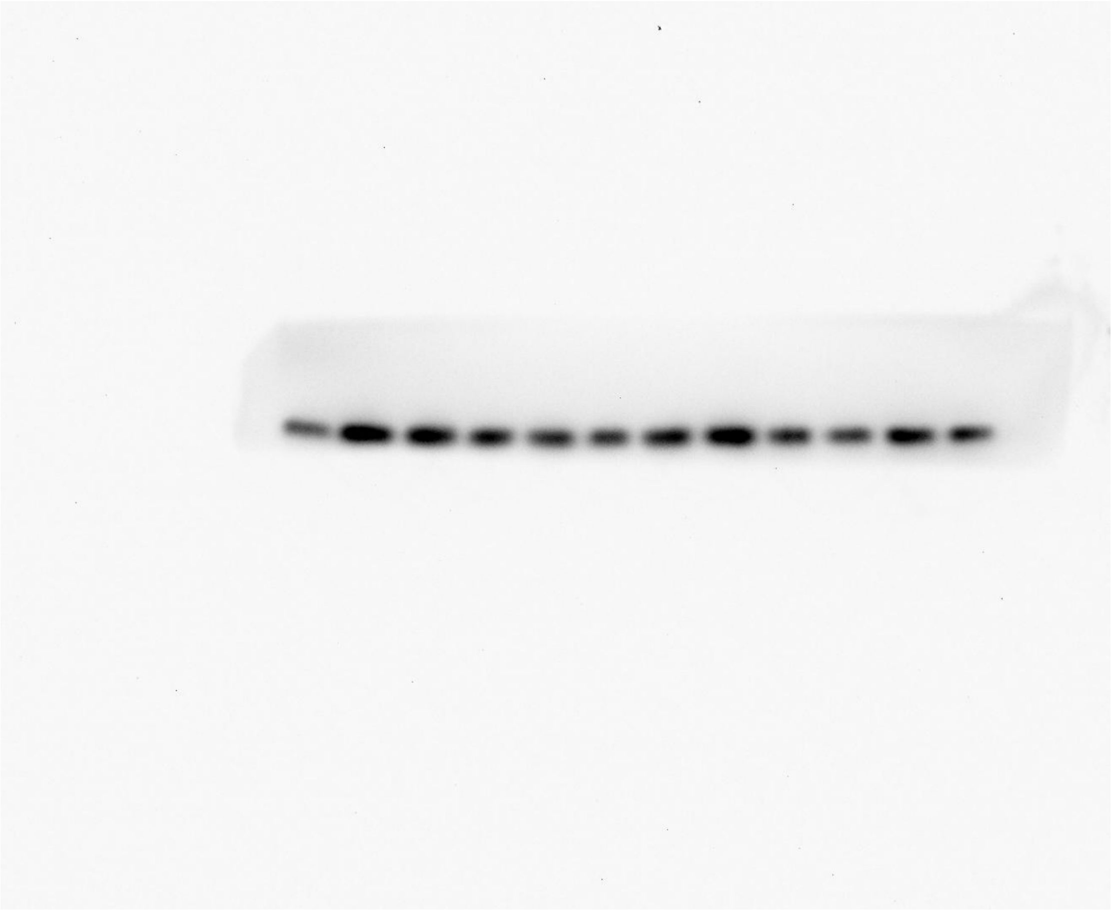

85.fig.S4B-1-claudin-right

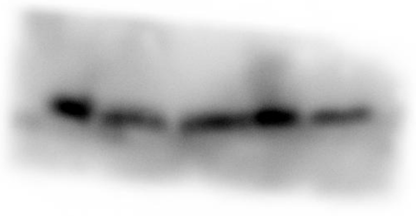

86.fig.S4B-2-claudin

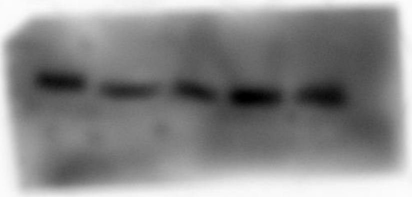

87.fig.S4B-3-claudin

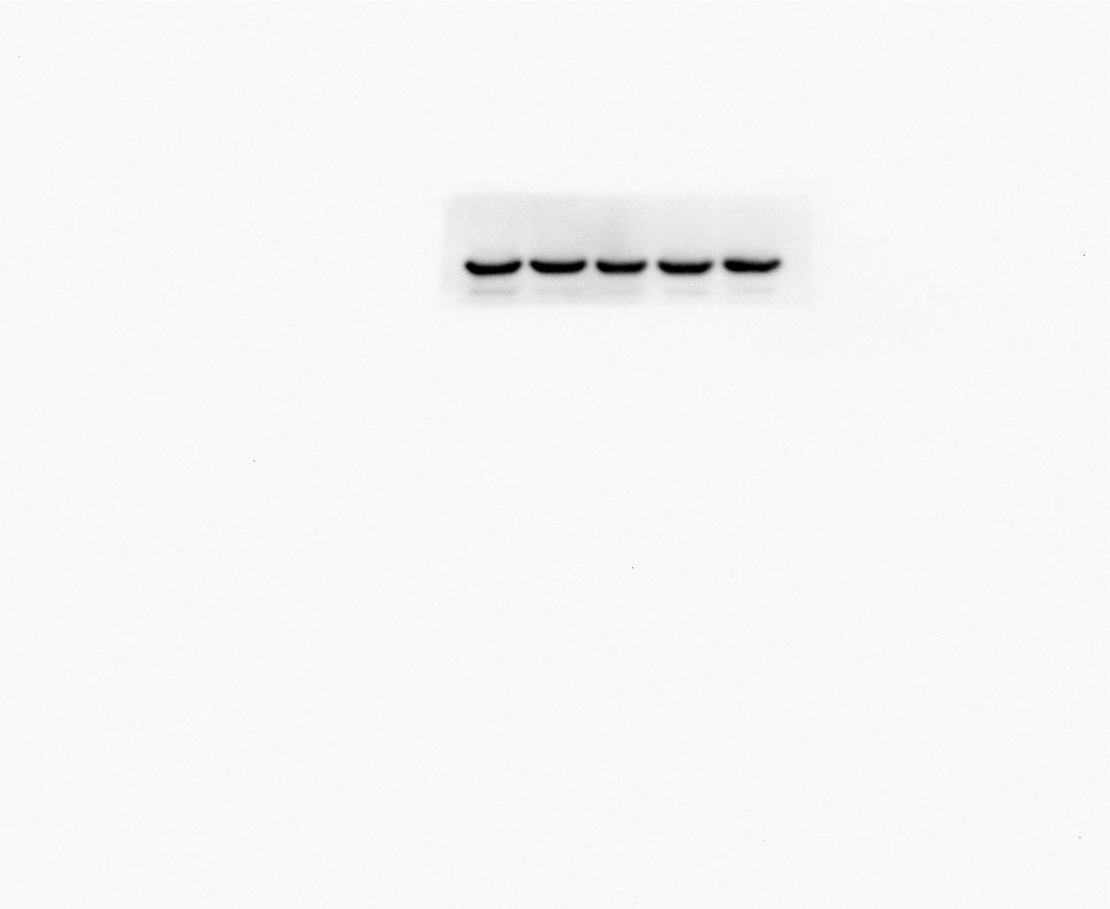

88.fig.S4B-1-gap

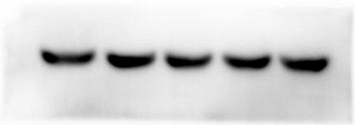

89.fig.S4B-2-gap

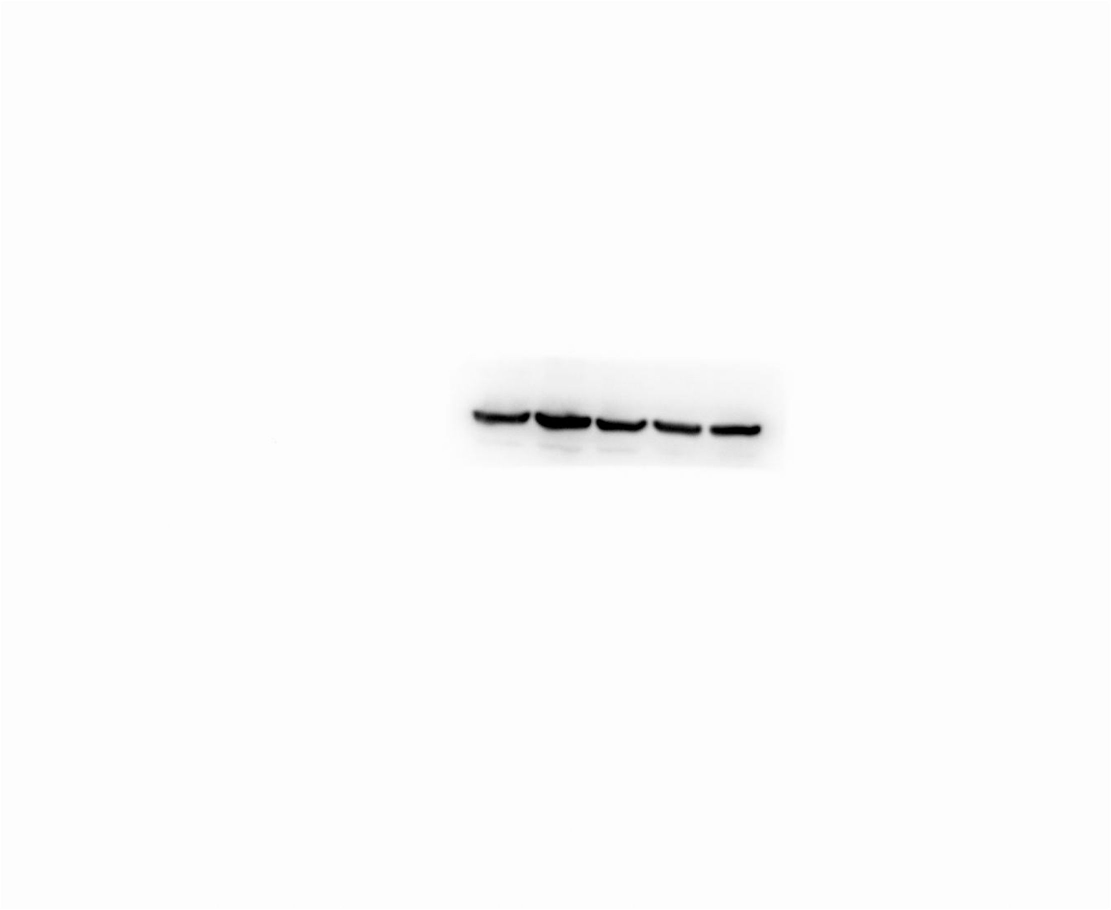

90.fig.S4B-3-gap

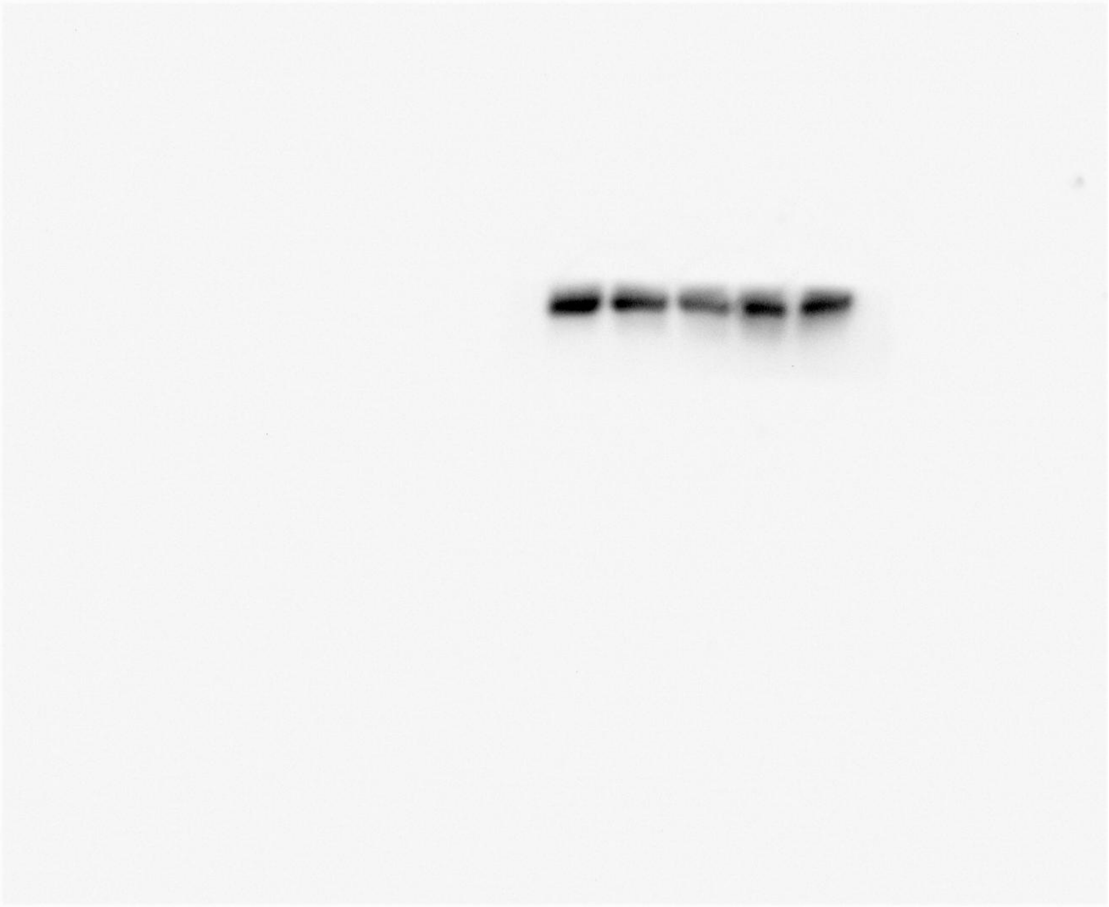

91.fig.S4B-1-occludin

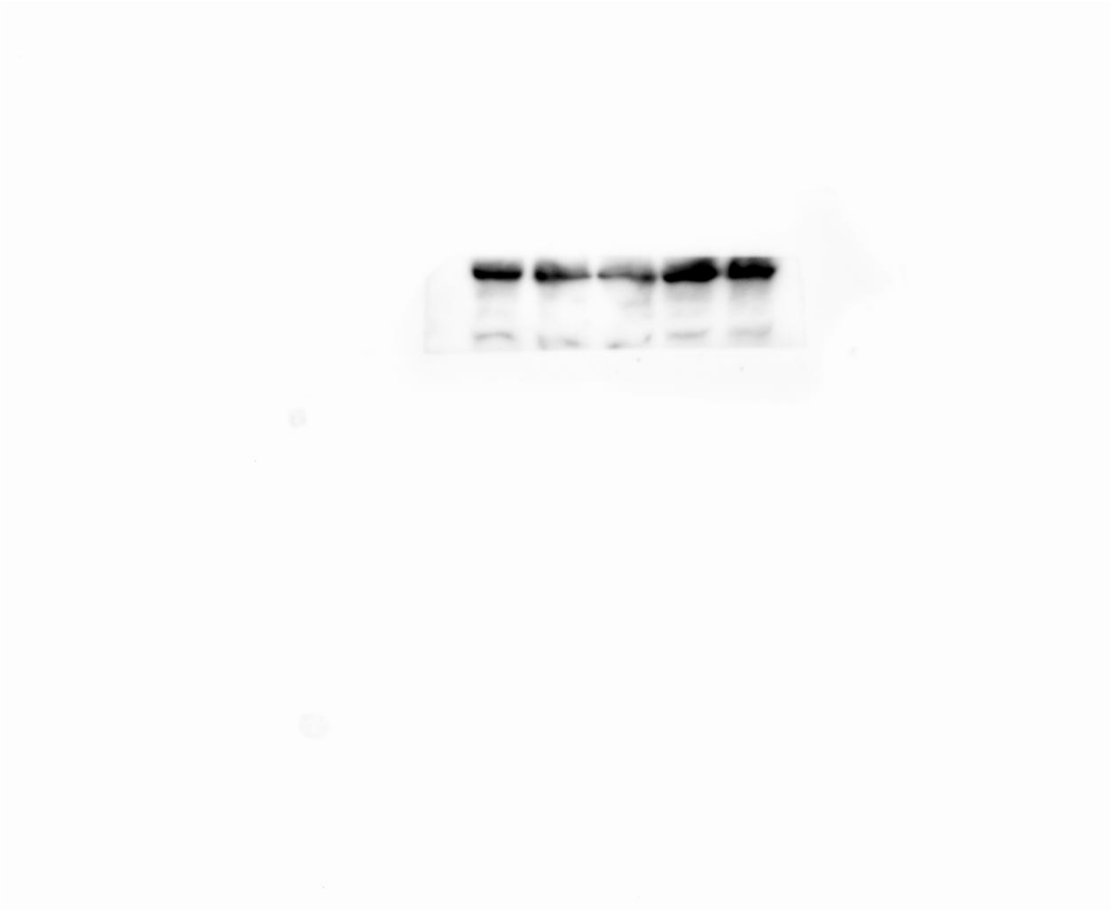

92.fig.S4B-2-occludin

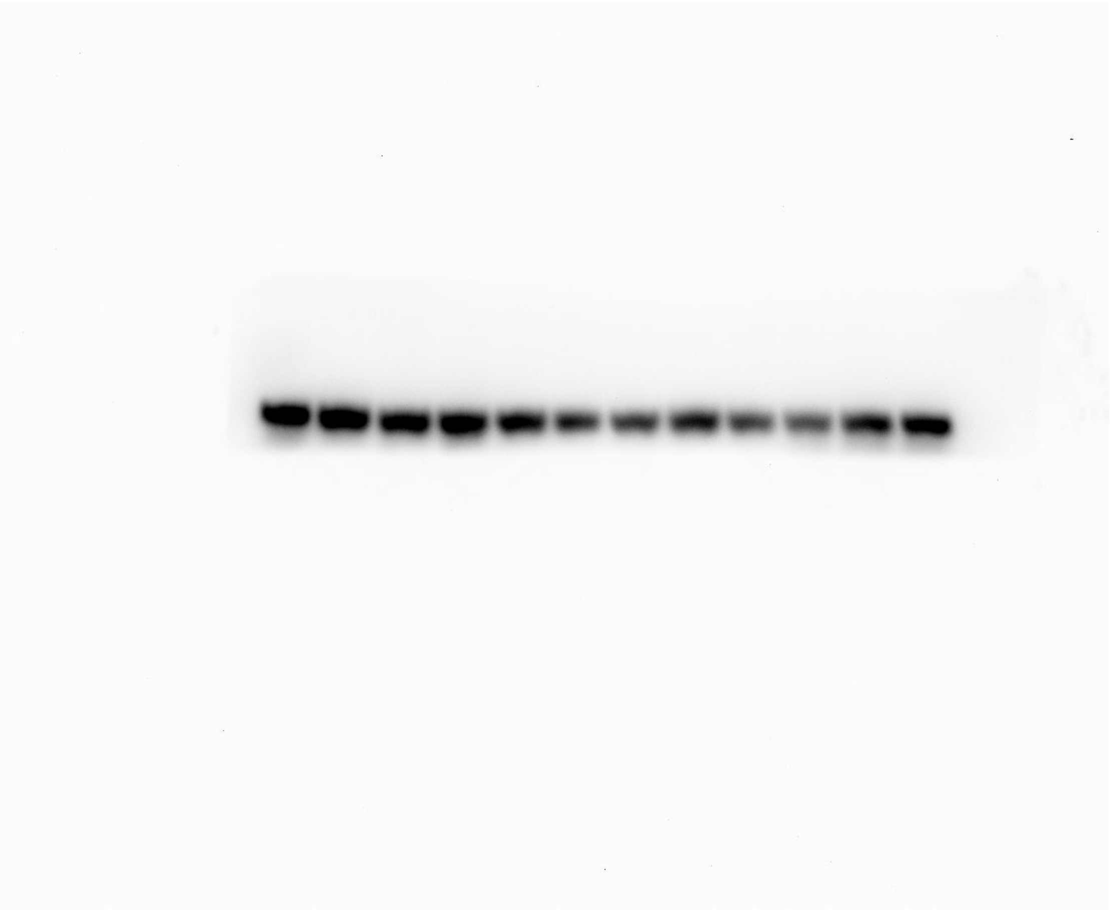

93.fig.S4B-3-occludin-right

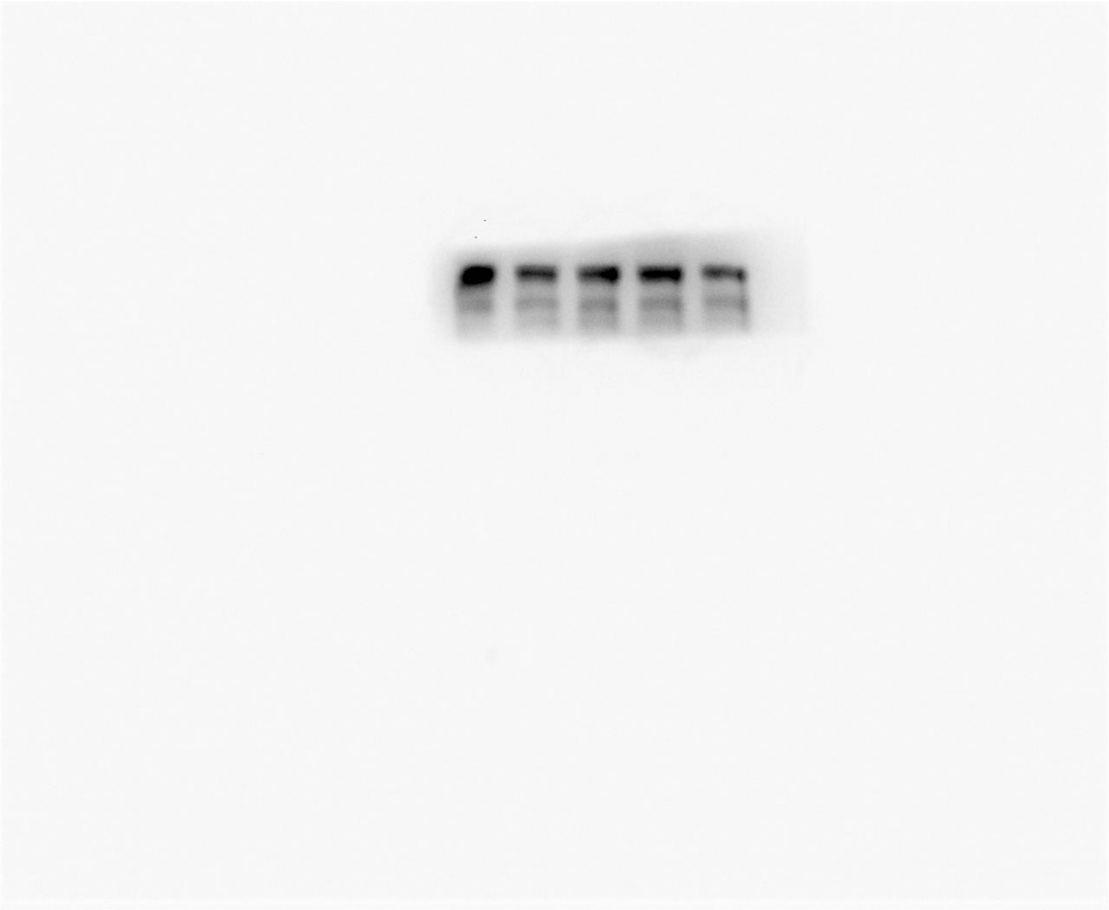

94.fig.S4B-1-zo

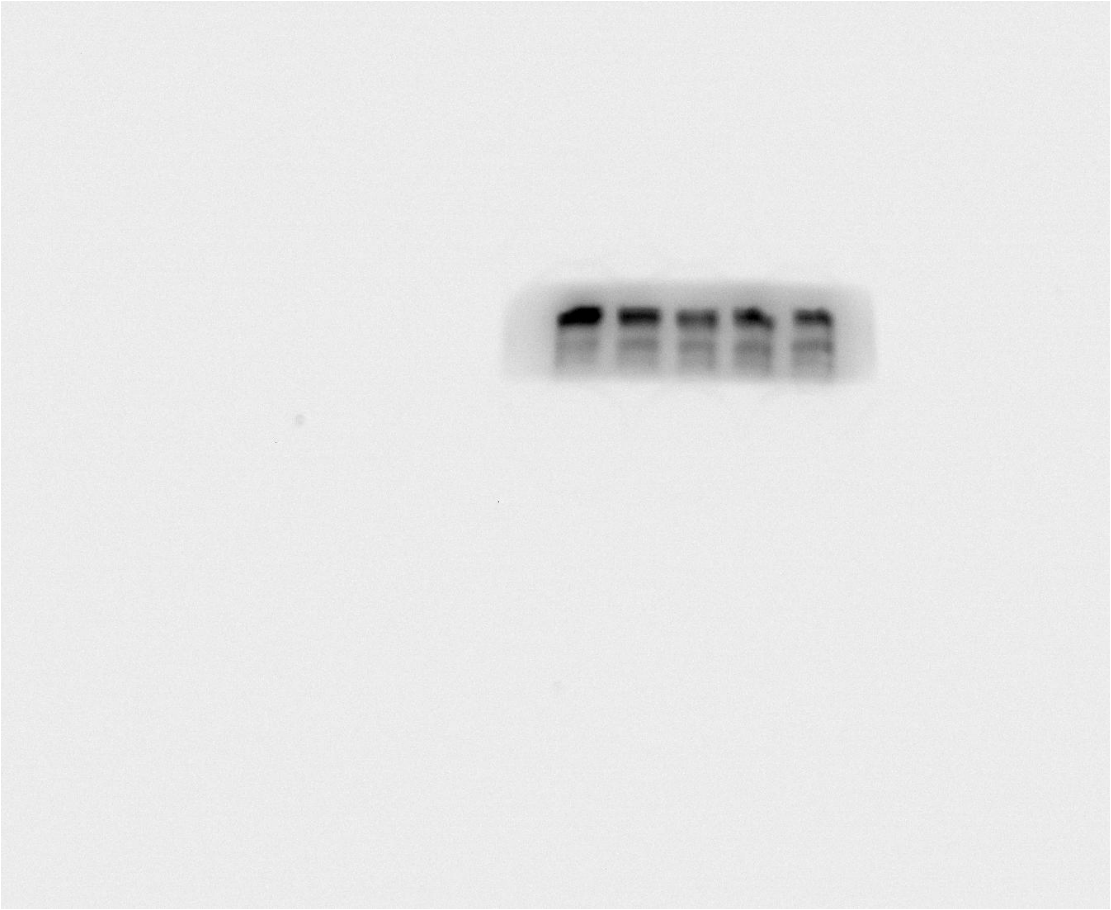

95.fig.S4B-2-zo

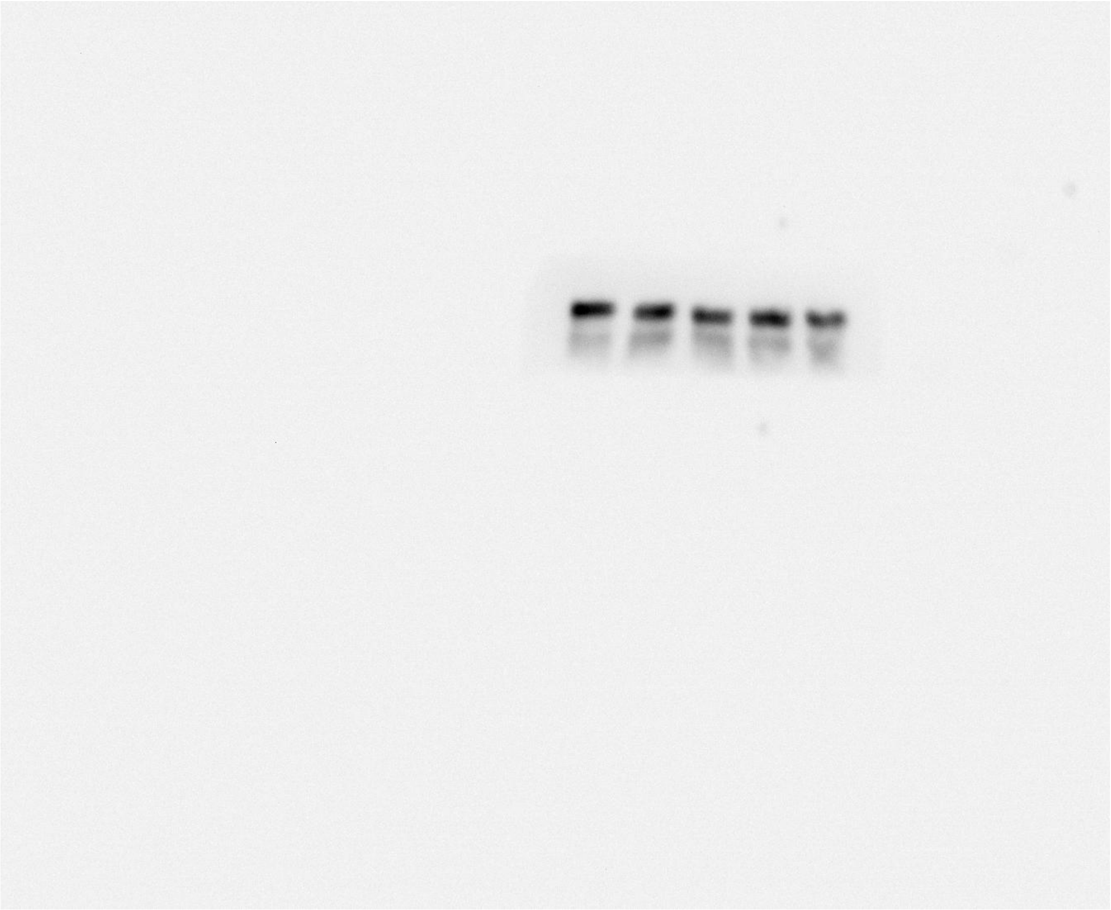

96.fig.S4B-3-zo

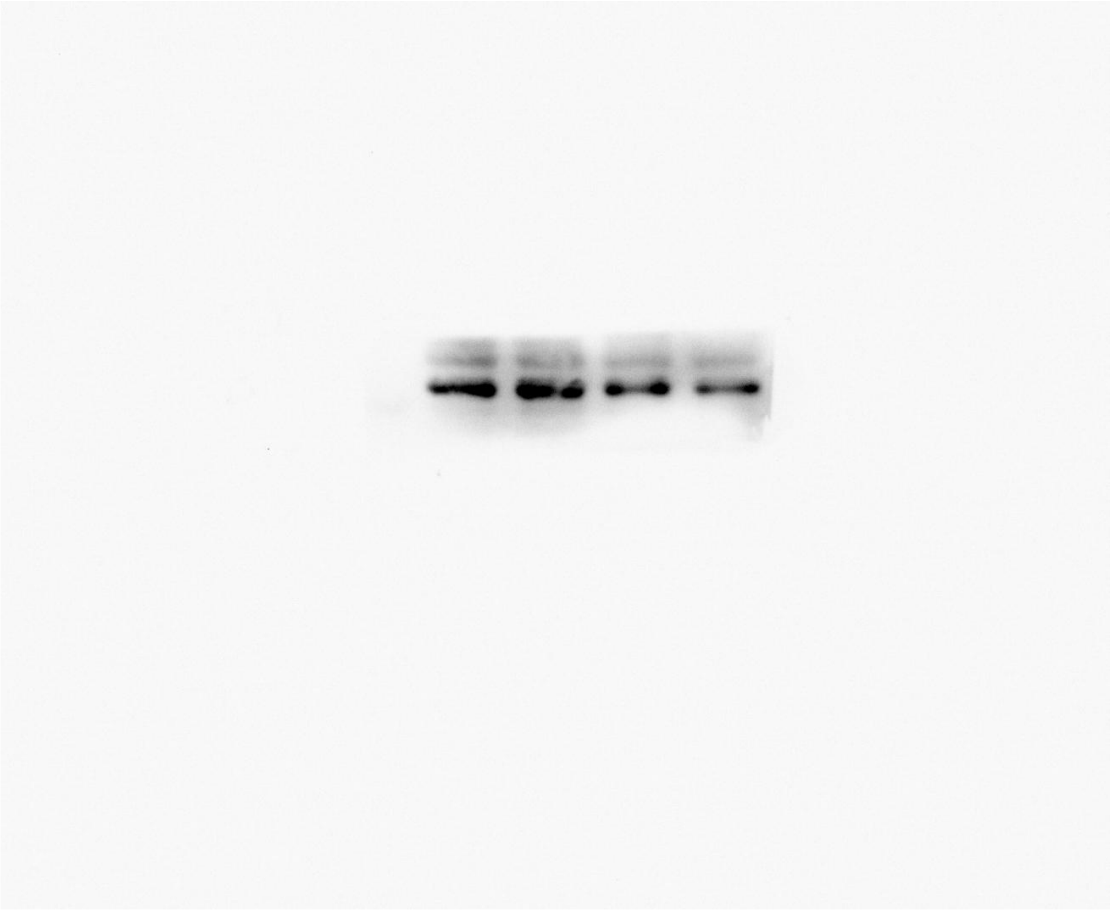

97.fig.S4C-1-claudin

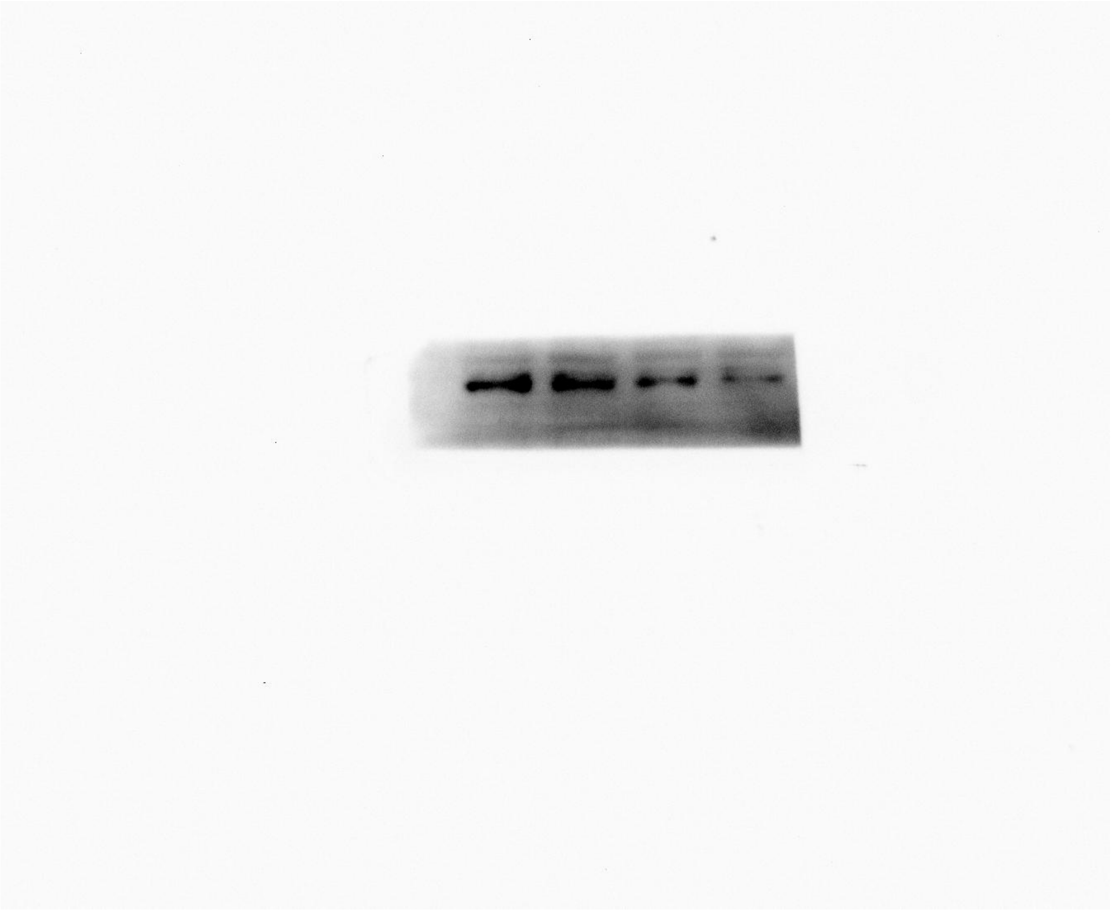

98.fig.S4C-2-claudin

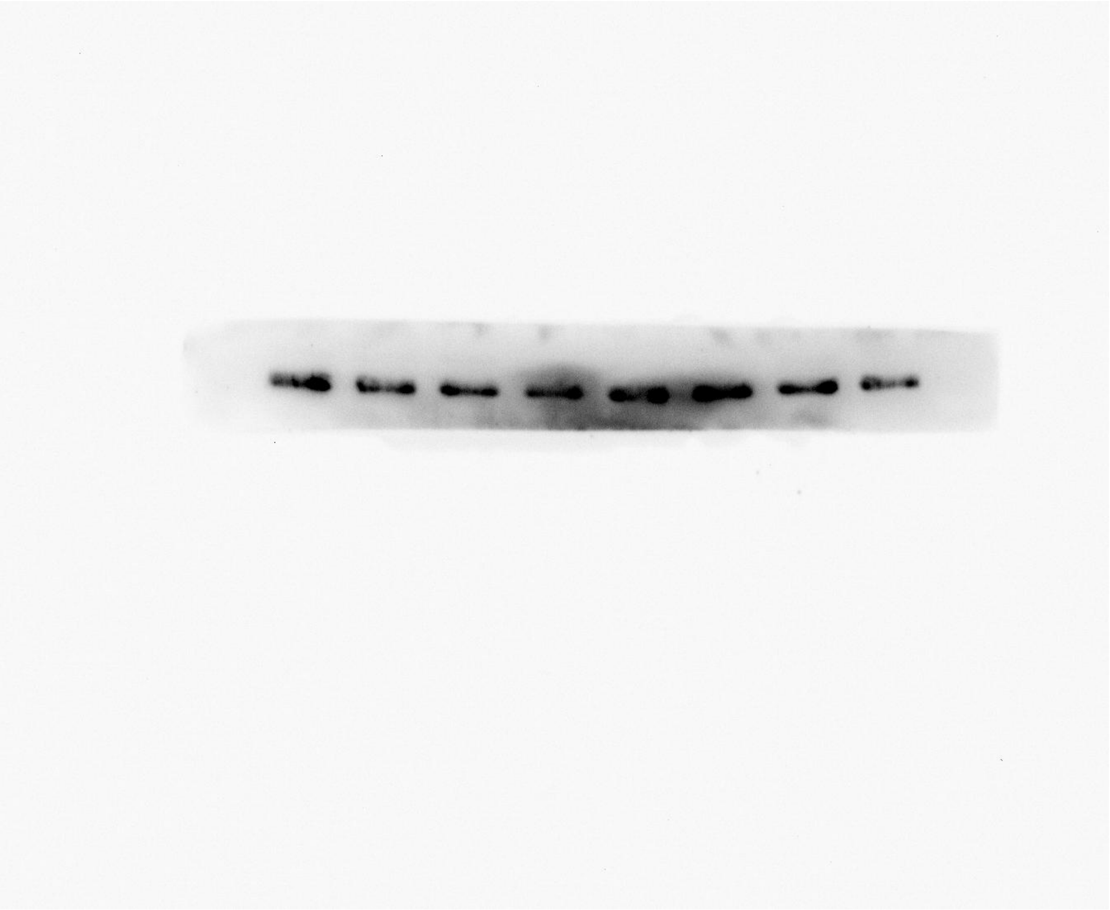

99.fig.S4C-3-claudin-right

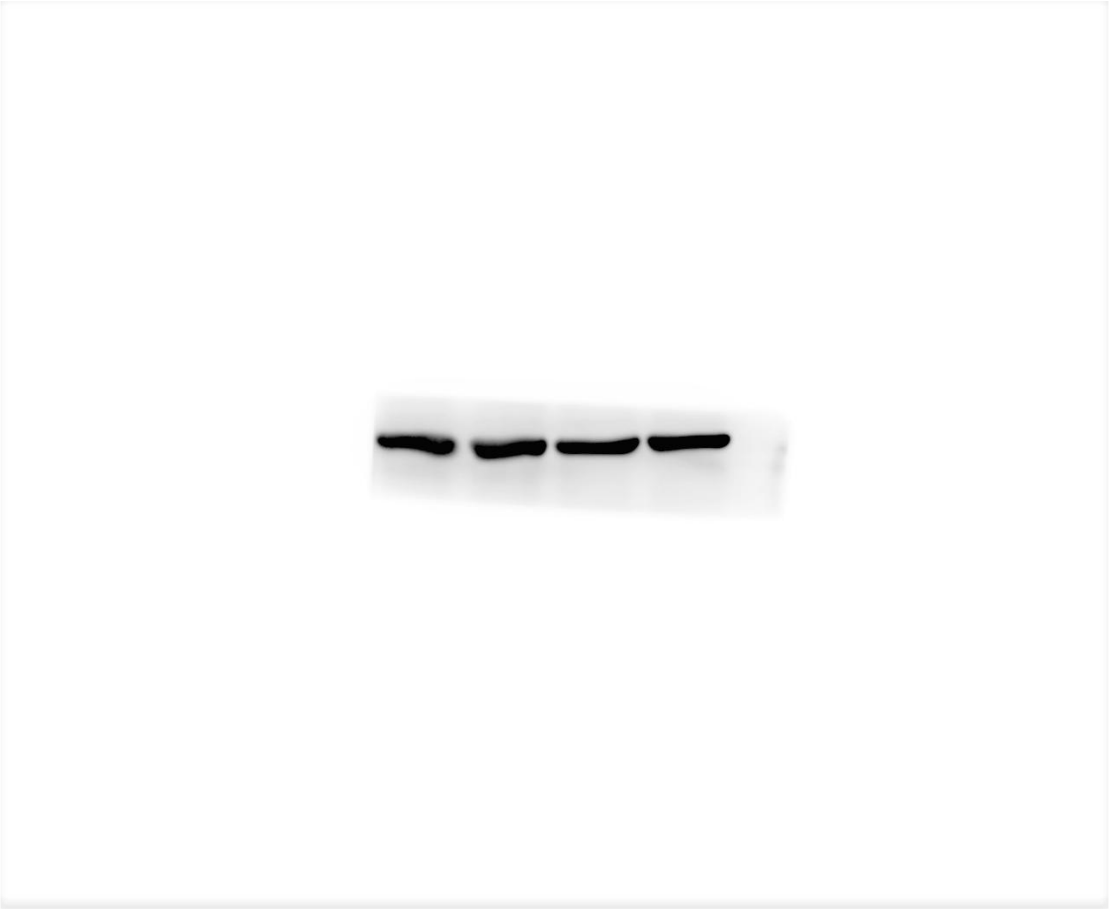

100.fig.S4C-1-gap

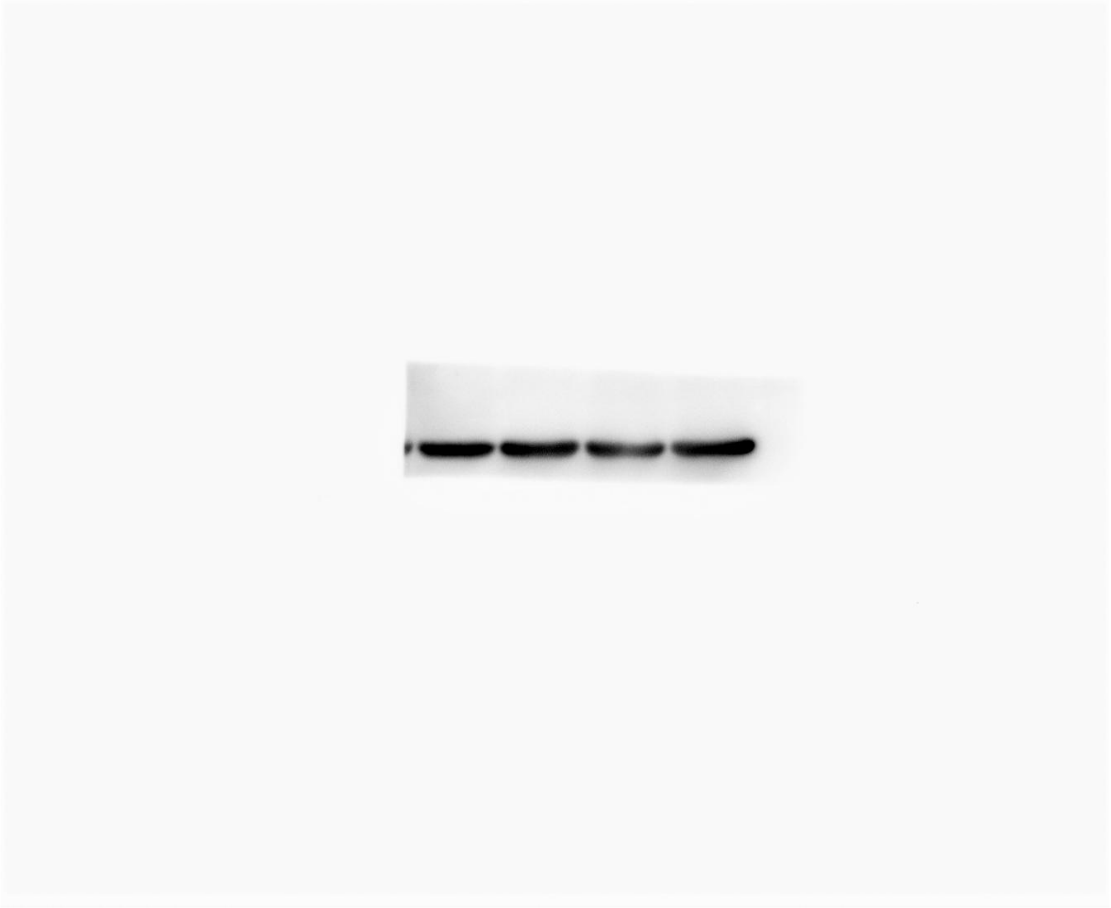

101.fig.S4C-2-gap

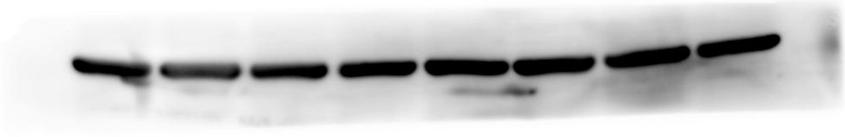

102.fig.S4C-3-gap-left

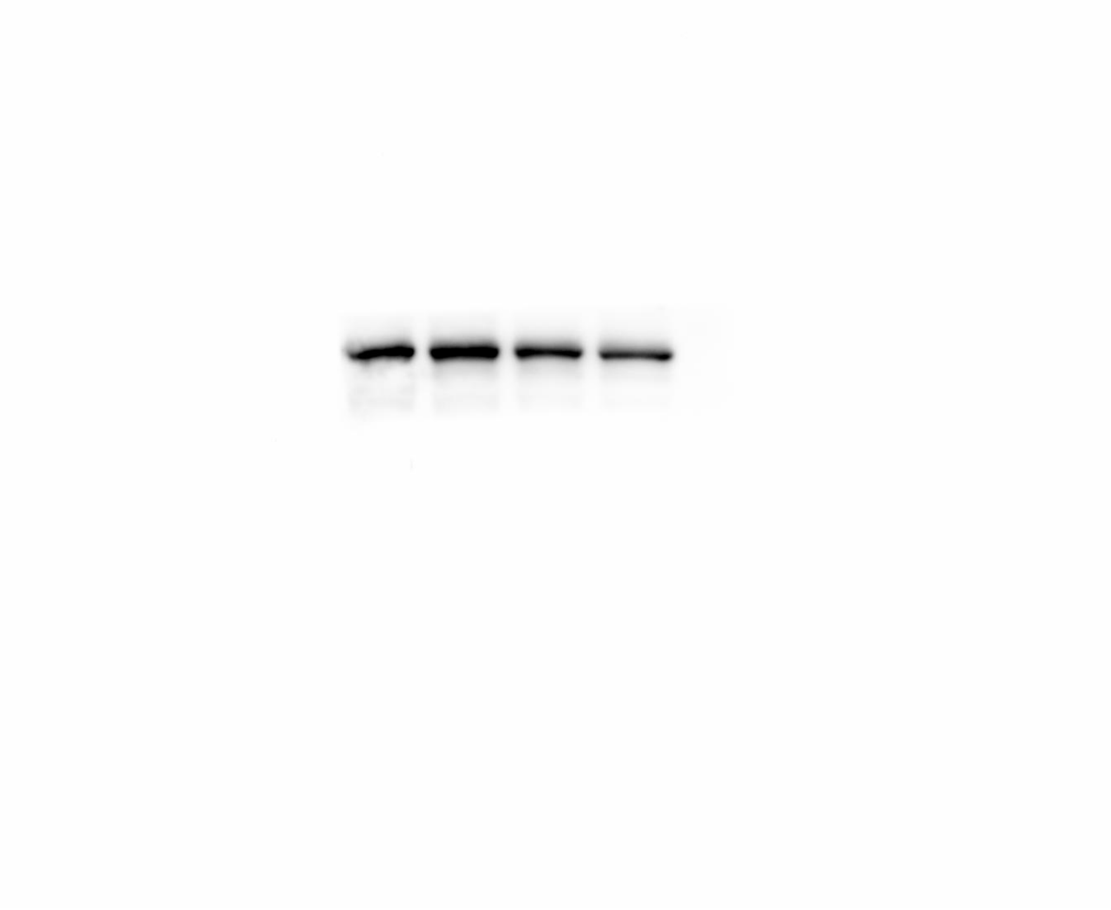

103.fig.S4C-1-occludin

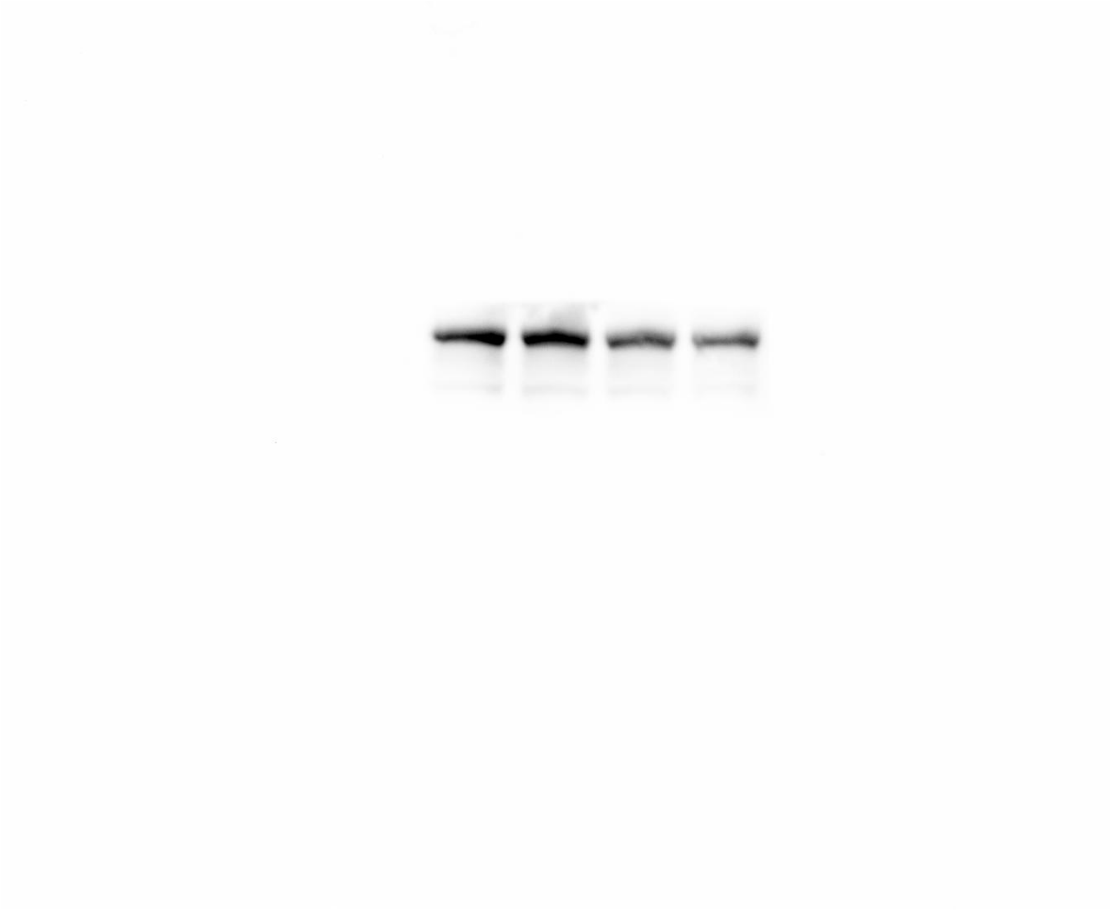

104.fig.S4C-2-occludin

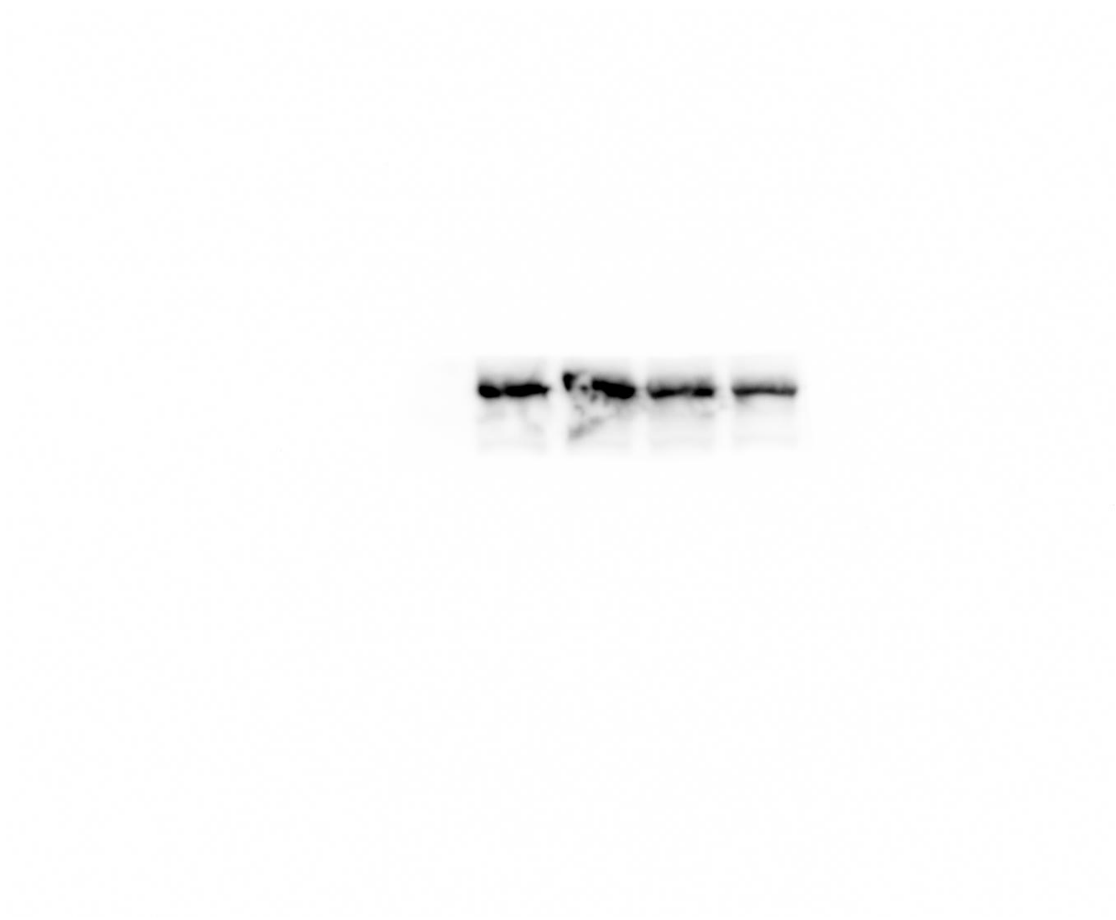

105.fig.S4C-3-occludin

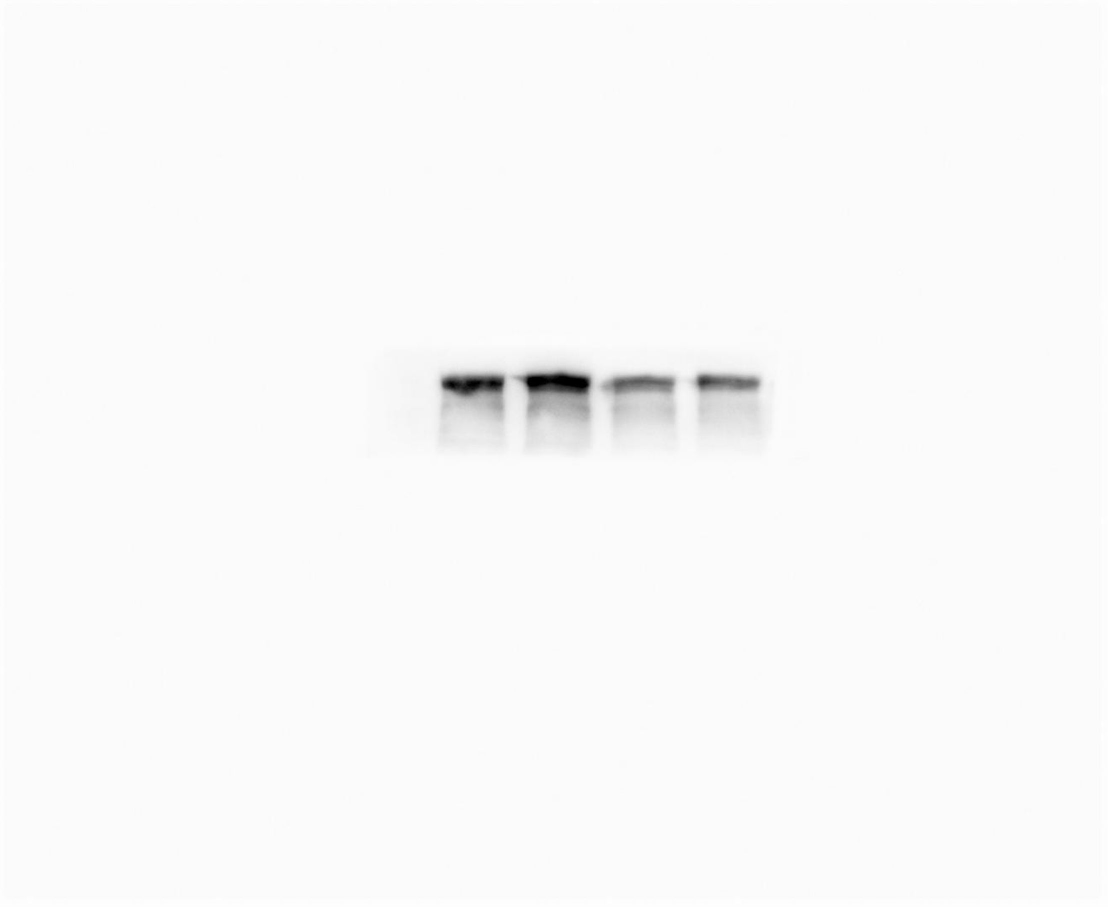

106.fig.S4C-1-zo

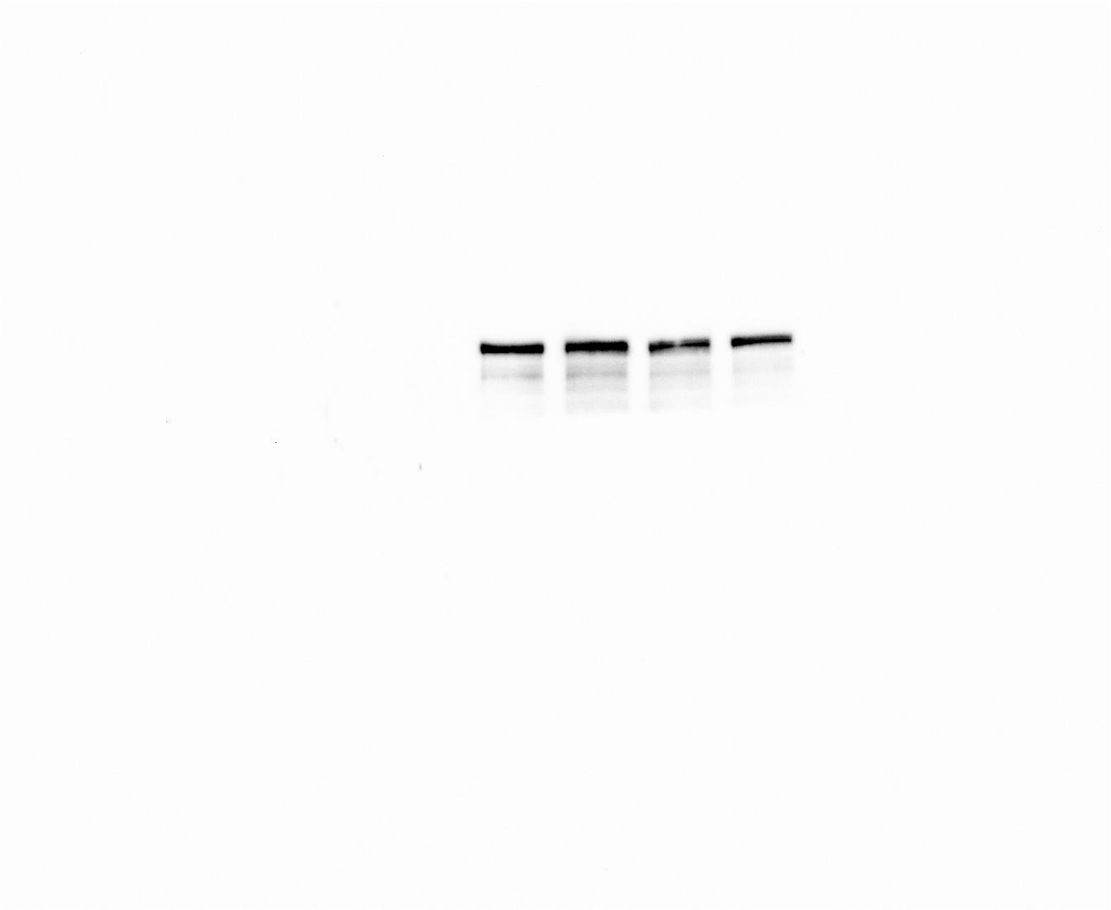

107.fig.S4C-2-zo

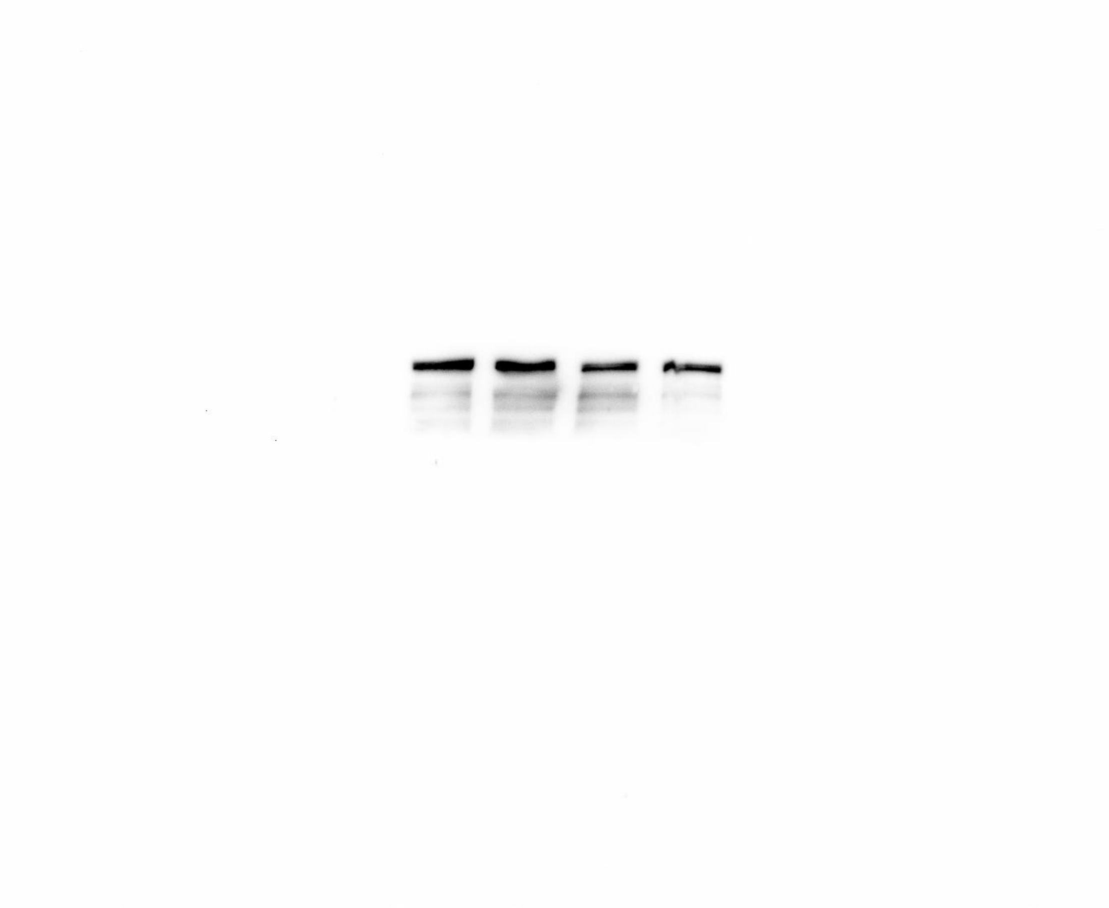

108.fig.S4C-3-zo

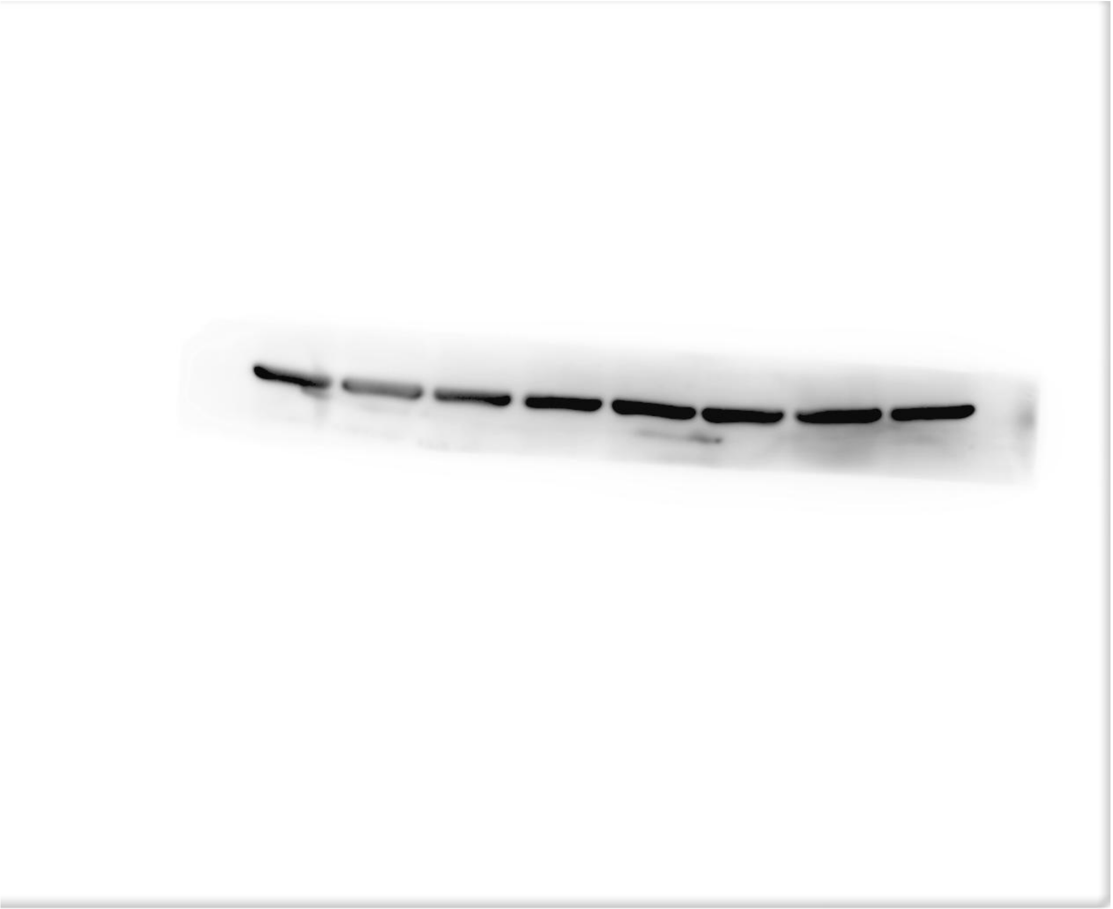

109.fig.S4D-1-gap-right

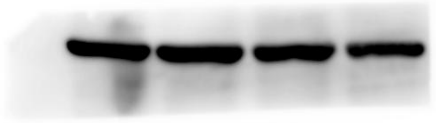

110.fig.S4D-2-gap

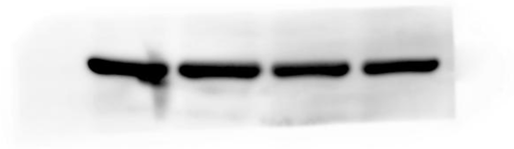

111.fig.S4D-3-gap

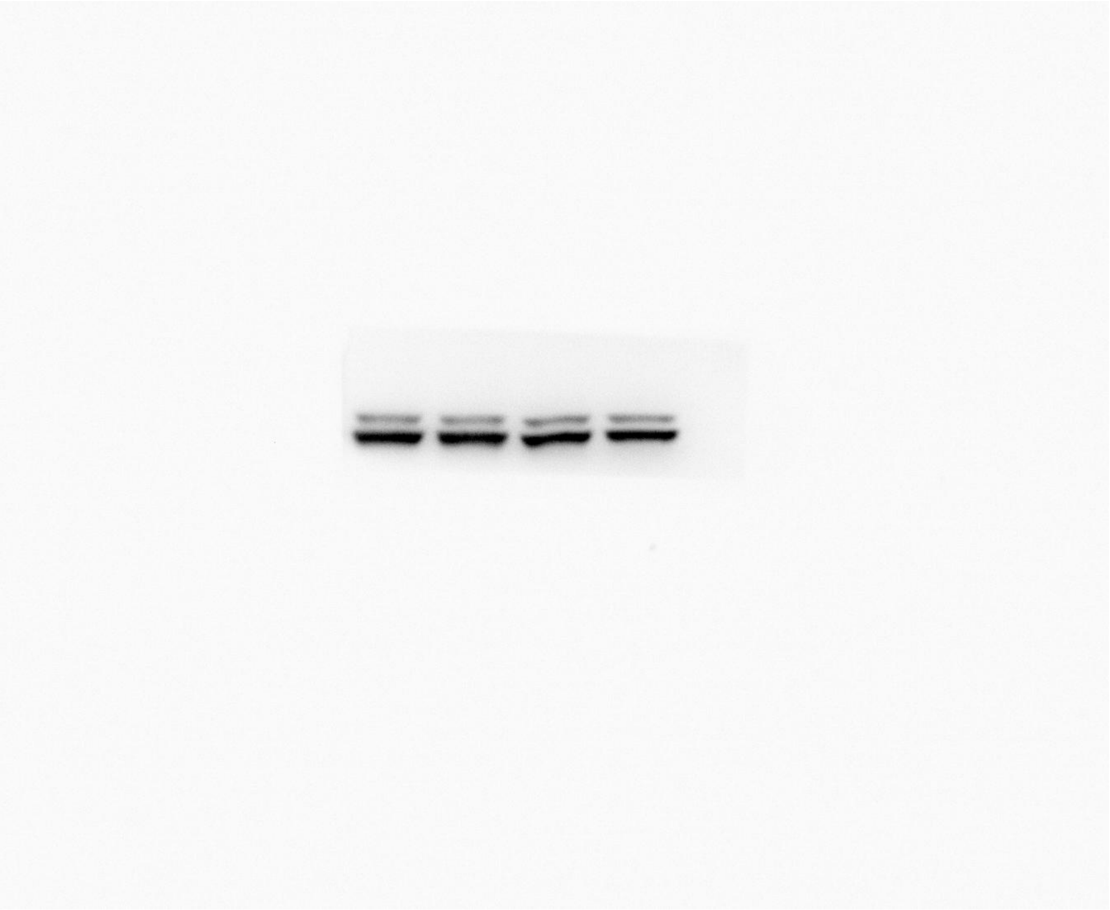

112.fig.S4D-1-erk

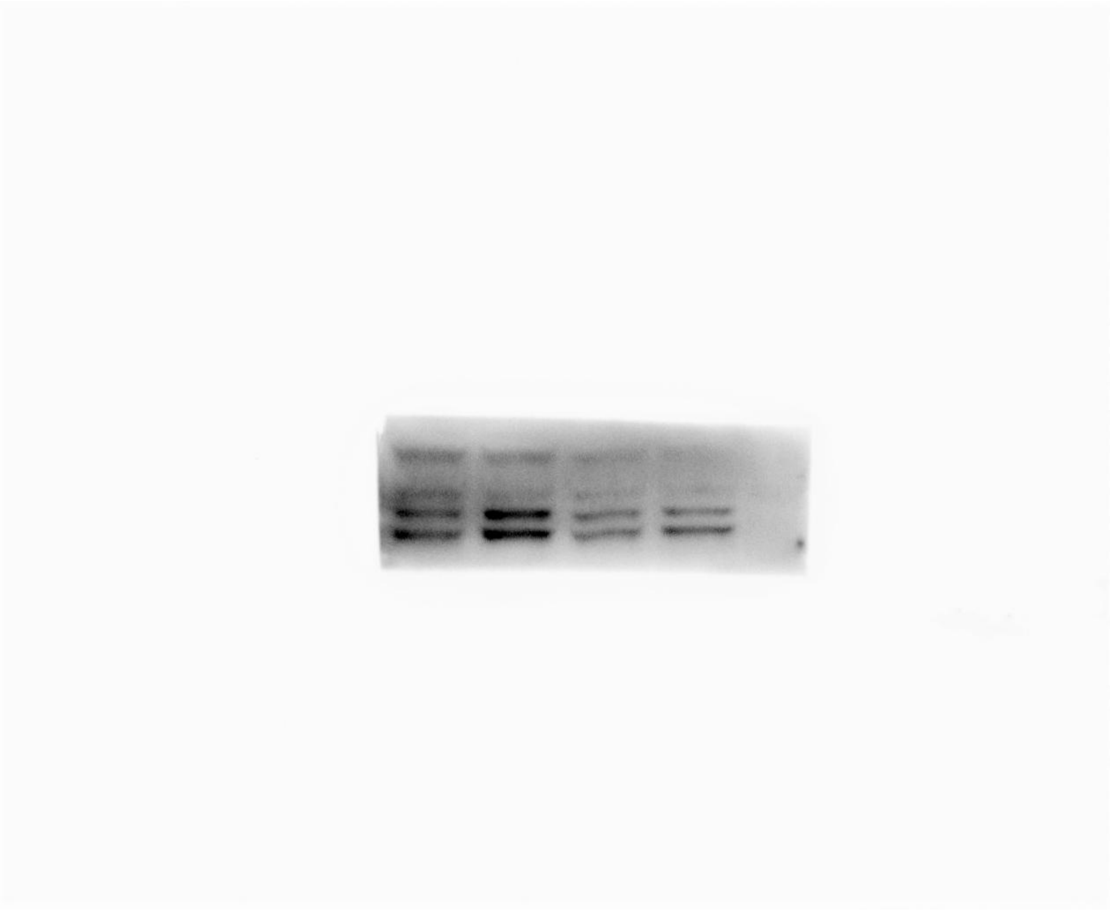

113.fig.S4D-1-p-erk

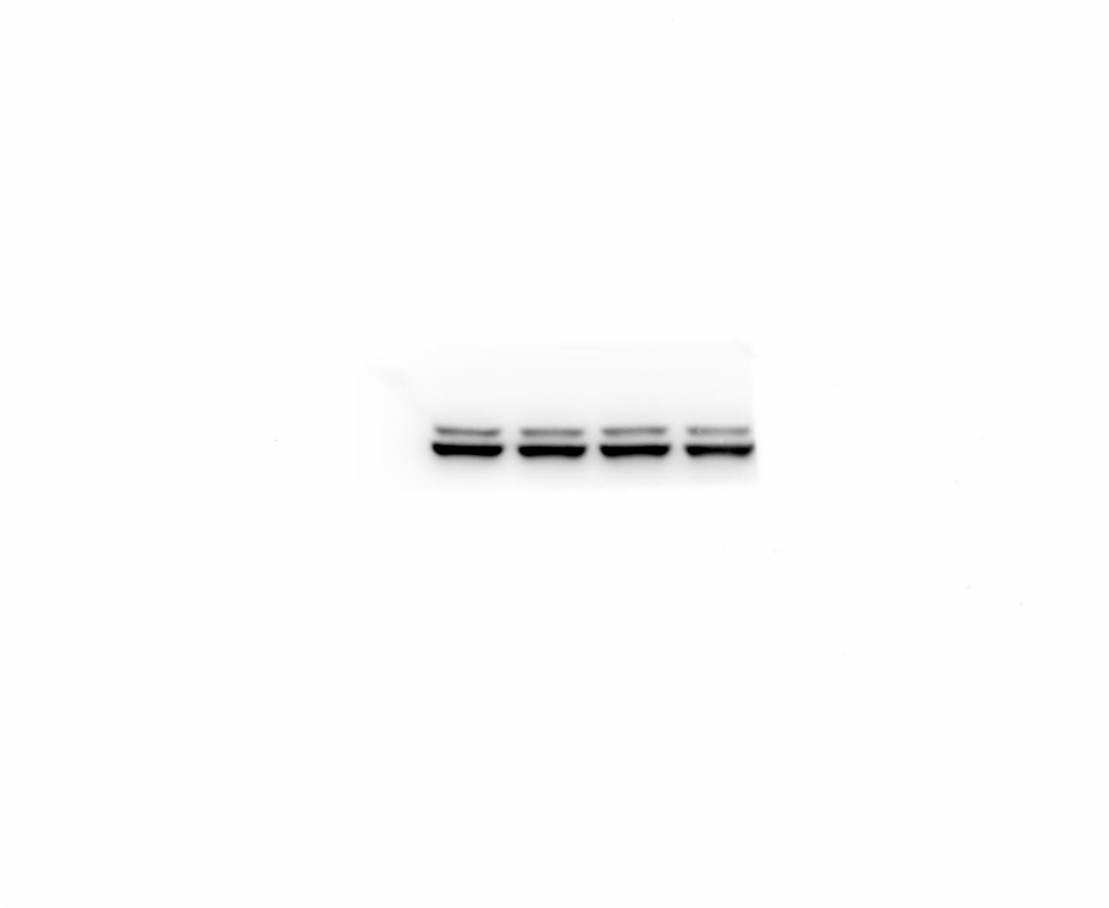

114.fig.S4D-2-erk

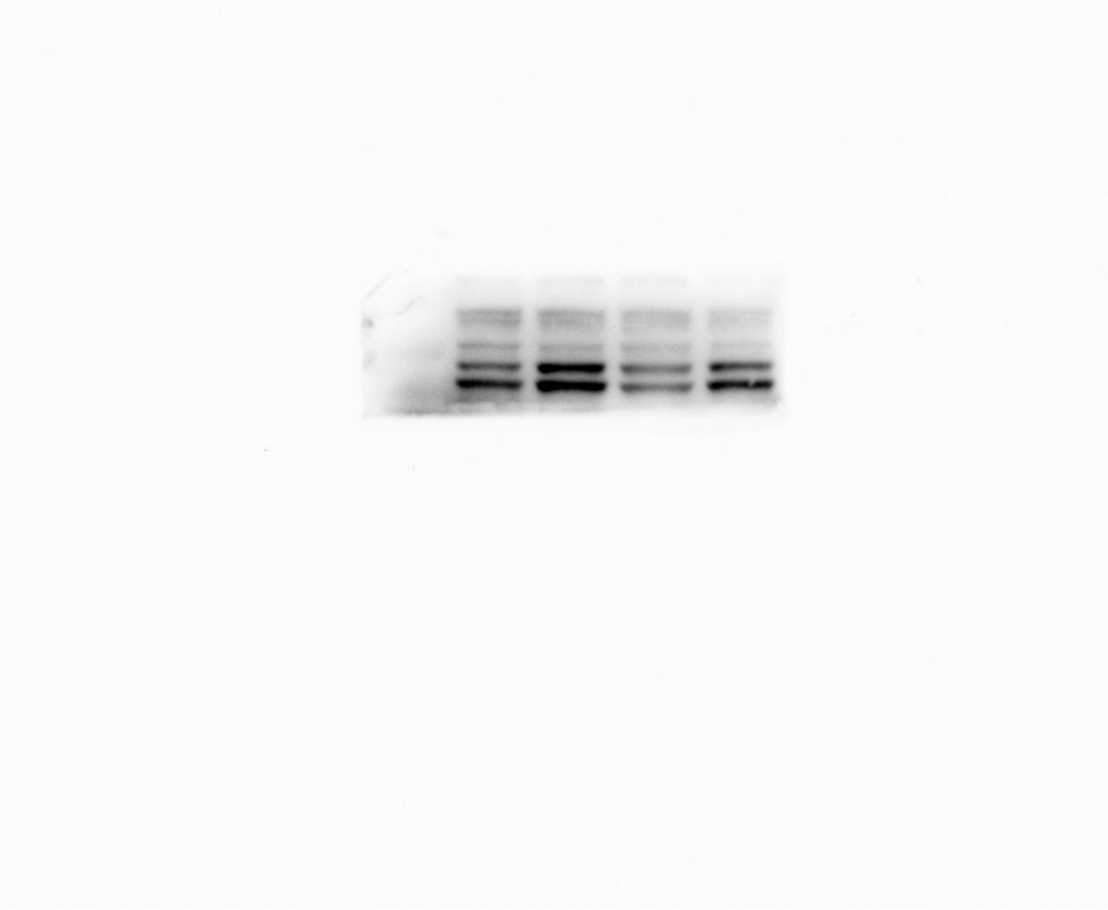

115.fig.S4D-2-p-erk

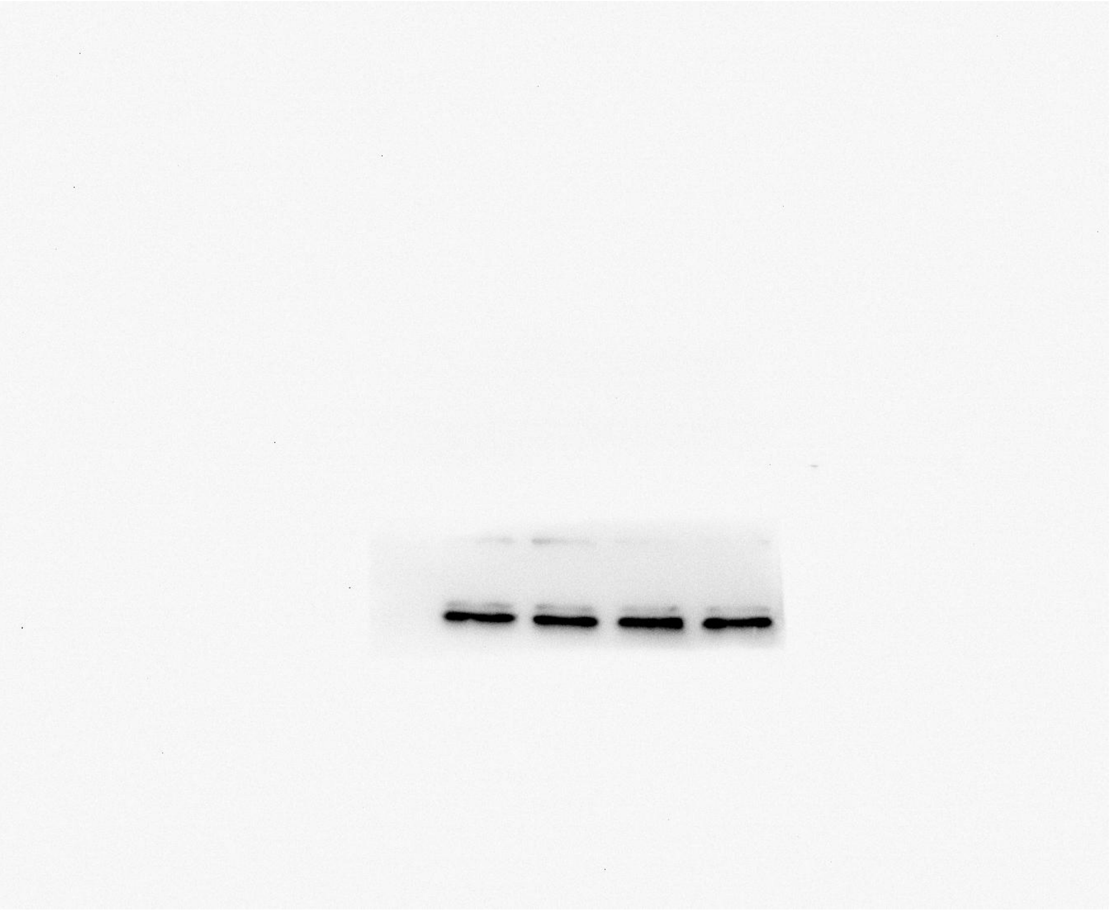

116.fig.S4D-3-erk

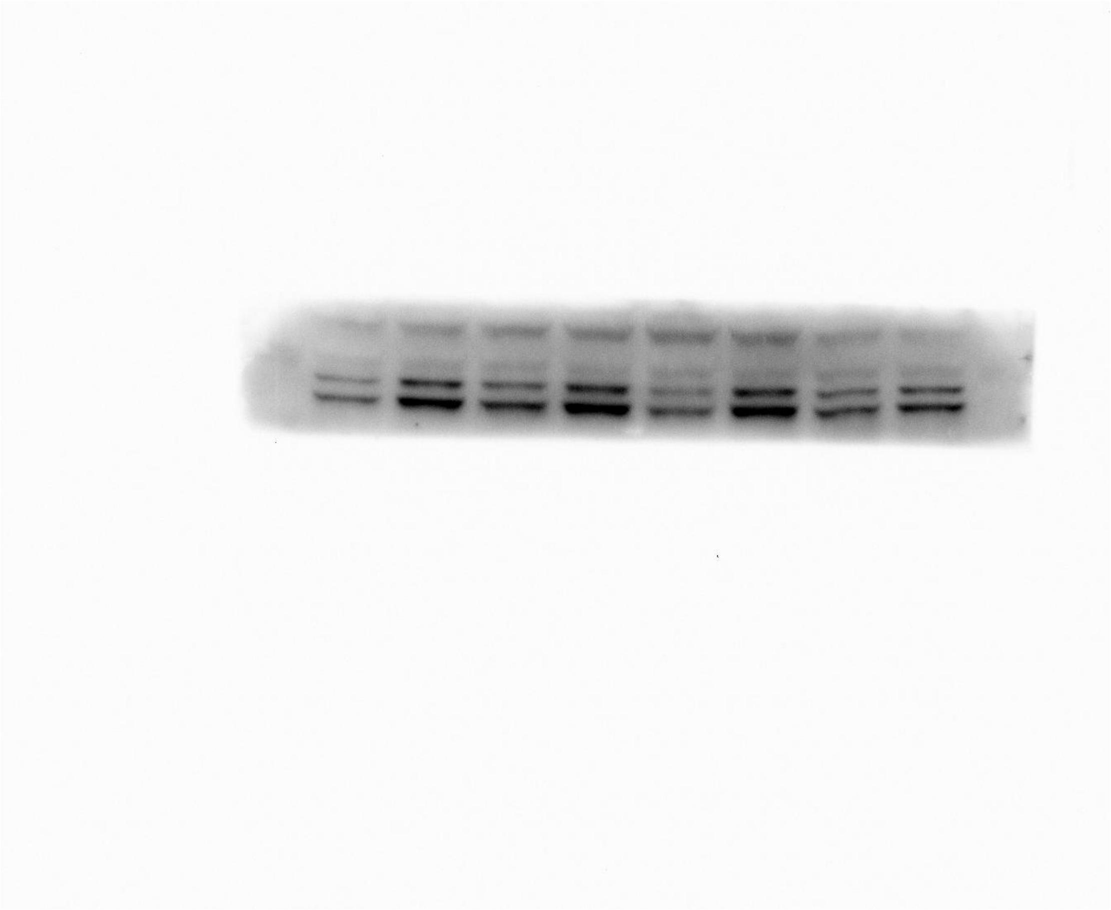

117.fig.S4D-3-p-erk-right

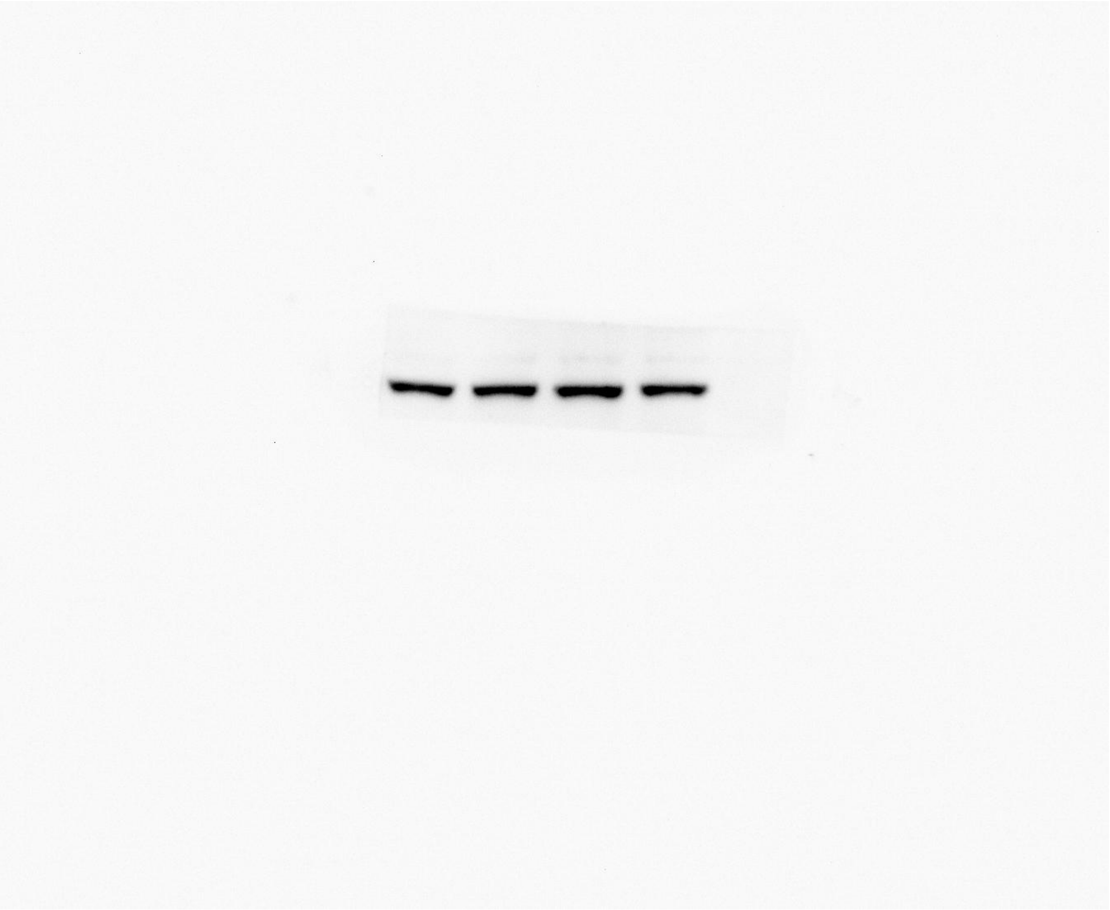

118.fig.S4D-1-mek

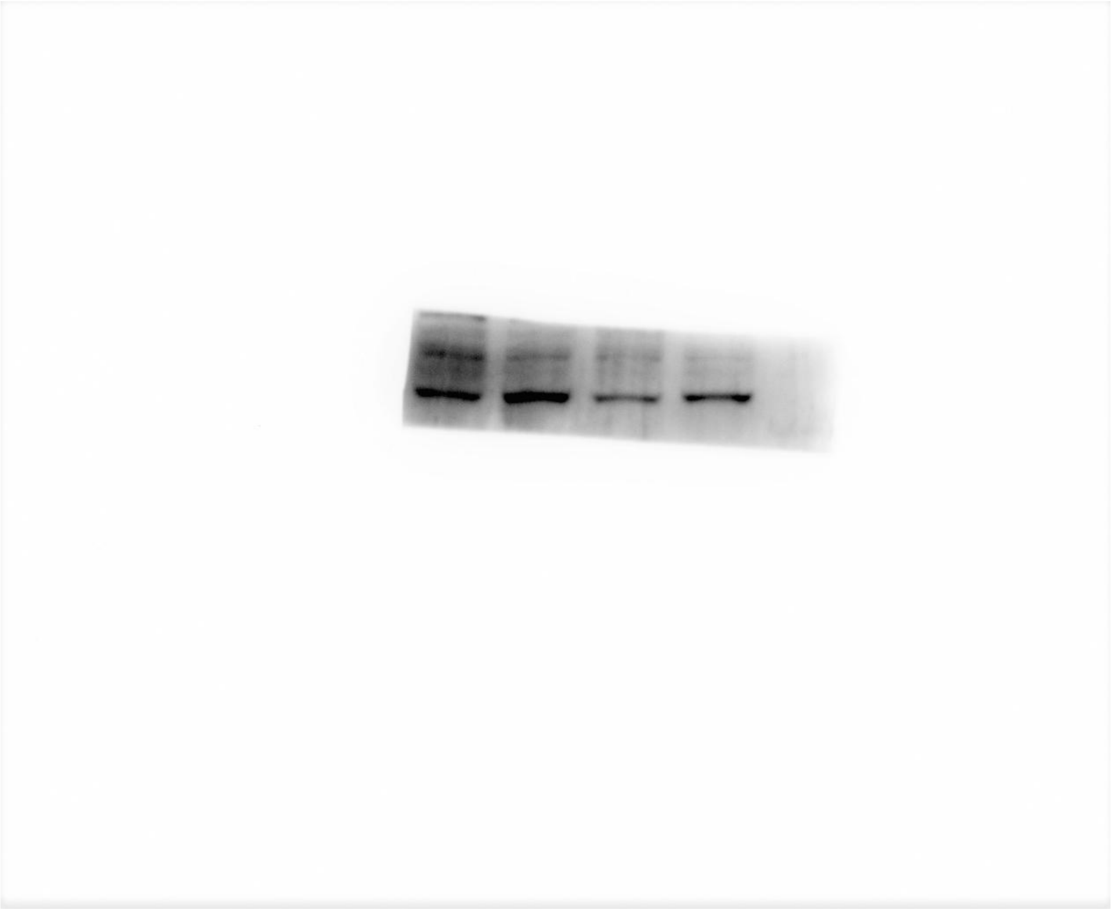

119.fig.S4D-1-p-mek

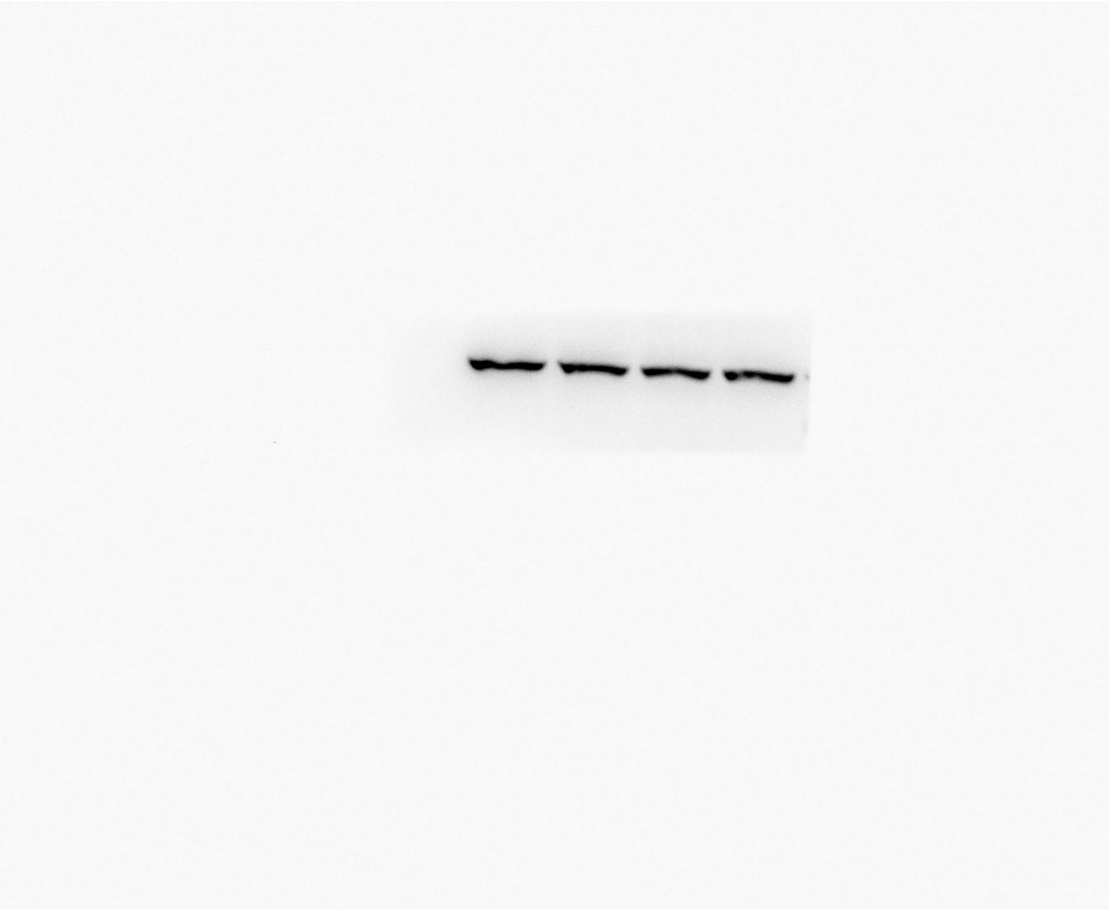

120.fig.S4D-2-mek

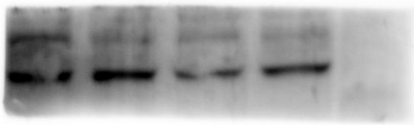

121.fig.S4D-2-p-mek

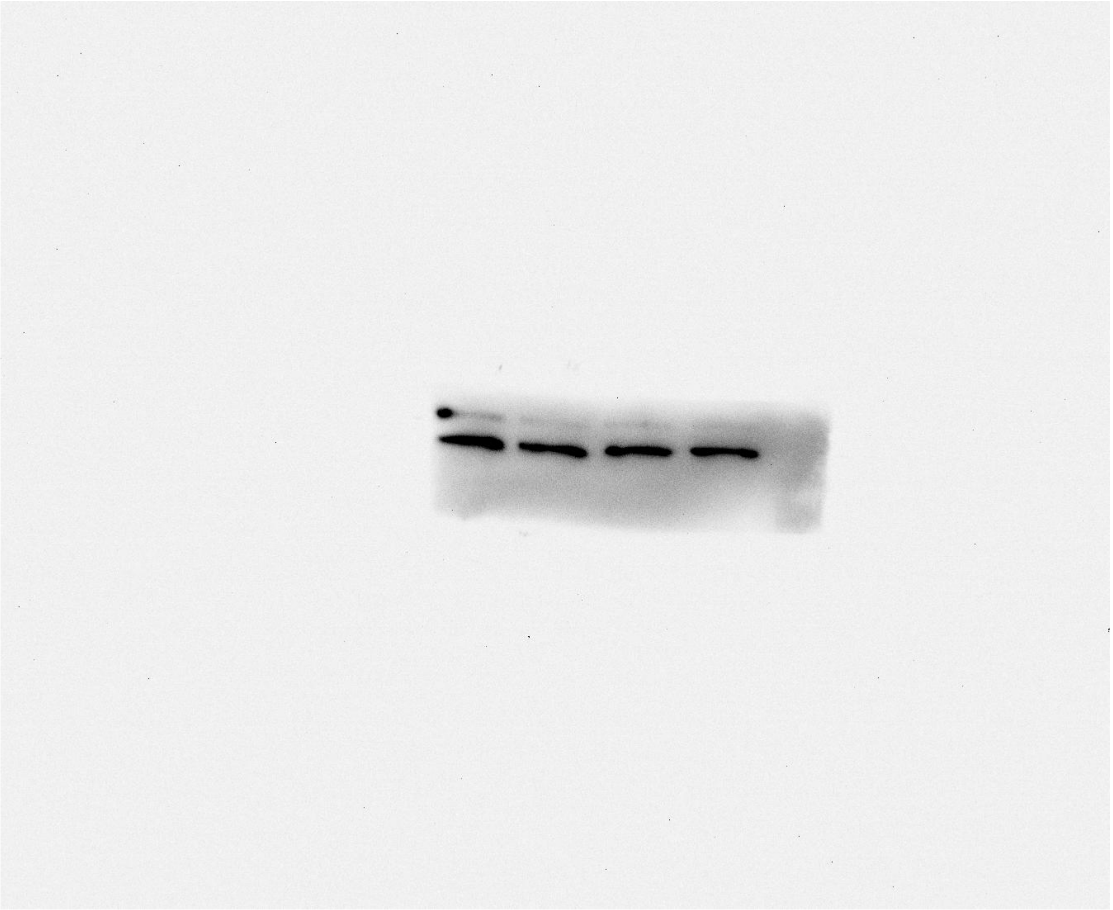

122.fig.S4D-3-mek

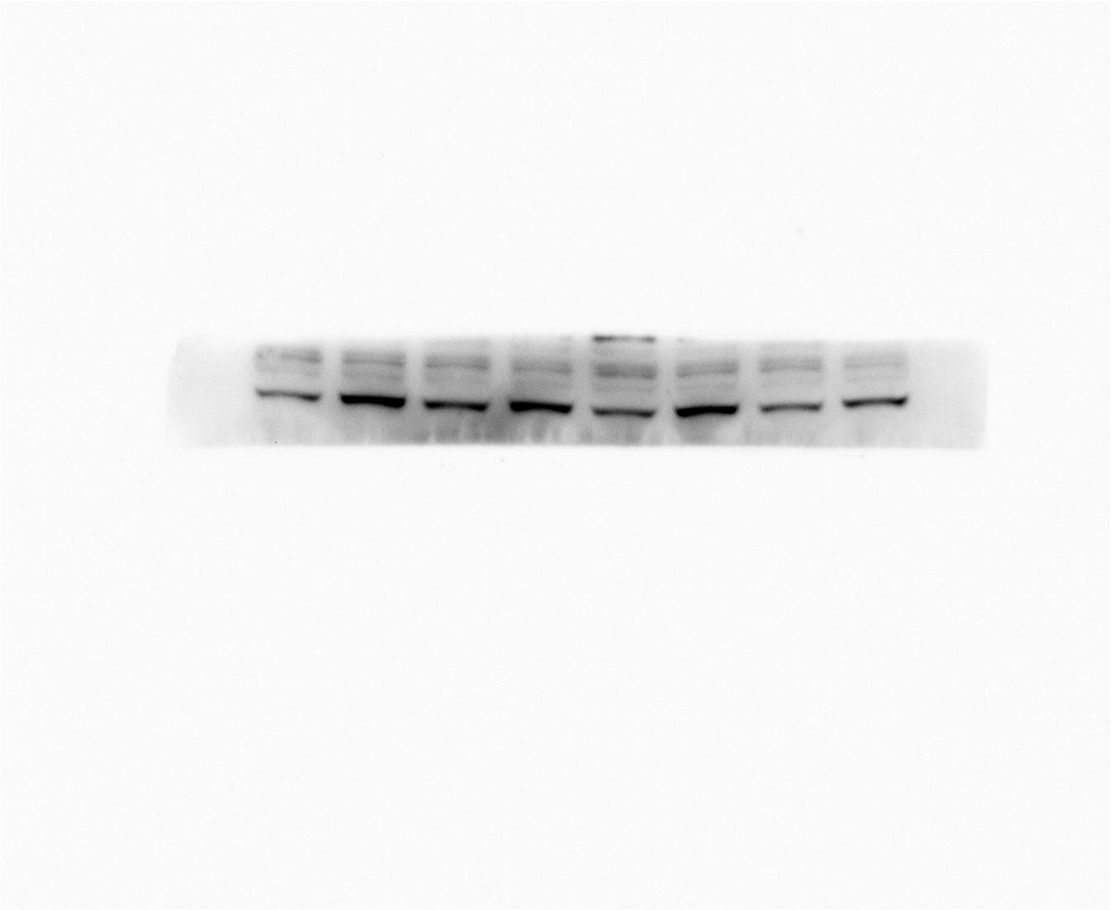

123.fig.S4D-3-p-mek-right
